# Supplementary material for: Systems Vaccinology for a Live Attenuated Tularemia Vaccine Reveals Unique Transcriptional Signatures That Predict Humoral and Cellular Immune Responses
Source: Vaccines (Basel). 2019 Dec 24;8(1):4. doi: 10.3390/vaccines8010004 (PMC7158697; doi:10.3390/vaccines8010004)
Supplement: Supplementary file 1 [file vaccines-08-00004-s001.pdf]

Supplementary Text Manuscript Appendix "‘Systems vaccinology for a live attenuated tularemia vaccine reveals unique transcriptional signatures that predict humoral and cellular immune responses"’
Version 1.0

December 23, 2019

Table of Contents

1 Introduction **8**

2 Supplemental methods **8**

2.1 Study Design 8
2.2 Microarray experiment 8
2.3 Cell mediated immunity experiments 9
2.4 Microarray data preprocessing 10
2.5 Differential gene analysis 11
2.6 Determination of robust gene clusters 11
2.7 Gene set enrichment analysis 11
2.8 Regularized logistic regression analysis 12
2.9 Regularized canonical correlation analysis 13
2.10 Comparisons with viral vaccine microarray studies 14
2.11 Cell mediated immunity analysis 15

3 Supplemental results **15**

4 References **84**

List of Tables

Table 1 Descriptive summary statistics of percent monocyte cells of live PBMCs fold change from baseline by treatment. 16
Table 2 Descriptive summary statistics of percent monocyte cells (CD16+) of live PBMCs fold change from baseline by treatment. 16
Table 3 Descriptive summary statistics of percent monocyte cells (CD16-) of live PBMCs fold change from baseline by treatment. 16
Table 4 Descriptive summary statistics of percent T-cells of live PBMCs fold change from baseline by treatment. 16
Table 5 Descriptive summary statistics of percent T-cells (CD4+) of live PBMCs fold change from baseline by treatment. 16
Table 6 Descriptive summary statistics of percent T-cells (CD4-) of live PBMCs fold change from baseline by treatment. 17
Table 7 Descriptive summary statistics of percent natural killer cells (CD56 bright) of live PBMCs fold change from baseline by treatment. 17
Table 8 Descriptive summary statistics of percent natural killer cells (CD56 bright CD16+) of live PBMCs fold change from baseline by treatment. 17
Table 9 Descriptive summary statistics of percent natural killer cells (CD56 bright CD16-) of live PBMCs fold change from baseline by treatment. 17
Table 10 Descriptive summary statistics of percent natural killer cells (CD56 dim CD16-) of live PBMCs fold change from baseline by treatment. 17
Table 11 Descriptive summary statistics of percent B-cells of live PBMCs fold change from baseline by treatment. 18
Table 12 Descriptive summary statistics of percent T-cells (CD4+ CCR5 high) of live PBMCs fold change from baseline by treatment. 18
Table 13 Descriptive summary statistics of percent T-cells (CD4- CCR5 high) of live PBMCs fold change from baseline by treatment. 18
Table 14 Descriptive summary statistics of percent B-cells (CCR5 high) of live PBMCs fold change from baseline by treatment. 18
Table 15 Descriptive summary statistics of percent T-cells (CD4+ CD69 high) of live PBMCs fold change from baseline by treatment. 18
Table 16 Descriptive summary statistics of percent T-cells (CD4+-CD69 high) of live PBMCs fold change from baseline by treatment. 19
Table 17 Descriptive summary statistics of percent natural killer cells (CD56 bright CD69 high) of live PBMCs fold change from baseline by treatment. 19
Table 18 Descriptive summary statistics of percent natural killer cells (CD56 dim CD16- CD69 high) of live PBMCs fold change from baseline by treatment. 19
Table 19 Descriptive summary statistics of percent B-cells (CD69 high) of live PBMCs fold change from baseline by treatment. 19
Table 20 Descriptive summary statistics of percent T-cells (CD4+ HLA-DR+) of live PBMCs fold change from baseline by treatment. 19

Table 21 Descriptive summary statistics of percent T-cells (CD4- HLA-DR+) of live PBMCs fold change from baseline by treatment. 20
Table 22 Descriptive summary statistics of monocyte cells (CD86-) mean fluorescence intensity fold change from baseline by treatment. 20
Table 23 Descriptive summary statistics of monocyte cells (CD16+ CD86-) mean fluorescence intensity fold change from baseline by treatment. 20
Table 24 Descriptive summary statistics of monocyte cells (CD16- CD86-) mean fluorescence intensity fold change from baseline by treatment. 20
Table 25 Descriptive summary statistics of monocyte cells (CCR5-) mean fluorescence intensity fold change from baseline by treatment. 20
Table 26 Descriptive summary statistics of monocyte cells (CD16+ CCR5-) mean fluorescence intensity fold change from baseline by treatment. 21
Table 27 Descriptive summary statistics of monocyte cells (CD16- CCR5-) mean fluorescence intensity fold change from baseline by treatment. 21
Table 28 Descriptive summary statistics of T-cells (CCR5-) mean fluorescence intensity fold change from baseline by treatment. 21
Table 29 Descriptive summary statistics of T-cells (CD4+ CCR5-) mean fluorescence intensity fold change from baseline by treatment. 21
Table 30 Descriptive summary statistics of T-cells (CD4- CCR5-) mean fluorescence intensity fold change from baseline by treatment. 21
Table 31 Descriptive summary statistics of natural killer cells (CD56 bright CCR5-) mean fluorescence intensity fold change from baseline by treatment. 22
Table 32 Descriptive summary statistics of natural killer cells (CD56 bright CD16+ CCR5-) mean fluorescence intensity fold change from baseline by treatment. 22
Table 33 Descriptive summary statistics of natural killer cells (CD56 bright CD16- CCR5-) mean fluorescence intensity fold change from baseline by treatment. 22
Table 34 Descriptive summary statistics of T-cells (CD4- CD69-) mean fluorescence intensity fold change from baseline by treatment. 22
Table 35 Descriptive summary statistics of natural killer cells (CD56 bright CD69-) mean fluorescence intensity fold change from baseline by treatment. 22
Table 36 Descriptive summary statistics of natural killer cells (CD56 bright CD16+ CD69-) mean fluorescence intensity fold change from baseline by treatment. 23
Table 37 Descriptive summary statistics of natural killer cells (CD56 bright CD16- CD69-) mean fluorescence intensity fold change from baseline by treatment. 23
Table 38 Descriptive summary statistics of natural killer cells (CD56 dim CD16- CD69-) mean fluorescence intensity fold change from baseline by treatment. 23
Table 39 Descriptive summary statistics of B-cells (CD69-) mean fluorescence intensity fold change from baseline by treatment. 23
Table 40 Descriptive summary statistics of monocyte cells (HLA-DR) mean fluorescence intensity fold change from baseline by treatment. 23
Table 41 Descriptive summary statistics of monocyte cells (CD16+ HLA-DR) mean fluorescence intensity fold change from baseline by treatment. 24

Table 42 Descriptive summary statistics of monocyte cells (CD16- HLA-DR) mean fluorescence intensity fold change from baseline by treatment. 24
Table 43 Descriptive summary statistics of Human platelet-derived growth factor concentration fold change from baseline by treatment. 24
Table 44 Descriptive summary statistics of Human Interleukin-1 beta concentration fold change from baseline by treatment. 24
Table 45 Descriptive summary statistics of Human Interleukin-1 receptor antagonist concentration fold change from baseline by treatment. 24
Table 46 Descriptive summary statistics of Human Interleukin-2 concentration fold change from baseline by treatment. 25
Table 47 Descriptive summary statistics of Human Interleukin-4 concentration fold change from baseline by treatment. 25
Table 48 Descriptive summary statistics of Human Interleukin-5 concentration fold change from baseline by treatment. 25
Table 49 Descriptive summary statistics of Human Interleukin-6 concentration fold change from baseline by treatment. 25
Table 50 Descriptive summary statistics of Human Interleukin-7 concentration fold change from baseline by treatment. 25
Table 51 Descriptive summary statistics of Human Interleukin-8 concentration fold change from baseline by treatment. 26
Table 52 Descriptive summary statistics of Human Interleukin-9 concentration fold change from baseline by treatment. 26
Table 53 Descriptive summary statistics of Human Interleukin-10 concentration fold change from baseline by treatment. 26
Table 54 Descriptive summary statistics of Human Interleukin-12 (Lupus Ku autoantigen protein p70) concentration fold change from baseline by treatment. 26
Table 55 Descriptive summary statistics of Human Interleukin-13 concentration fold change from baseline by treatment. 26
Table 56 Descriptive summary statistics of Human Interleukin-15 concentration fold change from baseline by treatment. 27
Table 57 Descriptive summary statistics of Human Interleukin-17 concentration fold change from baseline by treatment. 27
Table 58 Descriptive summary statistics of Human Eotaxin concentration fold change from baseline by treatment. 27
Table 59 Descriptive summary statistics of Human basic fibroblast growth factor concentration fold change from baseline by treatment. 27
Table 60 Descriptive summary statistics of Human granulocyte-colony stimulating factor concentration fold change from baseline by treatment. 27
Table 61 Descriptive summary statistics of Human granulocyte macrophage-colony stimulating factor concentration fold change from baseline by treatment. 28
Table 62 Descriptive summary statistics of Human interferon gamma concentration fold change from baseline by treatment. 28

Table 63 Descriptive summary statistics of Human Interferon gamma-induced protein 10 concentration fold change from baseline by treatment. 28
Table 64 Descriptive summary statistics of Human Monocyte chemoattractant protein-1 (Monocyte Chemotactic and Activating Factor) concentration fold change from baseline by treatment. . 28
Table 65 Descriptive summary statistics of Human Macrophage Inflammatory Protein-1 alpha concentration fold change from baseline by treatment. 28
Table 66 Descriptive summary statistics of Human Macrophage Inflammatory Protein-1 beta concentration fold change from baseline by treatment. 29
Table 67 Descriptive summary statistics of Human RANTES concentration fold change from baseline by treatment. 29
Table 68 Descriptive summary statistics of Human Tumor necrosis factor - alpha concentration fold change from baseline by treatment. 29
Table 69 Descriptive summary statistics of Human Vascular endothelial growth factor concentration fold change from baseline by treatment. 29
Table 70 Descriptive summary statistics of natural killer cells (CD56 dim CD16- CCR5-) mean fluorescence intensity fold change from baseline by treatment. 29
Table 71 Descriptive summary statistics of B-cells (CCR5-) mean fluorescence intensity fold change from baseline by treatment. 30
Table 72 Descriptive summary statistics of T-cells (CD69-) mean fluorescence intensity fold change from baseline by treatment. 30
Table 73 Descriptive summary statistics of T-cells (CD4+ CD69-) mean fluorescence intensity fold change from baseline by treatment. 30
Table 74 Differentially expressed genes (Combined Study Groups, Day 0 vs. 1) 31
Table 75 Differentially expressed genes (Combined Study Groups, Day 0 vs. 2) 35
Table 76 Differentially expressed genes (Combined Study Groups, Day 0 vs. 7) 42
Table 77 Differentially expressed genes (Combined Study Groups, Day 0 vs. 14) 51
Table 78 Differentially expressed genes (vaccine study comparisons, DVC-LVS/USAMRIID-LVS (Tularemia), Day 2 vs. 0) 53
Table 79 Differentially expressed genes (vaccine study comparisons, DVC-LVS/USAMRIID-LVS (Tularemia), Day 7 vs. 0) 55
Table 80 Differentially expressed genes (vaccine study comparisons, YF-17D (Yellow Fever), Day 3 vs. 0) 56
Table 81 Differentially expressed genes (vaccine study comparisons, YF-17D (Yellow Fever), Day 7 vs. 0) 60
Table 82 Differentially expressed genes (vaccine study comparisons, TIV (Influenza), Day 7 vs. 0) . 62
Table 83 Differentially expressed genes (vaccine study comparisons, LAIV (Influenza), Day 3 vs. 0) . 63
Table 84 Differentially expressed genes (vaccine study comparisons, LAIV (Influenza), Day 7 vs. 0) . 65
Table 85 Differentially expressed genes (vaccine study comparisons, DVC-LVS/USAMRIID-LVS (Tularemia) Day 2 vs. YF-17D (Yellow Fever) Day 3) 65
Table 86 Differentially expressed genes (vaccine study comparisons, DVC-LVS/USAMRIID-LVS (Tularemia) Day 7 vs. YF-17D (Yellow Fever) Day 7) 69

Table 87 Differentially expressed genes (vaccine study comparisons, DVC-LVS/USAMRIID-LVS (Tularemia) Day 2 vs. TIV (Influenza) Day 3) . . . 69
Table 88 Differentially expressed genes (vaccine study comparisons, DVC-LVS/USAMRIID-LVS (Tularemia) Day 7 vs. TIV (Influenza) Day 7) . . . 70
Table 89 Differentially expressed genes (vaccine study comparisons, DVC-LVS/USAMRIID-LVS (Tularemia) Day 2 vs. LAIV (Influenza) Day 3) . . . 71
Table 90 Differentially expressed genes (vaccine study comparisons, DVC-LVS/USAMRIID-LVS (Tularemia) Day 7 vs. LAIV (Influenza) Day 7) . . . 72
Table 91 Combination of genes differentiating between responders and non-responders (Microagglutination titer, Day 2) . . . 74
Table 92 Combination of genes differentiating between responders and non-responders (Microagglutination titer, Day 7) . . . 76
Table 93 Canonical correlation analysis summary statistics . . . 76

List of Figures

Figure 1 Scatter plot of log_2 fold changes of significant genes (DVC-LVS vs. USAMRIID-LVS, Day 7, 14) 77
Figure 2 Gene cluster dendrogram with bootstrap probabilities (Combined Study Groups, Day 1-14) 78
Figure 3 Gene cluster time trends of baseline log_2 fold change by study group (Combined Study Groups, Day 1-14) 79
Figure 4 Gene cluster time trends of baseline log_2 fold change by study group (Combined Study Groups, Day 1-14) 80
Figure 5 Gene cluster time trends of baseline log_2 fold change by study group (Combined Study Groups, Day 1-14) 81
Figure 6 Time trends of mean log_2 fold change and associated 95% bootstrap confidence intervals for gene clusters identified in subject-level heatmaps. 82
Figure 7 Number of subjects that pass a certain immune response cut off by assay. 83
Figure 8 Canonical correlation scree plots. 84

1 Introduction

This appendix provides supporting information for the manuscript entitled "'Systems vaccinology for a live attenuated tularemia vaccine reveals unique transcriptional signatures that predict humoral and cellular immune responses"'.

2 Supplemental methods

2.1 Study Design

This laboratory substudy was a systems biology study of the human response to two lots of tularemia vaccines assessed in a phase 2 clinical trial [1]. Tularemia is caused by *Franciscella tularensis*, a gram-negative bacterium that has been weaponized as an aerosol. For protection of personnel conducting biodefense research, the United States Army required clinical evaluation of a new lot of tularemia live vaccine strain manufactured in accordance with Current Good Manufacturing Processes. To assess this, a phase 2 randomized clinical trial was conducted comparing the new lot (DVC-LVS) produced by DynPort Vaccine Company to the existing vaccine that has been in use by the United States Army Medical Research Institute of Infectious Diseases (USAMRIID-LVS) for decades. The vaccines were delivered by scarification to 228 participants in the main study, which evaluated vaccine safety, reactogenicity, take and Tularemia-specific microagglutination titer on Days 0 (baseline), 8, 14, 28, 56, and 180. For this substudy, blood samples were obtained for 42 healthy male and female subjects aged 18 to 45 years old, enrolled in [1]. Laboratory assessments on Days 0, 1, 2, 7, and 14 included gene expression based on PBMC RNA using microarrays, serum cytokine/chemokine concentration measurements, and immune cell phenotyping (DC/monocyte/lymphocyte cells). T-cell activation was evaluated on Days 0, 14, 28, 56, and 180. Day 180 also included an assessment of T-cell proliferation.

2.2 Microarray experiment

Affymetrix High-throughput (HT) Perfect Match (PM) Array GeneChips were run in a 96 array plate configuration (96 samples per HT array). Each individual array contained 54,715 probe sets and 536,460 perfect match (PM) probes (on average 9.8 probes per probe set). HT PM Array Plates only contain PM probes. Mismatch probes (MM) are not included. Three 96 HT array plates were run producing data for 205 samples (the last 96 HT array contained data for 13 samples). Plates were run on three different days (10/11/2011, 11/8/2011, and 11/15/2011). For five samples array data was not produced: subject T02HC051 was missing Day 1; T02HC115 was missing Days 2, 7, and 14; T02HC118 was missing Day 0. All of the other 39 subjects had array data for all 5 time points (Day 0, 1, 2, 7, 14).

2.3 Cell mediated immunity experiments

Phenotyping of immune cell subpopulations This assay included 3 panels. It was performed pre-vaccination (Day 0), and at Days 1, 2, 7 and 14 post-vaccination. Results were measured in percent live PBMCs as well as mean fluorescence intensity.

For the dendritic cell panel, samples were first gated to exclude dead cells, debris, and doublets. Total Dendritic cells (DCs) were then defined as linage negative (CD3-CD14-CD16-CD19-CD20-CD56-) and HLADR+. DCs were then divided into myeloid dendritic cells (mDCs) and plasmacytoid dendritic cells (pDCs) subset based on CD11c and CD123 expression, respectively. mDCs were then further subdivided based on BDCA1 expression. All populations were then analyzed for CCR7, CD86, CD11b, and HLADR expression.

For the monocyte panel, samples were first gated to exclude dead cells, debris, and doublets and then B-cells and T-cells were excluded by CD19 and CD3 expression. HLADR+ cells were then defined as monocytes. Monocytes were then subdivided into CD14+ CD16- classical monocytes, CD14+ CD16+ intermediate monocytes, and CD14 dim CD16+ non-classical monocytes. All populations were then analyzed for CD86, CCR5, and HLADR expression.

For the lymphocyte panel, samples were first gated to exclude dead cells, debris, and doublets. CD14+ cells are excluded and CD14- cells were divided into CD3+ (T cells), CD19+ (B cells), and CD3- CD19- subsets. CD3+ cells were then divided into CD4+ and CD4- subsets and further analyzed for HLADR expression. The CD3-CD19- population was divided into CD56 bright (CD56 high CD16-) and CD56 dim (CD56 mid CD16+) natural killer (NK) cell subsets. All populations were then analyzed for CCR5 and CD69 expression. Expression of CCR7, CD86, CD11b, HLADR, CCR5 and CD69 was reported as mean fluorescence intensity (MFI). Background fluorescence was subtracted using isotype controls for all antibodies but anti HLADR.

T-Cell activation by phenotyping Phenotyping of T-cell activation was performed with whole blood samples at Day 0 (baseline), and Days 1, 2, 7, 14, 28 and 56 post-vaccination. Lymphocytes were identified based on forward scatter (FSC) and side scatter (SSC) properties. CD3+CD4+CD8- cells and CD3+CD4-CD8+ cells were defined as CD4 and CD8 T-cells. Activated T-cells were defined by co-expression of CD38 and HLA-DR (surface staining) or co-expression of Ki67 and Bcl-2 (intracellular staining) separately. Results were measured in percent live PBMCs.

T-cell proliferation assay The CFSE proliferation assay was performed using PBMCs from participants at Day 180 post-vaccination. Cells were labeled with CFSE and stimulated for 6 days with formalin-inactivated Tularemia Type B strain LVS or Type A strain SCHU S4 and then analyzed by

FACS. Lymphocytes, CD4 and CD8 T-cells were gated as described above. T-cell proliferation potential was determined as percent of CFSE low cells on CD4 and CD8 T cells separately and was normalized by subtracting percent of CFSE low cells obtained for negative (unstimulated) controls.

Serum cytokines/chemokines Human plasma cytokines were analyzed for Days 0, 1, 2, 7 and 14 using the Bio-Plex Pro Human Cytokine 27-plex Assay (Bio-Rad) according to manufacturer's instruction and are represented as pg/ml.

2.4 Microarray data preprocessing

Probe set annotations for HT HG-U133 Plus PM GeneChips were downloaded from the Affymetrix website (Release 34, 10/23/13). Due to missing MM probes, MAS 5.0-based average background and scale factors could not be calculated as outlined in the SAP. Instead, a median scaling factor for each sample was calculated by first calculating a scaling factor for each probe on the log_2 scale (median across all samples divided by probe intensity) followed by calculating the median of all probe scaling factors per sample. High values indicate that a library had typically lower probe values and vice versa. RNA quality and transcript truncation was evaluated by using β-actin and GAPDH housekeeping control probe sets that target different regions of the same gene (3$'$ end of transcript, middle (M) portion of the transcript, and 5$'$ end of transcript). 3$'$/5$'$ ratios and M/5$'$ ratios were calculated for each individual array. Hybridization and overall signal quality for each array was evaluated using 4 Affymetrix spike-in probe sets (BioB, BioC, BioD, CreX) that are added to the experiment with increasing RNA concentrations (as listed from low to high). Median intensity was used to summarize intensities per probe set and log_2 value of the ratio was reported. The slope (proxy for hybridization strength) was estimated for each sample by fitting a linear regression model. RNA-degradation was inspected by plotting average probe intensities in the 5' to 3' direction (the first 8 probes were included per probe set). The RNA degradation slope was estimated for each sample by fitting a linear regression model. The Robust Multichip Average (RMA) algorithm (Irizarry *et al.*, 2003) was used to obtain background-corrected and quantile-normalized probe-level intensities. Probe and probe-level log_2 intensity distributions were investigated using boxplots, probability density function (PDF) plots, and log expression (RLE) boxplots (Brettschneider *et al.*, 2008). The ComBat algorithm [2] using the parametric empirical Bayes option was applied to adjust the probe-level data for batch effects. Extreme outliers were removed from the probe level data and background correction, quantile normalization, and batch correction was rerun in the specified order. Next, probe sets with a sample coefficient of variation ($\hat{CV} = \frac{S}{\hat{x}}$) in the lower 25% quantile were filtered out.

2.5 Differential gene analysis

A two-sided paired t-test was applied to identify significantly up/down-regulated genes from baseline (Day 0 versus Day 1, 2, 7, 14). The paired test was carried out separately for each study group as well as for combined study groups. In addition, log_2 fold changes per study visit were compared between study groups using a two-sided Welch's t-test. To compensate for multiple testing, the false discovery rate (FDR) which controls the false positive rate among significantly differentially expressed genes was calculated using the *qvalue* R package. Genes with a q-value $\leq$ 0.05 and a fold change of $\geq$ 1.5-fold (up or down regulation) were deemed to be significantly differentially expressed.

2.6 Determination of robust gene clusters

log_2 fold changes of genes that were differentially expressed for a certain study group at any post baseline day were used as input for gene clustering. Uncentered Pearson correlation distance was used as distance measure. Pairwise distances between log_2 changes were calculated separately for each vaccine group and post baseline day (Day 1, 2, 7, 14) as well as for all study days (Day 1-14). Clusters were obtained using the hierarchical complete linkage clustering algorithm. To evaluate robustness of gene clusters, multiscale bootstrapping (Suzuki and Shimodaira, 2006) was carried out using varying dataset sizes (0.4*N, 0.5*N, 0.6*N, 0.7*N, 0.8*N, 0.9*N, 1*N, 1.1*N, 1.2*N, 1.3*N; where N stands for the respective dataset size). For each dataset size bin, 1,000 bootstrap samples were obtained, and bootstrap probabilities and p-values were calculated. The unbiased bootstrap probability cut-off was set to 0.05 and the maximum distance to form a significant cluster was set to 0.5 (equivalent to minimum uncentered Pearson correlation of 0.5). Clusters that were formed at larger distances were excluded.

2.7 Gene set enrichment analysis

Gene set enrichment analysis was performed using the Java implementation (Version 2.1.0, downloaded 01/23/2015 from http://www.broadinstitute.org/gsea) of the GSEA algorithm (GSEAPreranked method) to detect enriched gene sets [3]. Ensemble genes were grouped and analyzed based on 8,101 known gene sets obtained from the KEGG database (Version 70.0 (06/09/2014), [4]) and MSigDB (Version 4.0 (05/31/2013), [5]). The MSigDB gene set collection was comprised of GO Biological Processes, GO Cellular Components, GO Molecular Functions, Reactome Pathway, BioCarta Pathway, Chemical/-Genetic Perturbations, and Immunologic Signature gene sets. GSEA analysis was carried out separately for each post-vaccination day, comparison group (post-vaccination vs. baseline for DVC-LVS, post-vaccination vs. baseline for USAMRIID-LVS, and post-vaccination vs. baseline for the combined groups, as well as DVC-LVS vs. USAMRIID-LVS for each post-vaccination day). Probe set IDs were converted to Ensembl gene IDs based on Affymetrix HG U133 release 34 probe set annotations. To increase annotation coverage, for genes that did not have Affymetrix Ensembl gene IDs assignments,

if available, common name to gene ID mappings were obtained from the HUGO Gene Nomenclature Committee (www.genenames.org, downloaded 05/12/2015). Ranked lists of Ensembl gene IDs based on decreasing t-statistic were used as input for Chemical/Genetic Perturbations, and Immunologic Signature gene. For all other gene set collections, the absolute t-statistic was used. In either case, before generating the lists, the largest absolute t-statistic was used for Ensembl gene IDs that mapped to multiple probe sets. Except for Chemical/Genetic Perturbations, and Immunologic Signature gene sets which contain directional information, the absolute t-statistic was used. Program specifications were as follows: random seed was specified as 149, collapse was set to false, the minimum and maximum gene set size was not restricted (set to 1 and 10000, respectively), and the number of permutations was set to 10,000. To adjust for testing multiple gene sets per category type, the Benjamini-Hochberg procedure was applied to each list. For Chemical/Genetic Perturbation and Immunologic Signature collections, with many more gene sets compared to the other gene set collections, a FDR < 0.01 was applied to determine significant enrichment. For all other collections, a cut off of FDR < 0.1 was used. Enrichment trends across post-vaccination days and comparison groups were visualized using heatmaps and binary Jaccard-distance based complete-linkage hierarchical clustering.

2.8 Regularized logistic regression analysis

As there is no *a priori* knowledge about the correlates of protection for these vaccines, responders, non-responders, and subjects with high baseline were arbitrarily defined. The positive cut off for immune response thresholds for responders based on the percentage of activated CD4+, CD8+ T-cells and microagglutination titer was defined as three standard deviations (SD) above the mean of all baseline samples. Concurrently, a minimum sample size of 10 subjects in either response group (responders and non-responders) was required. The minimum subject criterion was met for responders and non-responders when combining vaccine study arms based on CD8+ T-cell activation and microagglutination titer but not for percent activated CD4+ T-cells. As the SAP allowed for flexibility in the choice of the cut off, the numbers of responders and non-responders in the combined data set were plotted for different SD cut offs to see if an alternative cut off can be identified that meets the minimum subject requirement. Based on the CD4+ plot, a cut off of 2 SD was chosen to define a positive CD4+ response. The classification for subjects with persistent T-cell responses (3 SD above the baseline mean at Day 7, 14, and 28) versus transient T-cell responses (3 SD above the baseline mean at Day 7 and 14 but below this cut off at Day 28) did not meet the minimum number of required subjects for CD4+ and CD8+ for any SD cut off. Thus, the comparison of transient versus persistent subjects was not carried out. The following criteria were used to define assay-specific responders and non-responders:

1. classification based on Tularemia-specific microagglutination titer
    (a) responder: a subject in which tularemia vaccine induces an increase in Tularemia-specific log-transformed microagglutination titer that is 3 SD above the mean of all log-transformed baseline values, at any of the following time points: Days 14 or 28.

(b) non-responder: a subject in which tularemia vaccine does not induce an increase in tularemia specific log-transformed microagglutination titer that is 3 SD above the mean of all log-transformed baseline values, at any of the following time points: Days 14 or 28.

(c) subject with high baseline: a subject with a log-transformed baseline value that is 3 SD above the mean of all log-transformed baseline values.

For each post-vaccination Day (1, 2, 7, 14) and assay-specific classification, a regularized logistic regression model was fit to identify gene responses that distinguish between responders and non responders using the *glmnet* R package (Version 2.0-2). Before fitting the models, the predictor variable set (probe sets) was processed as follows:

1. probe sets corresponding to the same gene (same gene name) were collapsed by retaining the probe set per gene that had the highest absolute mean fold change from baseline at the respective post vaccination-day.
2. collapsed probe sets with an absolute fold change $\geq$ 1.5 were retained and used as predictors for the respective model.

To avoid overfitting (n $<<$ p and collinearity among gene responses) and facilitate variable selection, an elastic net regularization step (combination of L1 Lasso and L2 ridge penalization, α = 0.5) was included as part of the fitting procedure. Five-fold cross validation was used to determine the optimum regularization parameter λ based on the minimum average model deviance across folds. In addition, the mean misclassification error across folds was determined for the optimal λ and provided as additional cross-validation statistic. The five random folds were stratified by positive and negative responders, i.e. the overall proportion of positive/negative responders was maintained for each random fold. The seed was set to 20151121.

2.9 Regularized canonical correlation analysis

To identify patterns that explain associations between changes in gene expression and peak changes in immunogenicity outcomes (IMO), multivariate regularized Canonical Correlation Analysis (CCA) was carried out separately for Days 1, 2, 7, and 14. CCA identifies linear combinations of the original variables (referred to as canonical variates) that maximize the inter-set correlation (here gene and immunogenicity variable sets). Planned immunogenicity variables included peak baseline fold change in %T-cell activation (CD8+ and CD4+ cells), T-cell proliferation at Day 180 (CD8+ and CD4+ cells), and peak baseline fold change in microagglutination titer. T-cell proliferation data was not included as part of the models as proliferation data for 11 subjects was missing (the data had not been collected).
Before fitting the CCA models, the gene expression variable set was processed as follows:

1. probe sets corresponding to the same gene (same gene name) were collapsed by retaining the probe set per gene that had the highest absolute mean fold change from baseline for the respective post vaccination-day.

2. collapsed probe sets with an absolute fold change $\geq$ 1.5 were retained and used as gene variable set for the correlation analysis.

The 5 IMO variables were encoded as follows:

– CD4H: subject-specific peak fold change in %CD3+ CD4+ CD38+ HLA-DR+ T-cells (Days 14, 28, 56, 180).

– CD4B: subject-specific peak fold change in %CD3+ CD4+ BCL2+ KI67+ T-cells (Days 14, 28, 56, 180).

– CD8H: subject-specific peak fold change in %CD3+ CD8+ CD38+ HLA-DR+ T-cells (Days 14, 28, 56, 180).

– CD8B: subject-specific peak fold change in %CD3+ CD8+ BCL2+ KI67+ T-cells (Days 14, 28, 56, 180).

– MAGL: subject-specific peak fold change in Tularemia-specific microagglutination titer (Days 14, 28, 56, 180).

Prior to CCA, immunogenicity variables were transformed using the Box-Cox transformation to make their distributions more symmetric and unimodal (while normality is not required, it optimizes the distributional information used as part of the modeling process). To avoid over fitting (n« g and collinearity among genes) a regularization step was applied. Optimal regularization parameters were estimated using leave-one-out cross validation. Significant canonical variate pairs were identified by manually inspecting scree plots. Individual variables were correlated against their significant canonical variates to obtain canonical loadings. Canonical loadings were squared to obtain the proportion of explained variance. Canonical loadings for each variable set were plotted and variables with low explained variance were filtered out (correlation < 0.5 or explained variance < 25%).

2.10 Comparisons with viral vaccine microarray studies

RMA-normalized expression data obtained from yellow fever (YF-17D) vaccinees (Day 0, 3, 7) as well as trivalent influenza vaccine (TIV) (Day 0, 3, 7), and live attenuated influenza vaccine (LAIV) (Day 0, 3, 7) vaccinees were provided by Emory University. For each vaccine study, probe sets with a coefficient of variation (CV) higher than the lower quartile of all CVs were retained. Due to different Affymetrix array types and non-matching probe information (HG-U133 PM for 10-0019 Tularemia vaccine data and HG133 Plus 2 for YF-17D, TIV, and LAIV microarray data), the mean log_2 fold change across gene sets with the same gene symbol (based on Affymetrix release 34 probe set annotations) was used for the comparative analysis. For each vaccine study and post-vaccination day, a paired-t test was applied to identify genes that were differential expressed from baseline. A Welch t-test was used to identify differential fold changes between vaccine studies (Day 2 responses following DVC-LVS/USAMRIID-LVS vaccination were compared to Day 3 responses following YF-17D, TIV, or LAIV vaccination). The same fold change and FDR cut offs as described in **Section 2.5** were used. For gene set enrichment analysis, gene symbols were mapped to Ensembl gene IDs using Affymetrix HG-U133 PM and Hugo (accessed 05/12/2015) mappings. Only gene symbols represented in the 10-0019 Tularemia expression data set were analyzed using GSEA. For Ensembl gene IDs that mapped to multiple gene symbols, the

largest absolute t statistic was used. Genes were then ranked based on absolute t statistic and GSEA analysis was carried out as described in **Section 2.7**. The DVC/USAMRIID-LVS data was pooled when identifying enriched gene sets as well as DE genes. Tularemia vaccine group membership was retained and displayed in subject-level heatmaps when comparing differential gene lists.

2.11 Cell mediated immunity analysis

MFI values less than 0 after normalization of background fluorescence were set to 0. MFI values of 0 were imputed to 1 for fold calculations only. Similarly, percent cell activation values of 0 were imputed to 0.01 for fold change calculations only. Cytokines with concentrations less than the experimental lower limit of quantification (LLOQ) were imputed to 1/2 LLOQ. A 95% CI for the median measurement as well as median fold change from baseline for each time point and treatment group was obtained for each assay and marker combination using the bootstrap method with 1,000 bootstrap replicates each.

3 Supplemental results

Tables

	DVC-LVS						USAMARIID-LVS						Combined					
Study Visit	n	Min	Q1	Median (95% CI)	Q3	Max	n	Min	Q1	Median (95% CI)	Q3	Max	n	Min	Q1	Median (95% CI)	Q3	Max
Day 1	19	0.65	0.85	0.94 (0.86,1.14)	1.14	1.46	15	0.74	0.92	1.11 (0.93,1.35)	1.39	1.57	34	0.65	0.88	0.98 (0.90,1.14)	1.24	1.57
Day 2	19	0.60	0.98	1.16 (1.02,1.34)	1.36	1.73	16	0.53	0.84	1.03 (0.84,1.24)	1.25	1.45	35	0.53	0.91	1.09 (0.95,1.24)	1.32	1.73
Day 7	19	0.53	0.67	0.80 (0.72,0.92)	0.98	2.07	16	0.33	0.61	0.78 (0.62,1.15)	1.18	2.06	35	0.33	0.62	0.79 (0.76,0.92)	1.11	2.07
Day 14	19	0.54	0.69	0.78 (0.70,0.94)	0.95	1.57	16	0.34	0.64	0.77 (0.63,1.05)	1.08	1.92	35	0.34	0.66	0.78 (0.68,0.94)	1.02	1.92

Table 1: Descriptive summary statistics of percent monocyte cells of live PBMCs fold change from baseline by treatment.

	DVC-LVS						USAMARIID-LVS						Combined					
Study Visit	n	Min	Q1	Median (95% CI)	Q3	Max	n	Min	Q1	Median (95% CI)	Q3	Max	n	Min	Q1	Median (95% CI)	Q3	Max
Day 1	19	0.85	0.94	1.05 (0.96,1.23)	1.26	1.56	15	0.87	1.24	1.32 (1.22,2.05)	2.06	2.38	34	0.85	0.99	1.23 (1.05,1.32)	1.43	2.38
Day 2	19	0.85	1.04	1.28 (1.08,1.72)	1.74	3.16	16	0.84	0.99	1.68 (0.99,1.99)	2.01	2.54	35	0.84	0.99	1.41 (1.16,1.76)	1.81	3.16
Day 7	19	0.52	0.77	0.95 (0.81,1.13)	1.22	2.40	16	0.83	1.09	1.22 (1.10,1.33)	1.37	3.93	35	0.52	0.93	1.13 (0.98,1.26)	1.32	3.93
Day 14	19	0.35	0.63	0.88 (0.66,1.17)	1.17	2.57	16	0.61	0.83	0.99 (0.79,1.47)	1.57	2.68	35	0.35	0.71	0.91 (0.82,1.12)	1.27	2.68

Table 2: Descriptive summary statistics of percent monocyte cells (CD16+) of live PBMCs fold change from baseline by treatment.

	DVC-LVS						USAMARIID-LVS						Combined					
Study Visit	n	Min	Q1	Median (95% CI)	Q3	Max	n	Min	Q1	Median (95% CI)	Q3	Max	n	Min	Q1	Median (95% CI)	Q3	Max
Day 1	19	0.59	0.81	0.95 (0.82,1.12)	1.12	1.47	15	0.72	0.89	1.06 (0.91,1.27)	1.33	1.63	34	0.59	0.83	0.96 (0.88,1.12)	1.23	1.63
Day 2	19	0.53	0.94	1.13 (0.96,1.32)	1.33	1.75	16	0.51	0.77	1.00 (0.78,1.21)	1.23	1.37	35	0.51	0.87	1.03 (0.92,1.21)	1.31	1.75
Day 7	19	0.48	0.63	0.77 (0.67,0.90)	0.98	2.04	16	0.31	0.55	0.71 (0.56,1.12)	1.10	2.03	35	0.31	0.59	0.76 (0.65,0.90)	1.08	2.04
Day 14	19	0.54	0.70	0.73 (0.70,0.92)	0.95	1.57	16	0.28	0.54	0.69 (0.55,1.05)	1.08	1.92	35	0.28	0.63	0.73 (0.68,0.93)	1.04	1.92

Table 3: Descriptive summary statistics of percent monocyte cells (CD16-) of live PBMCs fold change from baseline by treatment.

	DVC-LVS						USAMARIID-LVS						Combined					
Study Visit	n	Min	Q1	Median (95% CI)	Q3	Max	n	Min	Q1	Median (95% CI)	Q3	Max	n	Min	Q1	Median (95% CI)	Q3	Max
Day 1	19	0.88	0.97	1.02 (0.98,1.11)	1.12	1.27	15	0.87	0.96	1.00 (0.97,1.07)	1.06	1.14	34	0.87	0.97	1.01 (0.97,1.04)	1.10	1.27
Day 2	19	0.73	0.92	0.97 (0.93,1.00)	1.00	1.19	16	0.82	0.98	1.00 (0.98,1.04)	1.03	1.29	35	0.73	0.95	0.99 (0.97,1.00)	1.02	1.29
Day 7	19	0.90	1.00	1.05 (1.00,1.12)	1.12	1.39	16	0.83	0.94	1.04 (0.95,1.14)	1.14	1.42	35	0.83	0.96	1.05 (1.01,1.09)	1.14	1.42
Day 14	19	0.78	1.03	1.08 (1.04,1.12)	1.12	1.43	16	0.87	1.03	1.09 (1.02,1.15)	1.15	1.44	35	0.78	1.03	1.09 (1.04,1.10)	1.13	1.44

Table 4: Descriptive summary statistics of percent T-cells of live PBMCs fold change from baseline by treatment.

	DVC-LVS						USAMARIID-LVS						Combined					
Study Visit	n	Min	Q1	Median (95% CI)	Q3	Max	n	Min	Q1	Median (95% CI)	Q3	Max	n	Min	Q1	Median (95% CI)	Q3	Max
Day 1	19	0.93	1.03	1.05 (1.03,1.13)	1.15	1.44	15	0.73	0.94	1.02 (0.98,1.11)	1.10	1.23	34	0.73	0.98	1.04 (1.02,1.09)	1.12	1.44
Day 2	19	0.83	0.94	0.99 (0.94,1.03)	1.04	1.43	16	0.79	0.99	1.01 (0.99,1.09)	1.09	1.39	35	0.79	0.97	1.00 (0.98,1.04)	1.06	1.43
Day 7	19	0.88	1.02	1.11 (1.03,1.26)	1.29	1.48	16	0.75	0.94	1.04 (0.94,1.18)	1.19	1.57	35	0.75	0.95	1.08 (1.00,1.14)	1.25	1.57
Day 14	19	0.71	1.03	1.08 (1.03,1.20)	1.21	1.53	16	0.83	1.00	1.08 (1.00,1.22)	1.22	1.45	35	0.71	1.02	1.08 (1.04,1.20)	1.22	1.53

Table 5: Descriptive summary statistics of percent T-cells (CD4+) of live PBMCs fold change from baseline by treatment.

	DVC-LVS						USAMARIID-LVS						Combined					
Study Visit	n	Min	Q1	Median (95% CI)	Q3	Max	n	Min	Q1	Median (95% CI)	Q3	Max	n	Min	Q1	Median (95% CI)	Q3	Max
Day 1	19	0.56	0.98	1.02 (0.99,1.09)	1.11	1.16	15	0.88	0.96	1.00 (0.96,1.06)	1.06	1.34	34	0.56	0.96	1.02 (0.97,1.06)	1.09	1.34

	DVC-LVS						USAMARIID-LVS						Combined					
Study Visit	n	Min	Q1	Median (95% CI)	Q3	Max	n	Min	Q1	Median (95% CI)	Q3	Max	n	Min	Q1	Median (95% CI)	Q3	Max
Day 2	19	0.62	0.87	0.93 (0.88,1.01)	1.01	1.16	16	0.82	0.92	0.96 (0.91,1.01)	1.01	1.15	35	0.62	0.88	0.94 (0.91,1.00)	1.01	1.16
Day 7	19	0.78	0.94	1.03 (0.94,1.12)	1.12	1.30	16	0.54	0.88	0.99 (0.88,1.15)	1.16	1.89	35	0.54	0.90	1.00 (0.94,1.06)	1.13	1.89
Day 14	19	0.85	0.96	1.07 (0.97,1.13)	1.13	1.36	16	0.94	1.00	1.06 (0.99,1.11)	1.12	1.45	35	0.85	0.97	1.06 (1.02,1.11)	1.13	1.45

Table 6: Descriptive summary statistics of percent T-cells (CD4-) of live PBMCs fold change from baseline by treatment.

	DVC-LVS						USAMARIID-LVS						Combined					
Study Visit	n	Min	Q1	Median (95% CI)	Q3	Max	n	Min	Q1	Median (95% CI)	Q3	Max	n	Min	Q1	Median (95% CI)	Q3	Max
Day 1	19	0.46	0.75	0.92 (0.77,1.02)	1.04	1.58	15	0.55	0.73	0.86 (0.74,0.96)	0.96	1.34	34	0.46	0.73	0.87 (0.77,0.97)	1.01	1.58
Day 2	19	0.55	0.75	0.86 (0.75,1.01)	1.09	1.42	16	0.57	0.85	0.96 (0.85,1.06)	1.06	1.42	35	0.55	0.79	0.89 (0.84,1.02)	1.07	1.42
Day 7	19	0.56	0.80	1.02 (0.81,1.30)	1.31	1.51	16	0.71	0.80	0.90 (0.79,1.02)	1.02	1.14	35	0.56	0.80	0.91 (0.82,1.06)	1.07	1.51
Day 14	19	0.39	0.79	0.92 (0.79,1.19)	1.23	1.88	16	0.49	0.75	0.94 (0.75,1.09)	1.12	1.61	35	0.39	0.77	0.93 (0.86,1.09)	1.19	1.88

Table 7: Descriptive summary statistics of percent natural killer cells (CD56 bright) of live PBMCs fold change from baseline by treatment.

	DVC-LVS						USAMARIID-LVS						Combined					
Study Visit	n	Min	Q1	Median (95% CI)	Q3	Max	n	Min	Q1	Median (95% CI)	Q3	Max	n	Min	Q1	Median (95% CI)	Q3	Max
Day 1	19	0.50	0.79	0.90 (0.83,1.08)	1.13	1.62	15	0.51	0.71	0.90 (0.73,1.08)	1.04	1.50	34	0.50	0.74	0.90 (0.83,1.00)	1.08	1.62
Day 2	19	0.44	0.76	0.83 (0.76,1.08)	1.13	1.92	16	0.44	0.90	1.05 (0.86,1.15)	1.16	1.57	35	0.44	0.76	0.95 (0.81,1.08)	1.16	1.92
Day 7	19	0.59	0.90	1.04 (0.91,1.27)	1.28	1.91	16	0.57	0.79	0.91 (0.81,1.15)	1.16	2.24	35	0.57	0.83	0.92 (0.89,1.11)	1.22	2.24
Day 14	19	0.22	0.74	1.00 (0.74,1.18)	1.25	2.53	16	0.41	0.78	1.10 (0.78,1.31)	1.34	1.88	35	0.22	0.75	1.04 (0.85,1.21)	1.32	2.53

Table 8: Descriptive summary statistics of percent natural killer cells (CD56 bright CD16+) of live PBMCs fold change from baseline by treatment.

	DVC-LVS						USAMARIID-LVS						Combined					
Study Visit	n	Min	Q1	Median (95% CI)	Q3	Max	n	Min	Q1	Median (95% CI)	Q3	Max	n	Min	Q1	Median (95% CI)	Q3	Max
Day 1	19	0.43	0.73	0.93 (0.75,1.02)	1.03	1.64	15	0.51	0.74	0.86 (0.75,0.93)	0.93	1.32	34	0.43	0.73	0.87 (0.77,0.96)	1.01	1.64
Day 2	19	0.58	0.76	0.83 (0.77,0.98)	1.03	1.39	16	0.58	0.86	0.97 (0.86,1.05)	1.05	1.41	35	0.58	0.77	0.92 (0.83,0.99)	1.06	1.41
Day 7	19	0.53	0.80	1.00 (0.80,1.25)	1.27	1.52	16	0.59	0.83	0.90 (0.83,1.00)	1.01	1.15	35	0.53	0.81	0.92 (0.83,1.03)	1.10	1.52
Day 14	19	0.41	0.81	0.92 (0.81,1.19)	1.23	1.66	16	0.52	0.78	0.94 (0.76,1.06)	1.09	1.65	35	0.41	0.80	0.92 (0.85,1.06)	1.18	1.66

Table 9: Descriptive summary statistics of percent natural killer cells (CD56 bright CD16-) of live PBMCs fold change from baseline by treatment.

	DVC-LVS						USAMARIID-LVS						Combined					
Study Visit	n	Min	Q1	Median (95% CI)	Q3	Max	n	Min	Q1	Median (95% CI)	Q3	Max	n	Min	Q1	Median (95% CI)	Q3	Max
Day 1	19	0.45	0.66	0.80 (0.67,1.05)	1.06	1.55	15	0.61	0.67	0.81 (0.63,1.11)	1.18	2.04	34	0.45	0.65	0.80 (0.72,1.04)	1.10	2.04
Day 2	19	0.44	0.73	0.87 (0.74,1.01)	1.07	1.50	16	0.57	0.71	0.91 (0.70,1.06)	1.07	2.17	35	0.44	0.71	0.87 (0.73,0.99)	1.07	2.17
Day 7	19	0.56	0.72	0.82 (0.76,1.05)	1.05	1.70	16	0.59	0.74	0.90 (0.77,1.13)	1.14	1.78	35	0.56	0.72	0.87 (0.78,1.01)	1.08	1.78
Day 14	19	0.38	0.78	0.90 (0.78,1.05)	1.05	2.32	16	0.41	0.70	0.90 (0.72,1.01)	1.05	2.40	35	0.38	0.75	0.90 (0.82,1.00)	1.05	2.40

Table 10: Descriptive summary statistics of percent natural killer cells (CD56 dim CD16-) of live PBMCs fold change from baseline by treatment.

	DVC-LVS						USAMARIID-LVS						Combined					
Study Visit	n	Min	Q1	Median (95% CI)	Q3	Max	n	Min	Q1	Median (95% CI)	Q3	Max	n	Min	Q1	Median (95% CI)	Q3	Max
Day 1	19	0.42	0.86	1.10 (0.92,1.20)	1.30	1.83	15	0.35	0.92	1.09 (0.97,1.21)	1.25	3.27	34	0.35	0.89	1.09 (0.97,1.16)	1.27	3.27

	DVC-LVS						USAMARIID-LVS						Combined					
Study Visit	n	Min	Q1	Median (95% CI)	Q3	Max	n	Min	Q1	Median (95% CI)	Q3	Max	n	Min	Q1	Median (95% CI)	Q3	Max
Day 2	19	0.61	0.82	1.02 (0.82,1.13)	1.14	2.55	16	0.31	0.80	1.10 (0.82,1.56)	1.56	2.61	35	0.31	0.82	1.05 (0.83,1.15)	1.43	2.61
Day 7	19	0.47	0.84	1.02 (0.84,1.29)	1.30	1.96	16	0.47	0.96	1.21 (1.00,1.41)	1.42	2.19	35	0.47	0.85	1.15 (0.95,1.29)	1.38	2.19
Day 14	19	0.29	0.74	0.86 (0.75,1.02)	1.15	2.46	16	0.40	0.73	1.01 (0.68,1.15)	1.17	1.68	35	0.29	0.74	0.93 (0.79,1.05)	1.19	2.46

Table 11: Descriptive summary statistics of percent B-cells of live PBMCs fold change from baseline by treatment.

	DVC-LVS						USAMARIID-LVS						Combined					
Study Visit	n	Min	Q1	Median (95% CI)	Q3	Max	n	Min	Q1	Median (95% CI)	Q3	Max	n	Min	Q1	Median (95% CI)	Q3	Max
Day 1	19	0.06	0.76	0.94 (0.79,1.01)	1.10	1.93	15	0.64	0.97	1.29 (1.05,1.62)	1.76	14.27	34	0.06	0.85	0.99 (0.89,1.28)	1.46	14.27
Day 2	19	0.45	0.75	0.83 (0.77,1.34)	1.36	2.63	16	0.62	0.73	1.12 (0.73,1.27)	1.30	2.00	35	0.45	0.73	0.94 (0.80,1.22)	1.36	2.63
Day 7	19	0.17	0.72	0.87 (0.75,1.24)	1.29	2.57	16	0.48	0.65	0.99 (0.67,1.27)	1.31	5.83	35	0.17	0.68	0.88 (0.78,1.18)	1.31	5.83
Day 14	19	0.55	0.81	0.88 (0.82,1.39)	1.46	2.80	16	0.61	0.93	1.02 (0.95,1.28)	1.28	2.26	35	0.55	0.83	1.00 (0.85,1.19)	1.32	2.80

Table 12: Descriptive summary statistics of percent T-cells (CD4+ CCR5 high) of live PBMCs fold change from baseline by treatment.

	DVC-LVS						USAMARIID-LVS						Combined					
Study Visit	n	Min	Q1	Median (95% CI)	Q3	Max	n	Min	Q1	Median (95% CI)	Q3	Max	n	Min	Q1	Median (95% CI)	Q3	Max
Day 1	19	0.09	0.78	0.91 (0.78,1.13)	1.13	1.93	15	0.61	0.91	1.27 (0.93,1.70)	1.62	5.97	34	0.09	0.78	1.00 (0.88,1.21)	1.45	5.97
Day 2	19	0.33	0.65	0.84 (0.68,1.15)	1.17	2.51	16	0.26	0.72	0.89 (0.67,1.26)	1.29	1.96	35	0.26	0.65	0.87 (0.79,1.03)	1.23	2.51
Day 7	19	0.43	0.62	0.89 (0.63,1.36)	1.41	3.25	16	0.31	0.63	0.88 (0.63,1.07)	1.10	1.96	35	0.31	0.62	0.89 (0.68,1.07)	1.34	3.25
Day 14	19	0.50	0.84	1.08 (0.85,1.24)	1.30	4.28	16	0.51	0.89	1.00 (0.90,1.12)	1.12	2.55	35	0.50	0.85	1.07 (0.88,1.09)	1.23	4.28

Table 13: Descriptive summary statistics of percent T-cells (CD4- CCR5 high) of live PBMCs fold change from baseline by treatment.

	DVC-LVS						USAMARIID-LVS						Combined					
Study Visit	n	Min	Q1	Median (95% CI)	Q3	Max	n	Min	Q1	Median (95% CI)	Q3	Max	n	Min	Q1	Median (95% CI)	Q3	Max
Day 1	19	0.14	0.68	0.93 (0.70,1.19)	1.21	1.94	15	0.39	0.56	1.08 (0.59,1.50)	1.47	1.79	34	0.14	0.65	1.00 (0.70,1.17)	1.25	1.94
Day 2	19	0.37	0.73	0.82 (0.75,0.97)	1.05	1.37	16	0.27	0.55	0.80 (0.55,1.14)	1.16	1.70	35	0.27	0.65	0.81 (0.75,0.97)	1.13	1.70
Day 7	19	0.22	0.66	0.83 (0.68,0.94)	1.07	2.23	16	0.21	0.51	0.88 (0.51,1.21)	1.19	2.65	35	0.21	0.63	0.83 (0.65,1.04)	1.19	2.65
Day 14	19	0.37	0.81	1.09 (0.85,1.37)	1.37	2.14	16	0.37	0.62	0.77 (0.62,1.11)	1.14	2.16	35	0.37	0.73	0.88 (0.77,1.24)	1.37	2.16

Table 14: Descriptive summary statistics of percent B-cells (CCR5 high) of live PBMCs fold change from baseline by treatment.

	DVC-LVS						USAMARIID-LVS						Combined					
Study Visit	n	Min	Q1	Median (95% CI)	Q3	Max	n	Min	Q1	Median (95% CI)	Q3	Max	n	Min	Q1	Median (95% CI)	Q3	Max
Day 1	19	0.11	0.71	0.97 (0.71,1.15)	1.29	2.43	15	0.70	0.97	1.19 (0.95,1.36)	1.38	4.85	34	0.11	0.85	1.05 (0.94,1.25)	1.39	4.85
Day 2	19	0.56	0.90	1.06 (0.94,1.30)	1.48	2.86	16	0.58	0.92	1.06 (0.93,1.27)	1.26	1.78	35	0.56	0.92	1.06 (0.95,1.21)	1.35	2.86
Day 7	19	0.67	0.82	1.11 (0.85,1.66)	1.70	2.90	16	0.50	0.75	0.89 (0.76,0.96)	0.97	1.26	35	0.50	0.77	0.94 (0.85,1.12)	1.23	2.90
Day 14	19	0.52	0.77	0.90 (0.78,1.35)	1.44	2.81	16	0.48	0.80	0.96 (0.81,1.06)	1.07	2.26	35	0.48	0.78	0.93 (0.81,1.03)	1.24	2.81

Table 15: Descriptive summary statistics of percent T-cells (CD4+ CD69 high) of live PBMCs fold change from baseline by treatment.

	DVC-LVS						USAMARIID-LVS						Combined					
Study Visit	n	Min	Q1	Median (95% CI)	Q3	Max	n	Min	Q1	Median (95% CI)	Q3	Max	n	Min	Q1	Median (95% CI)	Q3	Max
Day 1	19	0.26	0.76	1.00 (0.76,1.14)	1.15	1.40	15	0.72	0.90	1.00 (0.88,1.17)	1.18	2.63	34	0.26	0.83	1.00 (0.89,1.13)	1.17	2.63

	DVC-LVS						USAMARIID-LVS						Combined					
Study Visit	n	Min	Q1	Median (95% CI)	Q3	Max	n	Min	Q1	Median (95% CI)	Q3	Max	n	Min	Q1	Median (95% CI)	Q3	Max
Day 2	19	0.74	0.88	1.07 (0.89,1.26)	1.29	2.77	16	0.64	0.90	1.04 (0.93,1.22)	1.24	1.45	35	0.64	0.88	1.04 (0.93,1.22)	1.28	2.77
Day 7	19	0.66	0.82	1.09 (0.84,1.33)	1.36	2.84	16	0.24	0.76	0.89 (0.77,1.02)	1.02	2.54	35	0.24	0.79	0.94 (0.84,1.13)	1.30	2.84
Day 14	19	0.58	0.82	0.92 (0.83,1.06)	1.12	1.79	16	0.53	0.89	0.98 (0.87,1.13)	1.14	2.60	35	0.53	0.82	0.94 (0.84,1.06)	1.14	2.60

Table 16: Descriptive summary statistics of percent T-cells (CD4+-CD69 high) of live PBMCs fold change from baseline by treatment.

	DVC-LVS						USAMARIID-LVS						Combined					
Study Visit	n	Min	Q1	Median (95% CI)	Q3	Max	n	Min	Q1	Median (95% CI)	Q3	Max	n	Min	Q1	Median (95% CI)	Q3	Max
Day 1	19	0.43	0.74	0.95 (0.76,1.20)	1.25	1.96	15	0.50	0.78	0.92 (0.79,1.65)	1.56	1350	34	0.43	0.76	0.93 (0.81,1.18)	1.32	1350
Day 2	19	0.15	0.79	1.07 (0.83,1.37)	1.37	2.14	16	0.20	0.79	0.88 (0.79,1.15)	1.24	720	35	0.15	0.78	0.98 (0.83,1.12)	1.37	720
Day 7	19	0.33	0.78	0.95 (0.81,1.41)	1.50	2.22	16	0.28	0.79	1.00 (0.80,1.17)	1.17	1290	35	0.28	0.78	0.96 (0.91,1.14)	1.30	1290
Day 14	19	0.46	0.67	1.08 (0.71,1.41)	1.43	2.12	16	0.34	0.83	0.96 (0.79,1.18)	1.18	810	35	0.34	0.68	1.00 (0.88,1.14)	1.36	810

Table 17: Descriptive summary statistics of percent natural killer cells (CD56 bright CD69 high) of live PBMCs fold change from baseline by treatment.

	DVC-LVS						USAMARIID-LVS						Combined					
Study Visit	n	Min	Q1	Median (95% CI)	Q3	Max	n	Min	Q1	Median (95% CI)	Q3	Max	n	Min	Q1	Median (95% CI)	Q3	Max
Day 1	19	0.67	0.96	1.21 (0.96,1.51)	1.54	2.01	15	0.65	1.04	1.14 (1.01,1.32)	1.29	1.50	34	0.65	0.98	1.17 (1.01,1.30)	1.40	2.01
Day 2	19	0.65	1.06	1.31 (1.10,1.56)	1.59	3.88	16	0.88	1.07	1.37 (1.06,1.63)	1.59	2.58	35	0.65	1.06	1.31 (1.11,1.49)	1.59	3.88
Day 7	19	0.43	0.92	1.08 (0.93,1.57)	1.59	6.05	16	0.24	0.86	1.02 (0.88,1.14)	1.14	7.21	35	0.24	0.91	1.05 (0.93,1.15)	1.31	7.21
Day 14	19	0.49	0.97	1.09 (0.98,1.25)	1.27	2.12	16	0.51	0.97	1.08 (0.97,1.26)	1.19	3.77	35	0.49	0.97	1.09 (0.98,1.14)	1.27	3.77

Table 18: Descriptive summary statistics of percent natural killer cells (CD56 dim CD16- CD69 high) of live PBMCs fold change from baseline by treatment.

	DVC-LVS						USAMARIID-LVS						Combined					
Study Visit	n	Min	Q1	Median (95% CI)	Q3	Max	n	Min	Q1	Median (95% CI)	Q3	Max	n	Min	Q1	Median (95% CI)	Q3	Max
Day 1	19	0.24	0.66	0.85 (0.66,0.98)	1.04	1.53	15	0.47	0.75	0.90 (0.77,1.01)	1.01	1.32	34	0.24	0.67	0.86 (0.77,0.97)	1.01	1.53
Day 2	19	0.32	0.64	0.78 (0.64,0.84)	0.86	1.76	16	0.58	0.77	0.95 (0.78,1.14)	1.14	1.98	35	0.32	0.73	0.81 (0.76,0.97)	1.00	1.98
Day 7	19	0.31	0.62	0.86 (0.69,1.01)	1.03	2.59	16	0.59	0.75	0.87 (0.75,1.01)	1.02	1.84	35	0.31	0.68	0.86 (0.80,1.01)	1.03	2.59
Day 14	19	0.33	0.68	0.81 (0.73,1.01)	1.06	1.87	16	0.51	0.81	1.00 (0.79,1.18)	1.21	1.45	35	0.33	0.70	0.90 (0.79,1.04)	1.12	1.87

Table 19: Descriptive summary statistics of percent B-cells (CD69 high) of live PBMCs fold change from baseline by treatment.

	DVC-LVS						USAMARIID-LVS						Combined					
Study Visit	n	Min	Q1	Median (95% CI)	Q3	Max	n	Min	Q1	Median (95% CI)	Q3	Max	n	Min	Q1	Median (95% CI)	Q3	Max
Day 1	19	0.79	0.99	1.17 (0.99,1.34)	1.34	2.36	15	0.93	1.00	1.11 (0.99,1.15)	1.21	1.78	34	0.79	0.99	1.12 (1.02,1.26)	1.32	2.36
Day 2	19	0.71	0.94	1.13 (0.97,1.31)	1.32	1.62	16	0.75	1.02	1.15 (1.03,1.22)	1.22	1.40	35	0.71	0.98	1.14 (1.03,1.22)	1.29	1.62
Day 7	19	0.78	0.94	1.22 (0.96,1.40)	1.43	1.99	16	0.70	0.88	1.00 (0.89,1.24)	1.25	1.57	35	0.70	0.91	1.11 (0.96,1.24)	1.32	1.99
Day 14	19	0.91	1.07	1.17 (1.08,1.25)	1.27	3.04	16	0.57	1.00	1.06 (1.00,1.50)	1.56	1.85	35	0.57	1.04	1.10 (1.06,1.25)	1.41	3.04

Table 20: Descriptive summary statistics of percent T-cells (CD4+ HLA-DR+) of live PBMCs fold change from baseline by treatment.

	DVC-LVS						USAMARIID-LVS						Combined					
Study Visit	n	Min	Q1	Median (95% CI)	Q3	Max	n	Min	Q1	Median (95% CI)	Q3	Max	n	Min	Q1	Median (95% CI)	Q3	Max

	DVC-LVS						USAMARIID-LVS						Combined					
Study Visit	n	Min	Q1	Median (95% CI)	Q3	Max	n	Min	Q1	Median (95% CI)	Q3	Max	n	Min	Q1	Median (95% CI)	Q3	Max
Day 1	19	0.55	1.04	1.12 (1.04,1.29)	1.31	1.85	15	0.77	1.04	1.11 (1.03,1.20)	1.21	1.46	34	0.55	1.04	1.11 (1.06,1.20)	1.28	1.85
Day 2	19	0.47	0.90	1.15 (0.90,1.29)	1.29	1.63	16	0.74	0.97	1.05 (1.00,1.28)	1.29	2.00	35	0.47	0.90	1.10 (1.01,1.25)	1.29	2.00
Day 7	19	0.81	1.07	1.48 (1.11,1.85)	1.86	4.58	16	0.63	0.84	1.20 (0.89,1.74)	1.81	3.50	35	0.63	0.98	1.41 (1.08,1.74)	1.86	4.58
Day 14	19	0.52	1.09	1.65 (1.10,1.98)	2.24	12.09	16	0.53	0.98	1.68 (1.01,2.25)	2.26	5.17	35	0.52	1.07	1.65 (1.22,1.98)	2.27	12.09

Table 21: Descriptive summary statistics of percent T-cells (CD4- HLA-DR+) of live PBMCs fold change from baseline by treatment.

	DVC-LVS						USAMARIID-LVS						Combined					
Study Visit	n	Min	Q1	Median (95% CI)	Q3	Max	n	Min	Q1	Median (95% CI)	Q3	Max	n	Min	Q1	Median (95% CI)	Q3	Max
Day 1	19	0.26	0.73	0.89 (0.73,1.06)	1.06	1.39	15	0.77	0.85	0.92 (0.85,1.17)	1.12	1.77	34	0.26	0.83	0.90 (0.85,1.05)	1.07	1.77
Day 2	19	0.64	0.90	1.10 (0.97,1.15)	1.16	1.52	16	0.41	0.93	1.09 (0.93,1.22)	1.23	1.41	35	0.41	0.92	1.10 (0.98,1.15)	1.20	1.52
Day 7	19	0.02	0.85	0.99 (0.85,1.13)	1.15	1.32	16	0.00	0.78	0.94 (0.78,1.19)	1.19	1.74	35	0.00	0.80	0.96 (0.84,1.13)	1.18	1.74
Day 14	19	0.59	0.87	0.96 (0.90,1.08)	1.08	2.89	16	0.12	0.90	0.95 (0.90,1.07)	1.07	1.31	35	0.12	0.90	0.96 (0.90,1.05)	1.08	2.89

Table 22: Descriptive summary statistics of monocyte cells (CD86-) mean fluorescence intensity fold change from baseline by treatment.

	DVC-LVS						USAMARIID-LVS						Combined					
Study Visit	n	Min	Q1	Median (95% CI)	Q3	Max	n	Min	Q1	Median (95% CI)	Q3	Max	n	Min	Q1	Median (95% CI)	Q3	Max
Day 1	19	0.47	0.89	0.93 (0.90,1.00)	1.01	1.12	15	0.76	0.92	1.00 (0.91,1.12)	1.12	1.90	34	0.47	0.90	0.96 (0.92,1.02)	1.07	1.90
Day 2	19	0.69	0.80	0.92 (0.80,1.00)	1.00	1.15	16	0.64	0.95	1.06 (0.96,1.10)	1.11	1.26	35	0.64	0.84	1.00 (0.90,1.06)	1.07	1.26
Day 7	19	0.36	0.80	0.91 (0.82,1.01)	1.01	1.19	16	0.50	0.84	1.01 (0.86,1.13)	1.13	1.65	35	0.36	0.81	0.92 (0.86,1.01)	1.08	1.65
Day 14	19	0.68	0.88	0.96 (0.90,1.04)	1.04	1.31	16	0.70	0.85	1.01 (0.85,1.09)	1.10	1.41	35	0.68	0.85	1.00 (0.90,1.05)	1.08	1.41

Table 23: Descriptive summary statistics of monocyte cells (CD16+ CD86-) mean fluorescence intensity fold change from baseline by treatment.

	DVC-LVS						USAMARIID-LVS						Combined					
Study Visit	n	Min	Q1	Median (95% CI)	Q3	Max	n	Min	Q1	Median (95% CI)	Q3	Max	n	Min	Q1	Median (95% CI)	Q3	Max
Day 1	19	0.01	0.75	0.88 (0.78,1.05)	1.11	1.41	15	0.71	0.79	0.83 (0.79,1.14)	1.08	1.78	34	0.01	0.78	0.85 (0.80,0.99)	1.12	1.78
Day 2	19	0.62	1.00	1.11 (1.00,1.24)	1.24	1.47	16	0.03	0.92	1.01 (0.95,1.22)	1.22	1.38	35	0.03	0.96	1.08 (1.00,1.19)	1.24	1.47
Day 7	19	0.54	0.88	1.00 (0.90,1.05)	1.07	1.84	16	0.03	0.72	0.92 (0.72,1.09)	1.09	1.58	35	0.03	0.81	0.98 (0.89,1.05)	1.09	1.84
Day 14	19	0.22	0.81	0.98 (0.86,1.05)	1.06	79.70	16	0.01	0.82	0.94 (0.86,0.98)	0.98	1.28	35	0.01	0.81	0.94 (0.86,1.00)	1.05	79.70

Table 24: Descriptive summary statistics of monocyte cells (CD16- CD86-) mean fluorescence intensity fold change from baseline by treatment.

	DVC-LVS						USAMARIID-LVS						Combined					
Study Visit	n	Min	Q1	Median (95% CI)	Q3	Max	n	Min	Q1	Median (95% CI)	Q3	Max	n	Min	Q1	Median (95% CI)	Q3	Max
Day 1	19	0.76	0.90	0.95 (0.90,1.02)	1.02	1.24	15	0.62	0.81	0.92 (0.81,1.02)	1.01	1.12	34	0.62	0.85	0.93 (0.88,0.99)	1.02	1.24
Day 2	19	0.68	0.88	0.95 (0.89,1.10)	1.11	1.55	16	0.74	0.85	0.93 (0.85,1.00)	1.00	1.22	35	0.68	0.86	0.95 (0.89,1.01)	1.04	1.55
Day 7	19	0.67	0.79	0.85 (0.79,0.93)	0.94	1.53	16	0.66	0.80	0.88 (0.81,0.93)	0.93	1.07	35	0.66	0.79	0.86 (0.84,0.91)	0.94	1.53
Day 14	19	0.70	0.81	0.89 (0.84,1.04)	1.06	1.18	16	0.73	0.79	0.82 (0.79,0.88)	0.87	1.22	35	0.70	0.79	0.85 (0.82,0.90)	0.95	1.22

Table 25: Descriptive summary statistics of monocyte cells (CCR5-) mean fluorescence intensity fold change from baseline by treatment.

	DVC-LVS						USAMARIID-LVS						Combined					
Study Visit	n	Min	Q1	Median (95% CI)	Q3	Max	n	Min	Q1	Median (95% CI)	Q3	Max	n	Min	Q1	Median (95% CI)	Q3	Max
Day 1	19	0.91	0.96	1.02 (0.96,1.06)	1.06	1.18	15	0.76	0.90	0.97 (0.89,1.09)	1.09	1.20	34	0.76	0.94	1.00 (0.96,1.04)	1.08	1.20
Day 2	19	0.92	0.98	1.03 (1.00,1.11)	1.12	1.52	16	0.78	0.94	0.99 (0.94,1.10)	1.10	1.33	35	0.78	0.96	1.01 (0.97,1.09)	1.11	1.52
Day 7	19	0.88	0.92	0.98 (0.93,1.03)	1.04	1.36	16	0.74	0.89	0.94 (0.88,1.02)	1.03	1.27	35	0.74	0.90	0.96 (0.91,1.01)	1.04	1.36
Day 14	19	0.84	0.93	0.99 (0.94,1.04)	1.07	1.28	16	0.87	0.91	0.96 (0.91,1.01)	1.00	1.22	35	0.84	0.92	0.98 (0.93,1.01)	1.04	1.28

Table 26: Descriptive summary statistics of monocyte cells (CD16+ CCR5-) mean fluorescence intensity fold change from baseline by treatment.

	DVC-LVS						USAMARIID-LVS						Combined					
Study Visit	n	Min	Q1	Median (95% CI)	Q3	Max	n	Min	Q1	Median (95% CI)	Q3	Max	n	Min	Q1	Median (95% CI)	Q3	Max
Day 1	19	0.74	0.88	0.94 (0.88,1.03)	1.04	1.27	15	0.62	0.79	0.91 (0.80,1.01)	1.01	1.11	34	0.62	0.84	0.93 (0.88,1.00)	1.03	1.27
Day 2	19	0.66	0.87	0.96 (0.87,1.13)	1.15	1.57	16	0.76	0.84	0.93 (0.84,1.01)	1.01	1.21	35	0.66	0.86	0.94 (0.89,1.03)	1.05	1.57
Day 7	19	0.66	0.76	0.85 (0.76,0.92)	0.93	1.55	16	0.65	0.82	0.90 (0.82,0.94)	0.94	1.09	35	0.65	0.78	0.89 (0.82,0.91)	0.94	1.55
Day 14	19	0.68	0.79	0.91 (0.82,1.05)	1.05	1.17	16	0.72	0.80	0.83 (0.81,0.90)	0.90	1.24	35	0.68	0.79	0.85 (0.82,0.92)	0.96	1.24

Table 27: Descriptive summary statistics of monocyte cells (CD16- CCR5-) mean fluorescence intensity fold change from baseline by treatment.

	DVC-LVS						USAMARIID-LVS						Combined					
Study Visit	n	Min	Q1	Median (95% CI)	Q3	Max	n	Min	Q1	Median (95% CI)	Q3	Max	n	Min	Q1	Median (95% CI)	Q3	Max
Day 1	19	0.66	0.91	0.94 (0.91,0.97)	0.97	1.14	15	0.84	0.90	0.99 (0.92,1.05)	1.05	1.38	34	0.66	0.90	0.94 (0.92,0.99)	1.02	1.38
Day 2	19	0.80	0.90	0.93 (0.90,1.00)	1.04	1.09	16	0.80	0.91	0.93 (0.91,1.01)	1.01	1.13	35	0.80	0.90	0.93 (0.91,0.98)	1.01	1.13
Day 7	19	0.78	0.87	0.93 (0.87,1.02)	1.03	1.14	16	0.78	0.90	0.94 (0.89,1.00)	1.00	1.31	35	0.78	0.87	0.94 (0.88,1.00)	1.02	1.31
Day 14	19	0.81	0.91	0.99 (0.91,1.04)	1.06	1.20	16	0.83	0.89	0.96 (0.89,1.00)	1.01	1.18	35	0.81	0.91	0.97 (0.94,1.01)	1.02	1.20

Table 28: Descriptive summary statistics of T-cells (CCR5-) mean fluorescence intensity fold change from baseline by treatment.

	DVC-LVS						USAMARIID-LVS						Combined					
Study Visit	n	Min	Q1	Median (95% CI)	Q3	Max	n	Min	Q1	Median (95% CI)	Q3	Max	n	Min	Q1	Median (95% CI)	Q3	Max
Day 1	19	0.78	0.92	0.95 (0.93,0.97)	0.98	1.13	15	0.80	0.89	1.01 (0.89,1.04)	1.06	1.28	34	0.78	0.91	0.95 (0.93,1.01)	1.04	1.28
Day 2	19	0.86	0.93	0.95 (0.93,1.01)	1.03	1.12	16	0.84	0.92	0.95 (0.91,1.03)	1.03	1.10	35	0.84	0.93	0.95 (0.94,0.98)	1.03	1.12
Day 7	19	0.62	0.90	0.95 (0.91,1.02)	1.03	1.07	16	0.77	0.90	0.93 (0.92,1.04)	1.04	1.33	35	0.62	0.90	0.95 (0.92,1.00)	1.04	1.33
Day 14	19	0.86	0.94	0.98 (0.94,1.02)	1.04	1.19	16	0.82	0.92	0.96 (0.93,0.99)	0.99	1.11	35	0.82	0.93	0.97 (0.94,0.99)	1.01	1.19

Table 29: Descriptive summary statistics of T-cells (CD4+ CCR5-) mean fluorescence intensity fold change from baseline by treatment.

	DVC-LVS						USAMARIID-LVS						Combined					
Study Visit	n	Min	Q1	Median (95% CI)	Q3	Max	n	Min	Q1	Median (95% CI)	Q3	Max	n	Min	Q1	Median (95% CI)	Q3	Max
Day 1	19	0.68	0.89	0.94 (0.89,0.98)	0.99	1.14	15	0.82	0.89	0.96 (0.90,1.08)	1.06	1.29	34	0.68	0.89	0.94 (0.92,0.99)	1.03	1.29
Day 2	19	0.78	0.88	0.95 (0.88,0.99)	1.02	1.14	16	0.76	0.89	0.95 (0.89,1.00)	0.99	1.18	35	0.76	0.88	0.95 (0.90,0.98)	1.01	1.18
Day 7	19	0.79	0.87	0.93 (0.89,1.06)	1.06	1.22	16	0.73	0.84	0.93 (0.84,0.96)	0.97	1.50	35	0.73	0.85	0.93 (0.89,0.97)	1.02	1.50
Day 14	19	0.84	0.92	0.98 (0.92,1.08)	1.09	1.40	16	0.80	0.91	0.96 (0.91,1.02)	1.02	1.32	35	0.80	0.91	0.96 (0.93,1.00)	1.05	1.40

Table 30: Descriptive summary statistics of T-cells (CD4- CCR5-) mean fluorescence intensity fold change from baseline by treatment.

	DVC-LVS						USAMARIID-LVS						Combined					
Study Visit	n	Min	Q1	Median (95% CI)	Q3	Max	n	Min	Q1	Median (95% CI)	Q3	Max	n	Min	Q1	Median (95% CI)	Q3	Max
Day 1	19	0.75	0.94	0.96 (0.95,0.99)	1.00	1.22	15	0.74	0.87	0.96 (0.86,1.05)	1.06	1.30	34	0.74	0.89	0.96 (0.95,0.99)	1.01	1.30

	DVC-LVS						USAMARIID-LVS						Combined					
Study Visit	n	Min	Q1	Median (95% CI)	Q3	Max	n	Min	Q1	Median (95% CI)	Q3	Max	n	Min	Q1	Median (95% CI)	Q3	Max
Day 2	19	0.74	0.94	0.97 (0.94,1.01)	1.02	1.22	16	0.66	0.89	0.97 (0.88,1.02)	1.03	1.17	35	0.66	0.92	0.97 (0.94,1.00)	1.02	1.22
Day 7	19	0.81	0.90	0.92 (0.90,1.00)	1.01	1.26	16	0.68	0.85	0.94 (0.85,1.03)	1.03	1.19	35	0.68	0.89	0.92 (0.90,1.00)	1.02	1.26
Day 14	19	0.69	0.94	0.98 (0.96,1.07)	1.10	1.29	16	0.70	0.89	0.94 (0.90,0.99)	0.99	1.08	35	0.69	0.91	0.96 (0.93,1.00)	1.05	1.29

Table 31: Descriptive summary statistics of natural killer cells (CD56 bright CCR5-) mean fluorescence intensity fold change from baseline by treatment.

	DVC-LVS						USAMARIID-LVS						Combined					
Study Visit	n	Min	Q1	Median (95% CI)	Q3	Max	n	Min	Q1	Median (95% CI)	Q3	Max	n	Min	Q1	Median (95% CI)	Q3	Max
Day 1	19	0.78	0.92	1.01 (0.92,1.04)	1.04	1.17	15	0.73	0.81	0.94 (0.79,1.07)	1.07	1.16	34	0.73	0.89	0.97 (0.92,1.03)	1.06	1.17
Day 2	19	0.75	0.95	1.01 (0.95,1.06)	1.09	1.25	16	0.70	0.85	0.94 (0.85,1.06)	1.06	1.36	35	0.70	0.88	1.00 (0.92,1.04)	1.06	1.36
Day 7	19	0.61	0.91	0.98 (0.93,1.05)	1.07	1.14	16	0.68	0.82	0.97 (0.83,1.06)	1.07	1.25	35	0.61	0.88	0.98 (0.90,1.04)	1.07	1.25
Day 14	19	0.77	0.91	0.99 (0.92,1.09)	1.09	1.42	16	0.72	0.82	0.86 (0.83,0.95)	0.95	1.19	35	0.72	0.86	0.95 (0.88,0.97)	1.01	1.42

Table 32: Descriptive summary statistics of natural killer cells (CD56 bright CD16+ CCR5-) mean fluorescence intensity fold change from baseline by treatment.

	DVC-LVS						USAMARIID-LVS						Combined					
Study Visit	n	Min	Q1	Median (95% CI)	Q3	Max	n	Min	Q1	Median (95% CI)	Q3	Max	n	Min	Q1	Median (95% CI)	Q3	Max
Day 1	19	0.72	0.94	0.96 (0.94,1.01)	1.01	1.23	15	0.74	0.89	0.97 (0.86,1.06)	1.06	1.36	34	0.72	0.93	0.97 (0.94,1.01)	1.05	1.36
Day 2	19	0.72	0.93	0.96 (0.93,1.03)	1.03	1.25	16	0.62	0.91	0.96 (0.90,1.04)	1.05	1.19	35	0.62	0.93	0.96 (0.93,1.03)	1.04	1.25
Day 7	19	0.79	0.90	0.94 (0.92,1.00)	1.00	1.31	16	0.68	0.84	0.92 (0.84,1.05)	1.06	1.25	35	0.68	0.87	0.94 (0.89,0.98)	1.02	1.31
Day 14	19	0.66	0.93	1.00 (0.93,1.11)	1.12	1.26	16	0.66	0.90	0.95 (0.91,0.98)	0.99	1.19	35	0.66	0.92	0.97 (0.93,1.00)	1.05	1.26

Table 33: Descriptive summary statistics of natural killer cells (CD56 bright CD16- CCR5-) mean fluorescence intensity fold change from baseline by treatment.

	DVC-LVS						USAMARIID-LVS						Combined					
Study Visit	n	Min	Q1	Median (95% CI)	Q3	Max	n	Min	Q1	Median (95% CI)	Q3	Max	n	Min	Q1	Median (95% CI)	Q3	Max
Day 1	19	0.73	0.87	0.96 (0.87,1.00)	1.01	1.78	15	0.77	0.86	0.92 (0.86,1.06)	1.03	1.27	34	0.73	0.87	0.95 (0.87,0.98)	1.02	1.78
Day 2	19	0.73	0.89	1.01 (0.94,1.16)	1.23	2.29	16	0.68	0.86	0.96 (0.86,1.04)	1.05	1.35	35	0.68	0.86	0.98 (0.95,1.06)	1.14	2.29
Day 7	19	0.84	0.89	0.96 (0.89,1.02)	1.07	3.32	16	0.48	0.80	0.96 (0.83,1.07)	1.07	1.49	35	0.48	0.86	0.96 (0.89,1.03)	1.08	3.32
Day 14	19	0.69	0.84	0.91 (0.85,0.99)	1.03	2.14	16	0.65	0.86	0.95 (0.86,1.07)	1.08	1.48	35	0.65	0.84	0.91 (0.86,1.02)	1.07	2.14

Table 34: Descriptive summary statistics of T-cells (CD4- CD69-) mean fluorescence intensity fold change from baseline by treatment.

	DVC-LVS						USAMARIID-LVS						Combined					
Study Visit	n	Min	Q1	Median (95% CI)	Q3	Max	n	Min	Q1	Median (95% CI)	Q3	Max	n	Min	Q1	Median (95% CI)	Q3	Max
Day 1	19	0.67	0.78	0.87 (0.78,0.98)	0.99	1.17	15	0.23	0.76	0.87 (0.74,1.00)	1.02	1.36	34	0.23	0.78	0.87 (0.81,0.97)	1.00	1.36
Day 2	19	0.73	0.82	0.91 (0.84,1.08)	1.09	1.50	16	0.59	0.84	0.95 (0.83,1.03)	1.03	1.56	35	0.59	0.82	0.93 (0.87,1.02)	1.05	1.56
Day 7	19	0.67	0.84	0.91 (0.85,0.99)	1.02	1.72	16	0.29	0.72	0.90 (0.72,1.06)	1.06	1.99	35	0.29	0.81	0.91 (0.85,0.99)	1.05	1.99
Day 14	19	0.57	0.84	0.99 (0.84,1.19)	1.20	1.54	16	0.08	0.86	1.00 (0.90,1.03)	1.03	1.89	35	0.08	0.84	0.99 (0.94,1.04)	1.17	1.89

Table 35: Descriptive summary statistics of natural killer cells (CD56 bright CD69-) mean fluorescence intensity fold change from baseline by treatment.

	DVC-LVS						USAMARIID-LVS						Combined					
Study Visit	n	Min	Q1	Median (95% CI)	Q3	Max	n	Min	Q1	Median (95% CI)	Q3	Max	n	Min	Q1	Median (95% CI)	Q3	Max
Day 1	19	0.33	0.63	0.84 (0.65,0.94)	0.96	2.25	15	0.21	0.72	0.95 (0.79,1.12)	1.12	1.99	34	0.21	0.65	0.89 (0.75,0.96)	1.08	2.25
Day 2	19	0.37	0.85	1.09 (0.85,1.26)	1.26	3.25	16	0.30	0.82	1.21 (0.83,1.46)	1.46	1.81	35	0.30	0.85	1.10 (0.94,1.27)	1.45	3.25
Day 7	19	0.35	0.80	0.99 (0.83,1.21)	1.23	3.44	16	0.06	0.64	1.01 (0.65,1.33)	1.34	3.62	35	0.06	0.73	0.99 (0.78,1.16)	1.29	3.62
Day 14	19	0.18	0.83	1.08 (0.84,1.21)	1.25	2.76	16	0.21	0.85	1.02 (0.89,1.29)	1.30	1.65	35	0.18	0.83	1.04 (0.91,1.21)	1.29	2.76

Table 36: Descriptive summary statistics of natural killer cells (CD56 bright CD16+ CD69-) mean fluorescence intensity fold change from baseline by treatment.

	DVC-LVS						USAMARIID-LVS						Combined					
Study Visit	n	Min	Q1	Median (95% CI)	Q3	Max	n	Min	Q1	Median (95% CI)	Q3	Max	n	Min	Q1	Median (95% CI)	Q3	Max
Day 1	19	0.74	0.80	0.88 (0.80,1.00)	1.01	1.12	15	0.54	0.82	0.85 (0.82,0.98)	1.00	1.40	34	0.54	0.81	0.87 (0.83,0.99)	1.01	1.40
Day 2	19	0.56	0.81	0.89 (0.84,1.04)	1.05	1.48	16	0.59	0.84	0.90 (0.84,0.95)	0.95	1.62	35	0.56	0.83	0.90 (0.86,0.96)	1.02	1.62
Day 7	19	0.64	0.84	0.88 (0.85,0.98)	1.01	1.59	16	0.39	0.70	0.88 (0.70,1.08)	1.09	2.23	35	0.39	0.79	0.88 (0.82,0.97)	1.06	2.23
Day 14	19	0.53	0.82	0.98 (0.87,1.17)	1.19	1.43	16	0.04	0.78	0.96 (0.80,1.07)	1.07	2.02	35	0.04	0.79	0.97 (0.87,1.07)	1.17	2.02

Table 37: Descriptive summary statistics of natural killer cells (CD56 bright CD16- CD69-) mean fluorescence intensity fold change from baseline by treatment.

	DVC-LVS						USAMARIID-LVS						Combined					
Study Visit	n	Min	Q1	Median (95% CI)	Q3	Max	n	Min	Q1	Median (95% CI)	Q3	Max	n	Min	Q1	Median (95% CI)	Q3	Max
Day 1	19	0.64	0.90	1.06 (0.93,1.13)	1.17	1.83	15	0.08	0.86	0.99 (0.88,1.11)	1.10	1.83	34	0.08	0.88	1.05 (0.93,1.09)	1.12	1.83
Day 2	19	0.73	1.00	1.15 (1.00,1.30)	1.49	2.21	16	0.57	0.96	1.14 (0.95,1.27)	1.27	1.93	35	0.57	1.00	1.15 (1.02,1.25)	1.31	2.21
Day 7	19	0.57	0.95	1.05 (0.95,1.17)	1.25	3.70	16	0.50	0.91	0.96 (0.91,1.13)	1.16	2.06	35	0.50	0.94	0.99 (0.95,1.07)	1.21	3.70
Day 14	19	0.55	0.91	1.10 (0.94,1.23)	1.24	2.00	16	0.24	0.91	0.99 (0.92,1.14)	1.12	1.57	35	0.24	0.90	1.08 (0.94,1.15)	1.20	2.00

Table 38: Descriptive summary statistics of natural killer cells (CD56 dim CD16- CD69-) mean fluorescence intensity fold change from baseline by treatment.

	DVC-LVS						USAMARIID-LVS						Combined					
Study Visit	n	Min	Q1	Median (95% CI)	Q3	Max	n	Min	Q1	Median (95% CI)	Q3	Max	n	Min	Q1	Median (95% CI)	Q3	Max
Day 1	19	0.51	0.77	0.94 (0.77,1.06)	1.11	1.40	15	0.71	0.82	0.91 (0.83,1.03)	1.03	2.43	34	0.51	0.79	0.94 (0.84,1.02)	1.04	2.43
Day 2	19	0.34	0.73	0.82 (0.77,0.99)	0.99	1.96	16	0.65	0.89	0.96 (0.90,1.10)	1.11	2.90	35	0.34	0.78	0.92 (0.82,0.99)	1.06	2.90
Day 7	19	0.50	0.71	0.90 (0.72,1.09)	1.13	2.72	16	0.55	0.79	0.96 (0.79,1.08)	1.08	2.86	35	0.50	0.74	0.90 (0.79,1.03)	1.10	2.86
Day 14	19	0.73	0.83	0.93 (0.83,1.04)	1.04	2.02	16	0.69	0.90	1.07 (0.91,1.12)	1.12	2.58	35	0.69	0.85	0.95 (0.92,1.05)	1.11	2.58

Table 39: Descriptive summary statistics of B-cells (CD69-) mean fluorescence intensity fold change from baseline by treatment.

	DVC-LVS						USAMARIID-LVS						Combined					
Study Visit	n	Min	Q1	Median (95% CI)	Q3	Max	n	Min	Q1	Median (95% CI)	Q3	Max	n	Min	Q1	Median (95% CI)	Q3	Max
Day 1	19	0.88	0.99	1.04 (1.00,1.09)	1.10	1.24	15	0.75	0.89	0.96 (0.91,1.04)	1.04	1.10	34	0.75	0.93	1.01 (0.96,1.04)	1.06	1.24
Day 2	19	0.63	0.98	1.07 (0.99,1.12)	1.30	1.82	16	0.77	0.89	0.97 (0.90,1.09)	1.09	1.52	35	0.63	0.95	1.03 (0.96,1.09)	1.12	1.82
Day 7	19	0.80	0.92	0.97 (0.94,1.08)	1.11	3.47	16	0.07	0.81	0.88 (0.80,0.94)	0.94	1.36	35	0.07	0.85	0.94 (0.88,1.00)	1.04	3.47
Day 14	19	0.54	0.93	1.00 (0.95,1.10)	1.11	1.56	16	0.72	0.75	0.85 (0.75,0.92)	0.92	1.24	35	0.54	0.84	0.93 (0.86,1.02)	1.05	1.56

Table 40: Descriptive summary statistics of monocyte cells (HLA-DR) mean fluorescence intensity fold change from baseline by treatment.

	DVC-LVS						USAMARIID-LVS						Combined					
Study Visit	n	Min	Q1	Median (95% CI)	Q3	Max	n	Min	Q1	Median (95% CI)	Q3	Max	n	Min	Q1	Median (95% CI)	Q3	Max
Day 1	19	0.76	0.90	0.97 (0.91,1.04)	1.05	1.29	15	0.79	0.95	0.99 (0.95,1.08)	1.11	1.52	34	0.76	0.91	0.99 (0.95,1.03)	1.07	1.52
Day 2	19	0.76	0.94	1.03 (0.94,1.10)	1.14	1.46	16	0.80	0.93	1.03 (0.92,1.12)	1.11	1.96	35	0.76	0.94	1.03 (0.95,1.09)	1.12	1.96
Day 7	19	0.68	0.86	0.95 (0.90,1.09)	1.10	4.72	16	0.21	0.75	0.89 (0.75,1.15)	1.15	1.73	35	0.21	0.77	0.90 (0.82,1.07)	1.12	4.72
Day 14	19	0.71	0.90	0.96 (0.90,1.04)	1.07	1.35	16	0.54	0.76	0.93 (0.77,1.14)	1.14	2.06	35	0.54	0.86	0.95 (0.90,1.04)	1.12	2.06

Table 41: Descriptive summary statistics of monocyte cells (CD16+ HLA-DR) mean fluorescence intensity fold change from baseline by treatment.

	DVC-LVS						USAMARIID-LVS						Combined					
Study Visit	n	Min	Q1	Median (95% CI)	Q3	Max	n	Min	Q1	Median (95% CI)	Q3	Max	n	Min	Q1	Median (95% CI)	Q3	Max
Day 1	19	0.92	0.99	1.04 (1.00,1.07)	1.09	1.30	15	0.72	0.87	0.94 (0.89,1.03)	1.02	1.15	34	0.72	0.94	1.02 (0.97,1.05)	1.07	1.30
Day 2	19	0.62	1.02	1.08 (1.02,1.15)	1.31	1.76	16	0.79	0.90	0.97 (0.90,1.13)	1.13	1.42	35	0.62	0.95	1.05 (0.97,1.13)	1.14	1.76
Day 7	19	0.80	0.91	0.98 (0.91,1.08)	1.11	3.36	16	0.06	0.80	0.88 (0.79,0.95)	0.96	1.28	35	0.06	0.86	0.94 (0.89,1.01)	1.02	3.36
Day 14	19	0.52	0.96	1.01 (0.97,1.11)	1.12	1.48	16	0.64	0.79	0.85 (0.78,0.90)	0.89	1.16	35	0.52	0.84	0.94 (0.87,1.05)	1.06	1.48

Table 42: Descriptive summary statistics of monocyte cells (CD16- HLA-DR) mean fluorescence intensity fold change from baseline by treatment.

	DVC-LVS						USAMARIID-LVS						Combined					
Study Visit	n	Min	Q1	Median (95% CI)	Q3	Max	n	Min	Q1	Median (95% CI)	Q3	Max	n	Min	Q1	Median (95% CI)	Q3	Max
Day 1	22	0.22	0.91	1.58 (1.00,1.86)	1.97	5.05	18	0.17	0.55	0.93 (0.56,1.89)	1.90	23.39	40	0.17	0.61	1.25 (0.87,1.79)	1.94	23.39
Day 2	22	0.23	0.89	1.66 (1.07,2.50)	2.79	6.18	18	0.19	0.54	0.90 (0.67,1.99)	2.36	14.28	40	0.19	0.77	1.24 (0.88,1.86)	2.68	14.28
Day 7	22	0.33	0.60	0.85 (0.70,1.16)	1.47	52.21	18	0.24	0.50	0.75 (0.58,2.09)	2.20	13.90	40	0.24	0.55	0.80 (0.70,1.16)	1.83	52.21
Day 14	22	0.15	0.60	1.28 (0.71,1.86)	2.15	50.30	18	0.14	0.52	0.84 (0.53,2.56)	2.69	7.71	40	0.14	0.54	1.08 (0.64,1.64)	2.34	50.30

Table 43: Descriptive summary statistics of Human platelet-derived growth factor concentration fold change from baseline by treatment.

	DVC-LVS						USAMARIID-LVS						Combined					
Study Visit	n	Min	Q1	Median (95% CI)	Q3	Max	n	Min	Q1	Median (95% CI)	Q3	Max	n	Min	Q1	Median (95% CI)	Q3	Max
Day 1	22	1	1	1 (1,1)	1	1	18	1	1	1 (1,1)	1	1	40	1	1	1 (1,1)	1	1
Day 2	22	1	1	1 (1,1)	1	1	18	1	1	1 (1,1)	1	1	40	1	1	1 (1,1)	1	1
Day 7	22	1	1	1 (1,1)	1	1	18	1	1	1 (1,1)	1	1	40	1	1	1 (1,1)	1	1
Day 14	22	1	1	1 (1,1)	1	1	18	1	1	1 (1,1)	1	1	40	1	1	1 (1,1)	1	1

Table 44: Descriptive summary statistics of Human Interleukin-1 beta concentration fold change from baseline by treatment.

	DVC-LVS						USAMARIID-LVS						Combined					
Study Visit	n	Min	Q1	Median (95% CI)	Q3	Max	n	Min	Q1	Median (95% CI)	Q3	Max	n	Min	Q1	Median (95% CI)	Q3	Max
Day 1	22	0.54	0.89	1.23 (0.94,2.09)	2.26	5.42	18	0.24	0.61	1.03 (0.64,1.68)	1.77	5.11	40	0.24	0.74	1.20 (0.90,1.45)	2.10	5.42
Day 2	22	0.30	0.95	1.28 (1.01,2.08)	2.17	4.01	18	0.03	0.76	1.21 (0.78,1.96)	2.14	7.23	40	0.03	0.86	1.21 (1.00,1.74)	2.21	7.23
Day 7	22	0.18	0.58	0.80 (0.64,1.16)	1.30	7.82	18	0.29	0.53	1.05 (0.54,2.12)	2.13	5.10	40	0.18	0.56	0.84 (0.68,1.32)	1.70	7.82
Day 14	22	0.27	0.81	1.12 (0.87,1.66)	1.87	3.61	18	0.17	0.44	1.03 (0.44,1.33)	1.34	4.04	40	0.17	0.70	1.07 (0.87,1.34)	1.79	4.04

Table 45: Descriptive summary statistics of Human Interleukin-1 receptor antagonist concentration fold change from baseline by treatment.

	DVC-LVS						USAMARIID-LVS						Combined					
Study Visit	n	Min	Q1	Median (95% CI)	Q3	Max	n	Min	Q1	Median (95% CI)	Q3	Max	n	Min	Q1	Median (95% CI)	Q3	Max
Day 1	22	0.68	1	1 (1,1)	1	1.45	18	0.38	1	1 (1,1)	1	3.77	40	0.38	1	1 (1,1)	1	3.77

Confidential/Proprietary Information

	DVC-LVS						USAMARIID-LVS						Combined					
Study Visit	n	Min	Q1	Median (95% CI)	Q3	Max	n	Min	Q1	Median (95% CI)	Q3	Max	n	Min	Q1	Median (95% CI)	Q3	Max
Day 2	22	0.41	1	1 (1,1)	1	1.09	18	0.38	1	1 (1,1)	1	3.67	40	0.38	1	1 (1,1)	1	3.67
Day 7	22	0.32	1	1 (1,1)	1	1.18	18	0.38	1	1 (1,1)	1	3.64	40	0.32	1	1 (1,1)	1	3.64
Day 14	22	0.32	1	1 (1,1)	1	3.95	18	0.38	1	1 (1,1)	1	3.44	40	0.32	1	1 (1,1)	1	3.95

Table 46: Descriptive summary statistics of Human Interleukin-2 concentration fold change from baseline by treatment.

	DVC-LVS						USAMARIID-LVS						Combined					
Study Visit	n	Min	Q1	Median (95% CI)	Q3	Max	n	Min	Q1	Median (95% CI)	Q3	Max	n	Min	Q1	Median (95% CI)	Q3	Max
Day 1	22	0.44	1.00	1.12 (1.00,1.61)	1.72	3.61	18	0.35	0.51	1.00 (0.56,1.16)	1.22	5.32	40	0.35	0.93	1.00 (1.00,1.27)	1.54	5.32
Day 2	22	0.73	1.00	1.12 (1.01,1.75)	1.76	3.41	18	0.35	0.79	1.14 (0.81,2.06)	2.06	4.93	40	0.35	0.98	1.12 (1.00,1.38)	1.93	4.93
Day 7	22	0.22	0.45	0.96 (0.54,1.00)	1.00	4.36	18	0.25	0.47	0.84 (0.49,1.80)	2.02	3.02	40	0.22	0.46	0.96 (0.57,1.00)	1.27	4.36
Day 14	22	0.34	0.95	1.00 (0.96,1.38)	1.59	3.61	18	0.18	0.65	0.98 (0.67,1.26)	1.28	3.39	40	0.18	0.90	1.00 (0.95,1.13)	1.58	3.61

Table 47: Descriptive summary statistics of Human Interleukin-4 concentration fold change from baseline by treatment.

	DVC-LVS						USAMARIID-LVS						Combined					
Study Visit	n	Min	Q1	Median (95% CI)	Q3	Max	n	Min	Q1	Median (95% CI)	Q3	Max	n	Min	Q1	Median (95% CI)	Q3	Max
Day 1	22	0.65	1	1 (1,1)	1	2.71	18	0.73	1	1 (1,1)	1	1	40	0.65	1	1 (1,1)	1	2.71
Day 2	22	0.44	1	1 (1,1)	1	1.00	18	0.32	1	1 (1,1)	1	1	40	0.32	1	1 (1,1)	1	1.00
Day 7	22	0.60	1	1 (1,1)	1	1.00	18	0.32	1	1 (1,1)	1	1	40	0.32	1	1 (1,1)	1	1.00
Day 14	22	0.76	1	1 (1,1)	1	2.03	18	0.32	1	1 (1,1)	1	1	40	0.32	1	1 (1,1)	1	2.03

Table 48: Descriptive summary statistics of Human Interleukin-5 concentration fold change from baseline by treatment.

	DVC-LVS						USAMARIID-LVS						Combined					
Study Visit	n	Min	Q1	Median (95% CI)	Q3	Max	n	Min	Q1	Median (95% CI)	Q3	Max	n	Min	Q1	Median (95% CI)	Q3	Max
Day 1	22	1	1	1 (1,1)	1	3.82	18	0.41	1	1 (1,1)	1	4.68	40	0.41	1	1 (1,1)	1	4.68
Day 2	22	1	1	1 (1,1)	1	1.00	18	0.41	1	1 (1,1)	1	5.18	40	0.41	1	1 (1,1)	1	5.18
Day 7	22	1	1	1 (1,1)	1	2.29	18	0.41	1	1 (1,1)	1	4.48	40	0.41	1	1 (1,1)	1	4.48
Day 14	22	1	1	1 (1,1)	1	1.00	18	0.41	1	1 (1,1)	1	4.33	40	0.41	1	1 (1,1)	1	4.33

Table 49: Descriptive summary statistics of Human Interleukin-6 concentration fold change from baseline by treatment.

	DVC-LVS						USAMARIID-LVS						Combined					
Study Visit	n	Min	Q1	Median (95% CI)	Q3	Max	n	Min	Q1	Median (95% CI)	Q3	Max	n	Min	Q1	Median (95% CI)	Q3	Max
Day 1	22	1	1	1 (1,1)	1	3.38	18	1	1	1 (1,1)	1	1.00	40	1	1	1 (1,1)	1	3.38
Day 2	22	1	1	1 (1,1)	1	1.00	18	1	1	1 (1,1)	1	2.02	40	1	1	1 (1,1)	1	2.02
Day 7	22	1	1	1 (1,1)	1	2.05	18	1	1	1 (1,1)	1	1.00	40	1	1	1 (1,1)	1	2.05
Day 14	22	1	1	1 (1,1)	1	1.00	18	1	1	1 (1,1)	1	1.00	40	1	1	1 (1,1)	1	1.00

Table 50: Descriptive summary statistics of Human Interleukin-7 concentration fold change from baseline by treatment.

	DVC-LVS						USAMARIID-LVS						Combined					
Study Visit	n	Min	Q1	Median (95% CI)	Q3	Max	n	Min	Q1	Median (95% CI)	Q3	Max	n	Min	Q1	Median (95% CI)	Q3	Max
Day 1	22	1.00	1	1 (1,1)	1	2.98	18	1	1	1 (1,1)	1	1	40	1.00	1	1 (1,1)	1	2.98

	DVC-LVS						USAMARIID-LVS						Combined					
Study Visit	n	Min	Q1	Median (95% CI)	Q3	Max	n	Min	Q1	Median (95% CI)	Q3	Max	n	Min	Q1	Median (95% CI)	Q3	Max
Day 2	22	0.45	1	1 (1,1)	1	2.31	18	1	1	1 (1,1)	1	1	40	0.45	1	1 (1,1)	1	2.31
Day 7	22	0.97	1	1 (1,1)	1	2.25	18	1	1	1 (1,1)	1	1	40	0.97	1	1 (1,1)	1	2.25
Day 14	22	0.45	1	1 (1,1)	1	2.38	18	1	1	1 (1,1)	1	1	40	0.45	1	1 (1,1)	1	2.38

Table 51: Descriptive summary statistics of Human Interleukin-8 concentration fold change from baseline by treatment.

	DVC-LVS						USAMARIID-LVS						Combined					
Study Visit	n	Min	Q1	Median (95% CI)	Q3	Max	n	Min	Q1	Median (95% CI)	Q3	Max	n	Min	Q1	Median (95% CI)	Q3	Max
Day 1	22	0.03	1	1 (1,1)	1.05	3.60	18	0.16	0.90	1 (0.94,1)	1.00	16.96	40	0.03	1	1 (1,1)	1	16.96
Day 2	22	0.03	1	1 (1,1)	1.00	16.34	18	0.06	1.00	1 (1.00,1)	1.01	29.37	40	0.03	1	1 (1,1)	1	29.37
Day 7	22	0.03	1	1 (1,1)	1.21	35.55	18	0.16	0.84	1 (0.90,1)	1.00	6.30	40	0.03	1	1 (1,1)	1	35.55
Day 14	22	0.03	1	1 (1,1)	1.00	32.64	18	0.06	1.00	1 (1.00,1)	1.00	21.38	40	0.03	1	1 (1,1)	1	32.64

Table 52: Descriptive summary statistics of Human Interleukin-9 concentration fold change from baseline by treatment.

	DVC-LVS						USAMARIID-LVS						Combined					
Study Visit	n	Min	Q1	Median (95% CI)	Q3	Max	n	Min	Q1	Median (95% CI)	Q3	Max	n	Min	Q1	Median (95% CI)	Q3	Max
Day 1	22	0.32	1	1 (1,1)	1	1.30	18	0.45	1	1 (1,1)	1	1.00	40	0.32	1	1 (1,1)	1	1.30
Day 2	22	0.32	1	1 (1,1)	1	1.23	18	0.45	1	1 (1,1)	1	2.17	40	0.32	1	1 (1,1)	1	2.17
Day 7	22	0.32	1	1 (1,1)	1	2.40	18	0.45	1	1 (1,1)	1	1.00	40	0.32	1	1 (1,1)	1	2.40
Day 14	22	0.32	1	1 (1,1)	1	2.74	18	0.45	1	1 (1,1)	1	1.00	40	0.32	1	1 (1,1)	1	2.74

Table 53: Descriptive summary statistics of Human Interleukin-10 concentration fold change from baseline by treatment.

	DVC-LVS						USAMARIID-LVS						Combined					
Study Visit	n	Min	Q1	Median (95% CI)	Q3	Max	n	Min	Q1	Median (95% CI)	Q3	Max	n	Min	Q1	Median (95% CI)	Q3	Max
Day 1	22	0.35	1	1 (1,1)	1	2.67	18	0.30	1	1 (1,1)	1	3.09	40	0.30	1	1 (1,1)	1	3.09
Day 2	22	0.35	1	1 (1,1)	1	2.23	18	0.50	1	1 (1,1)	1	2.02	40	0.35	1	1 (1,1)	1	2.23
Day 7	22	0.35	1	1 (1,1)	1	2.83	18	0.53	1	1 (1,1)	1	2.01	40	0.35	1	1 (1,1)	1	2.83
Day 14	22	0.35	1	1 (1,1)	1	5.58	18	0.24	1	1 (1,1)	1	2.79	40	0.24	1	1 (1,1)	1	5.58

Table 54: Descriptive summary statistics of Human Interleukin-12 (Lupus Ku autoantigen protein p70) concentration fold change from baseline by treatment.

	DVC-LVS						USAMARIID-LVS						Combined					
Study Visit	n	Min	Q1	Median (95% CI)	Q3	Max	n	Min	Q1	Median (95% CI)	Q3	Max	n	Min	Q1	Median (95% CI)	Q3	Max
Day 1	22	0.61	1	1 (1,1)	1	1.12	18	0.34	0.96	1 (0.98,1)	1	5.53	40	0.34	1	1 (1,1)	1	5.53
Day 2	22	0.43	1	1 (1,1)	1	1.09	18	0.40	0.99	1 (1.00,1)	1	3.72	40	0.40	1	1 (1,1)	1	3.72
Day 7	22	0.44	1	1 (1,1)	1	1.61	18	0.40	1.00	1 (1.00,1)	1	2.80	40	0.40	1	1 (1,1)	1	2.80
Day 14	22	0.69	1	1 (1,1)	1	3.16	18	0.40	0.89	1 (0.92,1)	1	5.17	40	0.40	1	1 (1,1)	1	5.17

Table 55: Descriptive summary statistics of Human Interleukin-13 concentration fold change from baseline by treatment.

	DVC-LVS						USAMARIID-LVS						Combined					
Study Visit	n	Min	Q1	Median (95% CI)	Q3	Max	n	Min	Q1	Median (95% CI)	Q3	Max	n	Min	Q1	Median (95% CI)	Q3	Max

	DVC-LVS						USAMARIID-LVS						Combined					
Study Visit	n	Min	Q1	Median (95% CI)	Q3	Max	n	Min	Q1	Median (95% CI)	Q3	Max	n	Min	Q1	Median (95% CI)	Q3	Max
Day 1	22	1	1	1 (1,1)	1	1	18	1	1	1 (1,1)	1	1	40	1	1	1 (1,1)	1	1
Day 2	22	1	1	1 (1,1)	1	1	18	1	1	1 (1,1)	1	1	40	1	1	1 (1,1)	1	1
Day 7	22	1	1	1 (1,1)	1	1	18	1	1	1 (1,1)	1	1	40	1	1	1 (1,1)	1	1
Day 14	22	1	1	1 (1,1)	1	1	18	1	1	1 (1,1)	1	1	40	1	1	1 (1,1)	1	1

Table 56: Descriptive summary statistics of Human Interleukin-15 concentration fold change from baseline by treatment.

	DVC-LVS						USAMARIID-LVS						Combined					
Study Visit	n	Min	Q1	Median (95% CI)	Q3	Max	n	Min	Q1	Median (95% CI)	Q3	Max	n	Min	Q1	Median (95% CI)	Q3	Max
Day 1	22	0.22	1	1 (1,1)	1	2.88	18	0.24	1	1 (1,1)	1	6.28	40	0.22	1	1 (1,1)	1	6.28
Day 2	22	0.22	1	1 (1,1)	1	1.16	18	0.20	1	1 (1,1)	1	1.00	40	0.20	1	1 (1,1)	1	1.16
Day 7	22	0.22	1	1 (1,1)	1	3.88	18	0.24	1	1 (1,1)	1	1.00	40	0.22	1	1 (1,1)	1	3.88
Day 14	22	0.22	1	1 (1,1)	1	15.57	18	0.30	1	1 (1,1)	1	7.25	40	0.22	1	1 (1,1)	1	15.57

Table 57: Descriptive summary statistics of Human Interleukin-17 concentration fold change from baseline by treatment.

	DVC-LVS						USAMARIID-LVS						Combined					
Study Visit	n	Min	Q1	Median (95% CI)	Q3	Max	n	Min	Q1	Median (95% CI)	Q3	Max	n	Min	Q1	Median (95% CI)	Q3	Max
Day 1	22	0.15	1.00	1 (1,1.15)	1.26	39.60	18	0.03	0.76	1 (0.81,1.00)	1.00	42.59	40	0.03	0.95	1 (1,1.00)	1.08	42.59
Day 2	22	0.02	1.00	1 (1,1.38)	1.45	35.34	18	0.03	0.74	1 (0.76,1.70)	2.05	58.82	40	0.02	0.99	1 (1,1.19)	1.70	58.82
Day 7	22	0.02	1.00	1 (1,1.04)	1.08	58.53	18	0.03	0.55	1 (0.61,1.01)	1.02	13.22	40	0.02	0.88	1 (1,1.00)	1.08	58.53
Day 14	22	0.46	0.92	1 (1,1.40)	1.45	72.17	18	0.03	0.53	1 (0.60,1.00)	1.00	40.31	40	0.03	0.80	1 (1,1.00)	1.42	72.17

Table 58: Descriptive summary statistics of Human Eotaxin concentration fold change from baseline by treatment.

	DVC-LVS						USAMARIID-LVS						Combined					
Study Visit	n	Min	Q1	Median (95% CI)	Q3	Max	n	Min	Q1	Median (95% CI)	Q3	Max	n	Min	Q1	Median (95% CI)	Q3	Max
Day 1	22	0.46	0.98	1.06 (1.00,1.27)	1.44	4.47	18	0.19	0.80	1.00 (0.88,1.36)	1.44	7.12	40	0.19	0.93	1.02 (1.00,1.22)	1.50	7.12
Day 2	22	0.46	0.98	1.12 (1.00,1.34)	1.53	3.75	18	0.59	0.75	1.00 (0.78,1.31)	1.32	5.22	40	0.46	0.90	1.03 (0.99,1.28)	1.39	5.22
Day 7	22	0.41	0.80	1.00 (0.81,1.15)	1.23	6.46	18	0.59	0.76	0.98 (0.80,1.60)	1.62	3.59	40	0.41	0.79	1.00 (0.86,1.13)	1.39	6.46
Day 14	22	0.29	0.98	1.06 (1.00,1.27)	1.39	2.71	18	0.19	0.70	0.95 (0.72,1.36)	1.40	3.25	40	0.19	0.89	1.01 (0.97,1.15)	1.40	3.25

Table 59: Descriptive summary statistics of Human basic fibroblast growth factor concentration fold change from baseline by treatment.

	DVC-LVS						USAMARIID-LVS						Combined					
Study Visit	n	Min	Q1	Median (95% CI)	Q3	Max	n	Min	Q1	Median (95% CI)	Q3	Max	n	Min	Q1	Median (95% CI)	Q3	Max
Day 1	22	0.40	1	1 (1,1.54)	1.84	4.03	18	0.23	1	1 (1,1.58)	1.88	3.06	40	0.23	1	1 (1,1)	1.92	4.03
Day 2	22	0.91	1	1 (1,1.18)	1.29	3.78	18	0.15	1	1 (1,1.00)	1.00	2.71	40	0.15	1	1 (1,1)	1.03	3.78
Day 7	22	0.40	1	1 (1,1.00)	1.00	3.80	18	0.15	1	1 (1,1.00)	1.00	2.84	40	0.15	1	1 (1,1)	1.00	3.80
Day 14	22	0.24	1	1 (1,1.74)	2.09	3.33	18	0.15	1	1 (1,1.00)	1.00	2.92	40	0.15	1	1 (1,1)	1.09	3.33

Table 60: Descriptive summary statistics of Human granulocyte-colony stimulating factor concentration fold change from baseline by treatment.

	DVC-LVS						USAMARIID-LVS						Combined					
Study Visit	n	Min	Q1	Median (95% CI)	Q3	Max	n	Min	Q1	Median (95% CI)	Q3	Max	n	Min	Q1	Median (95% CI)	Q3	Max
Day 1	22	0.44	0.88	1.10 (0.92,1.35)	1.42	2.59	18	0.36	0.65	1.02 (0.67,1.75)	1.77	7.71	40	0.36	0.82	1.05 (0.94,1.30)	1.50	7.71
Day 2	22	0.14	0.82	1.04 (0.88,1.51)	1.55	2.36	18	0.40	0.72	0.97 (0.73,1.44)	1.58	7.14	40	0.14	0.73	1.04 (0.90,1.29)	1.56	7.14
Day 7	22	0.14	0.76	0.94 (0.77,1.12)	1.14	4.14	18	0.42	0.66	0.84 (0.67,1.26)	1.27	20.46	40	0.14	0.68	0.92 (0.77,1.09)	1.24	20.46
Day 14	22	0.03	0.81	1.01 (0.88,1.26)	1.34	2.33	18	0.39	0.68	0.90 (0.72,1.70)	1.86	10.94	40	0.03	0.77	1.01 (0.82,1.16)	1.73	10.94

Table 61: Descriptive summary statistics of Human granulocyte macrophage-colony stimulating factor concentration fold change from baseline by treatment.

	DVC-LVS						USAMARIID-LVS						Combined					
Study Visit	n	Min	Q1	Median (95% CI)	Q3	Max	n	Min	Q1	Median (95% CI)	Q3	Max	n	Min	Q1	Median (95% CI)	Q3	Max
Day 1	22	0.40	0.92	1.21 (0.94,1.86)	2.17	5.75	18	0.19	0.44	1.06 (0.58,1.88)	2.10	4.54	40	0.19	0.77	1.21 (0.89,1.57)	2.29	5.75
Day 2	22	0.19	0.92	1.34 (1.00,1.69)	1.98	5.31	18	0.03	0.77	1.16 (0.78,1.77)	1.85	27.75	40	0.03	0.88	1.27 (0.95,1.58)	1.96	27.75
Day 7	22	0.09	0.62	0.82 (0.69,1.00)	1.10	9.71	18	0.20	0.52	1.02 (0.56,1.68)	1.72	20.35	40	0.09	0.59	0.82 (0.66,1.23)	1.63	20.35
Day 14	22	0.03	0.87	1.09 (0.95,1.74)	1.80	4.15	18	0.01	0.52	0.94 (0.55,1.50)	1.63	16.37	40	0.01	0.69	1.07 (0.88,1.21)	1.77	16.37

Table 62: Descriptive summary statistics of Human interferon gamma concentration fold change from baseline by treatment.

	DVC-LVS						USAMARIID-LVS						Combined					
Study Visit	n	Min	Q1	Median (95% CI)	Q3	Max	n	Min	Q1	Median (95% CI)	Q3	Max	n	Min	Q1	Median (95% CI)	Q3	Max
Day 1	22	0.56	0.90	1.22 (0.97,1.43)	1.51	2.07	18	0.65	0.77	1.14 (0.78,1.35)	1.37	18.86	40	0.56	0.81	1.16 (1.00,1.32)	1.49	18.86
Day 2	22	0.54	0.90	1.24 (0.98,2.01)	2.08	4.38	18	0.69	0.92	1.43 (1.02,1.80)	1.86	3.21	40	0.54	0.88	1.26 (1.05,1.60)	2.03	4.38
Day 7	22	0.50	0.90	0.96 (0.91,1.19)	1.39	3.04	18	0.64	0.80	0.98 (0.81,1.20)	1.22	3.18	40	0.50	0.84	0.98 (0.91,1.15)	1.27	3.18
Day 14	22	0.51	0.82	0.97 (0.82,1.24)	1.30	2.83	18	0.40	0.66	0.90 (0.72,1.10)	1.16	4.02	40	0.40	0.78	0.91 (0.82,1.13)	1.25	4.02

Table 63: Descriptive summary statistics of Human Interferon gamma-induced protein 10 concentration fold change from baseline by treatment.

	DVC-LVS						USAMARIID-LVS						Combined					
Study Visit	n	Min	Q1	Median (95% CI)	Q3	Max	n	Min	Q1	Median (95% CI)	Q3	Max	n	Min	Q1	Median (95% CI)	Q3	Max
Day 1	22	0.70	0.86	1.20 (0.94,1.33)	1.40	2.02	18	0.61	0.78	0.98 (0.79,1.15)	1.20	1.39	40	0.61	0.82	1.08 (0.92,1.23)	1.27	2.02
Day 2	22	0.67	0.98	1.08 (1.02,1.22)	1.29	3.86	18	0.50	0.75	1.01 (0.81,1.10)	1.12	1.63	40	0.50	0.90	1.05 (0.98,1.14)	1.21	3.86
Day 7	22	0.35	0.83	0.96 (0.84,1.07)	1.10	7.90	18	0.40	0.71	0.90 (0.72,1.00)	1.00	1.70	40	0.35	0.78	0.94 (0.86,1.00)	1.07	7.90
Day 14	22	0.38	0.84	1.06 (0.88,1.18)	1.20	3.64	18	0.29	0.81	0.96 (0.82,1.12)	1.13	1.55	40	0.29	0.82	1.00 (0.89,1.11)	1.18	3.64

Table 64: Descriptive summary statistics of Human Monocyte chemoattractant protein-1 (Monocyte Chemotactic and Activating Factor) concentration fold change from baseline by treatment.

	DVC-LVS						USAMARIID-LVS						Combined					
Study Visit	n	Min	Q1	Median (95% CI)	Q3	Max	n	Min	Q1	Median (95% CI)	Q3	Max	n	Min	Q1	Median (95% CI)	Q3	Max
Day 1	22	1.00	1	1 (1,1)	1	2.27	18	0.34	1	1 (1,1)	1	2.47	40	0.34	1	1 (1,1)	1	2.47
Day 2	22	0.98	1	1 (1,1)	1	1.00	18	0.34	1	1 (1,1)	1	1.00	40	0.34	1	1 (1,1)	1	1.00
Day 7	22	1.00	1	1 (1,1)	1	2.20	18	0.34	1	1 (1,1)	1	1.00	40	0.34	1	1 (1,1)	1	2.20
Day 14	22	1.00	1	1 (1,1)	1	1.06	18	0.34	1	1 (1,1)	1	2.22	40	0.34	1	1 (1,1)	1	2.22

Table 65: Descriptive summary statistics of Human Macrophage Inflammatory Protein-1 alpha concentration fold change from baseline by treatment.

	DVC-LVS							USAMARIID-LVS							Combined						
Study Visit	n	Min	Q1	Median (95% CI)	Q3	Max	n	Min	Q1	Median (95% CI)	Q3	Max	n	Min	Q1	Median (95% CI)	Q3	Max
Day 1	22	0.48	0.83	1.02 (0.89,1.24)	1.26	2.03	18	0.58	0.90	1.03 (0.91,1.12)	1.12	4.48	40	0.48	0.88	1.02 (0.94,1.16)	1.25	4.48
Day 2	22	0.89	1.08	1.14 (1.10,1.29)	1.48	3.02	18	0.68	0.91	1.03 (0.94,1.29)	1.43	2.38	40	0.68	1.00	1.11 (1.05,1.23)	1.50	3.02
Day 7	22	0.78	0.95	1.11 (0.97,1.18)	1.26	2.44	18	0.45	0.84	0.99 (0.86,1.18)	1.25	2.30	40	0.45	0.91	1.01 (0.96,1.12)	1.29	2.44
Day 14	22	0.38	0.87	1.10 (0.93,1.19)	1.23	3.22	18	0.39	0.76	0.90 (0.78,1.01)	1.01	1.50	40	0.38	0.82	1.00 (0.86,1.11)	1.16	3.22

Table 66: Descriptive summary statistics of Human Macrophage Inflammatory Protein-1 beta concentration fold change from baseline by treatment.

	DVC-LVS							USAMARIID-LVS							Combined						
Study Visit	n	Min	Q1	Median (95% CI)	Q3	Max	n	Min	Q1	Median (95% CI)	Q3	Max	n	Min	Q1	Median (95% CI)	Q3	Max
Day 1	22	0.41	0.97	1.09 (1.01,1.37)	1.49	1.86	18	0.63	0.98	1.14 (1.00,1.34)	1.36	1.73	40	0.41	0.96	1.10 (1.06,1.28)	1.42	1.86
Day 2	22	0.68	0.87	1.06 (0.92,1.24)	1.26	2.00	18	0.50	0.82	1.08 (0.86,1.22)	1.25	2.00	40	0.50	0.83	1.07 (0.95,1.14)	1.27	2.00
Day 7	22	0.32	0.82	0.94 (0.84,1.21)	1.24	1.92	18	0.50	0.83	1.12 (0.87,1.39)	1.40	2.05	40	0.32	0.80	1.00 (0.91,1.23)	1.32	2.05
Day 14	22	0.42	0.87	1.10 (0.94,1.27)	1.44	3.65	18	0.23	0.96	1.07 (0.96,1.24)	1.29	2.25	40	0.23	0.88	1.08 (1.01,1.18)	1.34	3.65

Table 67: Descriptive summary statistics of Human RANTES concentration fold change from baseline by treatment.

	DVC-LVS							USAMARIID-LVS							Combined						
Study Visit	n	Min	Q1	Median (95% CI)	Q3	Max	n	Min	Q1	Median (95% CI)	Q3	Max	n	Min	Q1	Median (95% CI)	Q3	Max
Day 1	22	0.46	1.00	1 (1,1.98)	2.07	3.63	18	0.23	1	1 (1,1.75)	2.12	3.44	40	0.23	1	1 (1,1.15)	2.14	3.63
Day 2	22	0.42	1.00	1 (1,1.58)	1.96	2.54	18	0.23	1	1 (1,1.00)	1.00	6.87	40	0.23	1	1 (1,1.00)	1.40	6.87
Day 7	22	0.36	0.97	1 (1,1.00)	1.00	4.15	18	0.26	1	1 (1,1.00)	1.00	5.85	40	0.26	1	1 (1,1.00)	1.00	5.85
Day 14	22	0.31	1.00	1 (1,1.00)	1.23	2.90	18	0.26	1	1 (1,1.00)	1.00	5.04	40	0.26	1	1 (1,1.00)	1.00	5.04

Table 68: Descriptive summary statistics of Human Tumor necrosis factor - alpha concentration fold change from baseline by treatment.

	DVC-LVS							USAMARIID-LVS							Combined						
Study Visit	n	Min	Q1	Median (95% CI)	Q3	Max	n	Min	Q1	Median (95% CI)	Q3	Max	n	Min	Q1	Median (95% CI)	Q3	Max
Day 1	22	0.22	1.00	1.00 (1,1.24)	1.34	3.48	18	0.48	0.96	1 (0.97,1.21)	1.21	5.06	40	0.22	0.99	1 (1,1.12)	1.32	5.06
Day 2	22	0.22	1.00	1.00 (1,1.31)	1.38	3.24	18	0.27	1.00	1 (1.00,1.08)	1.11	3.13	40	0.22	1.00	1 (1,1.07)	1.29	3.24
Day 7	22	0.22	0.92	1.00 (1,1.00)	1.17	7.24	18	0.17	1.00	1 (1.00,1.03)	1.04	4.86	40	0.17	0.98	1 (1,1.00)	1.06	7.24
Day 14	22	0.22	1.00	1.02 (1,1.44)	1.55	9.08	18	0.39	0.90	1 (0.94,1.00)	1.00	3.96	40	0.22	1.00	1 (1,1.13)	1.50	9.08

Table 69: Descriptive summary statistics of Human Vascular endothelial growth factor concentration fold change from baseline by treatment.

	DVC-LVS							USAMARIID-LVS							Combined						
Study Visit	n	Min	Q1	Median (95% CI)	Q3	Max	n	Min	Q1	Median (95% CI)	Q3	Max	n	Min	Q1	Median (95% CI)	Q3	Max
Day 1	19	0.90	0.94	0.98 (0.94,1.01)	1.02	1.10	15	0.78	0.88	0.93 (0.88,1.04)	1.03	1.11	34	0.78	0.91	0.97 (0.93,1.01)	1.03	1.11
Day 2	19	0.88	0.94	1.00 (0.94,1.03)	1.03	1.12	16	0.78	0.89	0.98 (0.89,1.04)	1.04	1.14	35	0.78	0.92	0.98 (0.94,1.02)	1.04	1.14
Day 7	19	0.66	0.92	0.96 (0.92,0.99)	0.99	1.10	16	0.73	0.87	0.96 (0.88,1.04)	1.04	1.29	35	0.66	0.90	0.96 (0.93,0.99)	1.01	1.29
Day 14	19	0.88	0.94	0.96 (0.94,1.03)	1.04	1.13	16	0.77	0.91	0.97 (0.91,0.99)	0.99	1.03	35	0.77	0.92	0.96 (0.95,1.00)	1.01	1.13

Table 70: Descriptive summary statistics of natural killer cells (CD56 dim CD16- CCR5-) mean fluorescence intensity fold change from baseline by treatment.

Study Visit	DVC-LVS n	Min	Q1	Median (95% CI)	Q3	Max	USAMARIID-LVS n	Min	Q1	Median (95% CI)	Q3	Max	Combined n	Min	Q1	Median (95% CI)	Q3	Max
Day 1	19	0.81	0.88	0.96 (0.91,1.00)	1.00	1.20	15	0.79	0.87	0.92 (0.87,1.03)	1.02	1.07	34	0.79	0.87	0.93 (0.91,1.00)	1.01	1.20
Day 2	19	0.81	0.89	0.97 (0.89,1.00)	1.00	1.03	16	0.78	0.84	0.94 (0.84,0.99)	0.99	1.12	35	0.78	0.87	0.97 (0.88,0.98)	1.00	1.12
Day 7	19	0.78	0.88	0.96 (0.91,0.98)	0.99	1.11	16	0.73	0.83	0.92 (0.83,1.07)	1.08	1.14	35	0.73	0.84	0.95 (0.90,0.98)	1.01	1.14
Day 14	19	0.77	0.95	0.98 (0.96,1.07)	1.07	1.15	16	0.83	0.91	0.95 (0.90,1.00)	1.01	1.12	35	0.77	0.92	0.97 (0.94,1.01)	1.01	1.15

Table 71: Descriptive summary statistics of B-cells (CCR5-) mean fluorescence intensity fold change from baseline by treatment.

Study Visit	DVC-LVS n	Min	Q1	Median (95% CI)	Q3	Max	USAMARIID-LVS n	Min	Q1	Median (95% CI)	Q3	Max	Combined n	Min	Q1	Median (95% CI)	Q3	Max
Day 1	19	0.76	0.88	0.93 (0.88,0.95)	0.96	10.82	15	0.68	0.81	0.87 (0.81,0.99)	0.99	1.46	34	0.68	0.82	0.91 (0.87,0.95)	0.98	10.82
Day 2	19	0.61	0.88	0.93 (0.88,1.10)	1.14	12.05	16	0.67	0.85	0.92 (0.85,1.01)	1.01	1.59	35	0.61	0.86	0.93 (0.88,1.00)	1.05	12.05
Day 7	19	0.75	0.88	0.91 (0.88,0.98)	1.01	18.10	16	0.41	0.76	0.92 (0.79,0.98)	1.00	1.86	35	0.41	0.84	0.92 (0.88,0.96)	1.01	18.10
Day 14	19	0.61	0.76	0.93 (0.78,1.00)	1.06	18.42	16	0.66	0.81	0.95 (0.82,1.04)	1.06	1.51	35	0.61	0.78	0.94 (0.84,1.00)	1.07	18.42

Table 72: Descriptive summary statistics of T-cells (CD69-) mean fluorescence intensity fold change from baseline by treatment.

Study Visit	DVC-LVS n	Min	Q1	Median (95% CI)	Q3	Max	USAMARIID-LVS n	Min	Q1	Median (95% CI)	Q3	Max	Combined n	Min	Q1	Median (95% CI)	Q3	Max
Day 1	19	0.71	0.87	0.92 (0.88,0.97)	0.99	1.49	15	0.26	0.71	0.85 (0.70,0.98)	0.94	1.66	34	0.26	0.75	0.90 (0.85,0.94)	0.98	1.66
Day 2	19	0.56	0.84	0.89 (0.85,1.00)	1.03	5.10	16	0.26	0.80	0.89 (0.79,1.00)	1.01	1.88	35	0.26	0.83	0.89 (0.84,0.98)	1.03	5.10
Day 7	19	0.72	0.83	0.87 (0.84,0.94)	1.01	10.10	16	0.14	0.71	0.87 (0.68,1.01)	1.00	2.68	35	0.14	0.82	0.87 (0.84,0.94)	1.03	10.10
Day 14	19	0.57	0.70	0.94 (0.71,1.03)	1.04	3.19	16	0.26	0.75	0.90 (0.78,1.05)	1.06	1.70	35	0.26	0.70	0.93 (0.81,1.03)	1.05	3.19

Table 73: Descriptive summary statistics of T-cells (CD4+ CD69-) mean fluorescence intensity fold change from baseline by treatment.

Probe Set ID	Gene Name	Gene Description	Log_2 FC	t-Statistic	P	FDR
206513_PM_at	AIM2	absent in melanoma 2	0.72	5.02	<0.0001	0.0248
204972_PM_at	OAS2	2'-5'-oligoadenylate synthetase 2, 69/71kDa	0.61	4.80	<0.0001	0.0248
1568592_PM_at	TRIM69	tripartite motif containing 69	0.59	4.43	<0.0001	0.033
214511_PM_x_at	FCGR1B, FCGR1C	Fc fragment of IgG, high affinity Ib, receptor (CD64),Fc fragment of IgG, high affinity Ic, receptor (CD64), pseudogene	0.70	4.34	0.0001	0.0367
230036_PM_at	SAMD9L	sterile alpha motif domain containing 9-like	0.64	4.31	0.0001	0.0381
202270_PM_at	GBP1	guanylate binding protein 1, interferon-inducible	0.67	4.14	0.0002	0.0475
210166_PM_at	TLR5	toll-like receptor 5	0.60	4.13	0.0002	0.048
226982_PM_at	ELL2	elongation factor, RNA polymerase II, 2	-0.63	-4.19	0.0002	0.0448
1553785_PM_at	RASGEF1B	RasGEF domain family, member 1B	-0.83	-4.21	0.0002	0.0432
225842_PM_at	PHLDA1	pleckstrin homology-like domain, family A, member 1	-0.85	-4.28	0.0001	0.0413
1556499_PM_s_at	COL1A1	collagen, type I, alpha 1	-0.65	-4.43	<0.0001	0.033
207978_PM_s_at	NR4A3	nuclear receptor subfamily 4, group A, member 3	-1.26	-4.60	<0.0001	0.0261
209959_PM_at	NR4A3	nuclear receptor subfamily 4, group A, member 3	-1.27	-4.69	<0.0001	0.0248
210004_PM_at	OLR1	oxidized low density lipoprotein (lectin-like) receptor 1	-1.10	-4.69	<0.0001	0.0248
220612_PM_at		Homo sapiens PRO0641 protein (PRO0641), mRNA.	-0.64	-4.72	<0.0001	0.0248

Confidential/Proprietary Information

Probe Set ID	Gene Name	Gene Description	Log_2 FC	t-Statistic	P	FDR
226099_PM_at	ELL2	elongation factor, RNA polymerase II, 2	-0.68	-4.72	<0.0001	0.0248
205476_PM_at	CCL20	chemokine (C-C motif) ligand 20	-0.99	-4.74	<0.0001	0.0248
217999_PM_s_at	PHLDA1	pleckstrin homology-like domain, family A, member 1	-0.87	-4.76	<0.0001	0.0248
219557_PM_s_at	NRIP3	nuclear receptor interacting protein 3	-0.88	-4.83	<0.0001	0.0248
1557049_PM_at	BTBD19	BTB (POZ) domain containing 19	-0.65	-4.85	<0.0001	0.0248
211434_PM_s_at	CCRL2	chemokine (C-C motif) receptor-like 2	-0.70	-4.91	<0.0001	0.0248
217997_PM_at	PHLDA1	pleckstrin homology-like domain, family A, member 1	-1.14	-4.92	<0.0001	0.0248
205330_PM_at	MN1	meningioma (disrupted in balanced translocation) 1	-0.73	-4.93	<0.0001	0.0248
239876_PM_at			-0.67	-5.00	<0.0001	0.0248
207850_PM_at	CXCL3	chemokine (C-X-C motif) ligand 3	-0.84	-5.00	<0.0001	0.0248
228536_PM_at	PRMT10	protein arginine methyltransferase 10 (putative)	-0.63	-5.00	<0.0001	0.0248
244868_PM_at			-0.72	-5.01	<0.0001	0.0248
1552711_PM_a_at	CYB5D1	cytochrome b5 domain containing 1	-0.59	-5.02	<0.0001	0.0248
209270_PM_at	LAMB3	laminin, beta 3	-0.76	-5.18	<0.0001	0.0248
224978_PM_s_at	USP36	ubiquitin specific peptidase 36	-0.59	-5.40	<0.0001	0.0197
210118_PM_s_at	IL1A	interleukin 1, alpha	-1.32	-5.69	<0.0001	0.0093
209383_PM_at	DDIT3	DNA-damage-inducible transcript 3	-0.82	-5.96	<0.0001	0.0057
1565701_PM_at		Homo sapiens mRNA; cDNA DKFZp451B0818 (from clone DKFZp451B0818)	-0.70	-5.96	<0.0001	0.0057
239451_PM_at			-1.21	-8.01	<0.0001	<0.0001

Table 74: Differentially expressed genes (Combined Study Groups, Day 0 vs. 1). Sorted by descending t-statistic. Probe set annotations were obtained from Affymetrix (HT HG-U133 Plus PM Annotations, Release 34,10/24/13).

Probe Set ID	Gene Name	Gene Description	Log_2 FC	t-Statistic	P	FDR
231578_PM_at	GBP1	guanylate binding protein 1, interferon-inducible	1.37	7.06	<0.0001	0.0002
235175_PM_at	GBP4	guanylate binding protein 4	0.82	6.78	<0.0001	0.0002
223220_PM_s_at	PARP9	poly (ADP-ribose) polymerase family, member 9	0.79	6.67	<0.0001	0.0003
229543_PM_at	OTTHUMG00000159342, RP1-93H18.6	NULL	0.79	6.52	<0.0001	0.0004
AFFX-HUMISGF3A/M97935_MA_at	STAT1	signal transducer and activator of transcription 1, 91kDa	0.82	6.50	<0.0001	0.0004
229625_PM_at	GBP5	guanylate binding protein 5	0.84	6.46	<0.0001	0.0004
212845_PM_at	SAMD4A	sterile alpha motif domain containing 4A	0.85	6.37	<0.0001	0.0005
205931_PM_s_at	CREB5	cAMP responsive element binding protein 5	0.64	6.29	<0.0001	0.0005
202269_PM_x_at	GBP1	guanylate binding protein 1, interferon-inducible	1.08	5.97	<0.0001	0.0009
206513_PM_at	AIM2	absent in melanoma 2	0.88	5.95	<0.0001	0.0009
232375_PM_at		Homo sapiens cDNA FLJ12169 fis, clone MAMMA1000643	0.81	5.93	<0.0001	0.0009

Probe Set ID	Gene Name	Gene Description	Log_2 FC	t-Statistic	P	FDR
216950_PM_s_at	FCGR1A, FCGR1B, FCGR1C	Fc fragment of IgG, high affinity Ia, receptor (CD64),Fc fragment of IgG, high affinity Ib, receptor (CD64),Fc fragment of IgG, high affinity Ic, receptor (CD64), pseudogene	0.94	5.81	<0.0001	0.0011
204439_PM_at	IFI44L	interferon-induced protein 44-like	0.63	5.79	<0.0001	0.0012
212099_PM_at	RHOB	ras homolog family member B	0.59	5.78	<0.0001	0.0012
231577_PM_s_at	GBP1	guanylate binding protein 1, interferon-inducible	1.09	5.71	<0.0001	0.0013
235276_PM_at	EPSTI1	epithelial stromal interaction 1 (breast)	0.60	5.69	<0.0001	0.0014
200986_PM_at	SERPING1	serpin peptidase inhibitor, clade G (C1 inhibitor), member 1	0.99	5.68	<0.0001	0.0014
214511_PM_x_at	FCGR1B, FCGR1C	Fc fragment of IgG, high affinity Ib, receptor (CD64),Fc fragment of IgG, high affinity Ic, receptor (CD64), pseudogene	0.96	5.65	<0.0001	0.0014
AFFX-HUMISGF3A/M97935_MB_at	STAT1	signal transducer and activator of transcription 1, 91kDa	0.76	5.63	<0.0001	0.0015
202270_PM_at	GBP1	guanylate binding protein 1, interferon-inducible	1.02	5.59	<0.0001	0.0015
238581_PM_at	GBP5	guanylate binding protein 5	0.75	5.57	<0.0001	0.0016
229228_PM_at	CREB5, LOC401317	cAMP responsive element binding protein 5,uncharacterized LOC401317	0.63	5.48	<0.0001	0.0019
227609_PM_at	EPSTI1	epithelial stromal interaction 1 (breast)	0.84	5.47	<0.0001	0.002
229450_PM_at	IFIT3	interferon-induced protein with tetratricopeptide repeats 3	0.86	5.45	<0.0001	0.002
218986_PM_s_at	DDX60	DEAD (Asp-Glu-Ala-Asp) box polypeptide 60	0.59	5.40	<0.0001	0.0022
230036_PM_at	SAMD9L	sterile alpha motif domain containing 9-like	0.79	5.32	<0.0001	0.0023
1561738_PM_at		Homo sapiens cDNA: FLJ20968 fis, clone ADSU00702.	0.79	5.30	<0.0001	0.0024
1568592_PM_at	TRIM69	tripartite motif containing 69	0.72	5.30	<0.0001	0.0024
1570541_PM_s_at	GBP1P1	guanylate binding protein 1, interferon-inducible pseudogene 1	0.83	5.25	<0.0001	0.0027
227807_PM_at	PARP9	poly (ADP-ribose) polymerase family, member 9	0.59	5.24	<0.0001	0.0027
239661_PM_at			0.63	5.24	<0.0001	0.0027
226603_PM_at	SAMD9L	sterile alpha motif domain containing 9-like	0.90	5.23	<0.0001	0.0027
226022_PM_at	SASH1	SAM and SH3 domain containing 1	0.67	5.23	<0.0001	0.0027
200628_PM_s_at	WARS	tryptophanyl-tRNA synthetase	0.61	5.20	<0.0001	0.0029
226757_PM_at	IFIT2	interferon-induced protein with tetratricopeptide repeats 2	0.74	5.03	<0.0001	0.004
202687_PM_s_at	TNFSF10	tumor necrosis factor (ligand) superfamily, member 10	0.74	5.02	<0.0001	0.004
236439_PM_at			0.72	5.00	<0.0001	0.0041
214059_PM_at	IFI44	interferon-induced protein 44	0.61	4.97	<0.0001	0.0043
219806_PM_s_at	SMCO4	single-pass membrane protein with coiled-coil domains 4	0.59	4.97	<0.0001	0.0043
217502_PM_at	IFIT2	interferon-induced protein with tetratricopeptide repeats 2	0.78	4.97	<0.0001	0.0043
211864_PM_s_at	MYOF	myoferlin	0.74	4.96	<0.0001	0.0043
1563509_PM_at		Homo sapiens mRNA; cDNA DKFZp313O229 (from clone DKFZp313O229)	0.59	4.93	<0.0001	0.0046
202688_PM_at	TNFSF10	tumor necrosis factor (ligand) superfamily, member 10	0.75	4.82	<0.0001	0.006
232000_PM_at		Homo sapiens cDNA FLJ11508 fis, clone HEMBA1002162	0.64	4.77	<0.0001	0.0067
209417_PM_s_at	IFI35	interferon-induced protein 35	0.59	4.72	<0.0001	0.0071

Probe Set ID	Gene Name	Gene Description	Log_2 FC	t-Statistic	P	FDR
204533_PM_at	CXCL10	chemokine (C-X-C motif) ligand 10	1.08	4.71	<0.0001	0.0071
AFFX-HUMISGF3A/M97935_5_at	STAT1	signal transducer and activator of transcription 1, 91kDa	0.64	4.67	<0.0001	0.0076
204972_PM_at	OAS2	2'-5'-oligoadenylate synthetase 2, 69/71kDa	0.67	4.66	<0.0001	0.0076
219386_PM_s_at	SLAMF8	SLAM family member 8	0.69	4.66	<0.0001	0.0076
214329_PM_x_at	TNFSF10	tumor necrosis factor (ligand) superfamily, member 10	0.94	4.63	<0.0001	0.0079
201798_PM_s_at	MYOF	myoferlin	0.87	4.59	<0.0001	0.0085
238439_PM_at	ANKRD22	ankyrin repeat domain 22	1.04	4.58	<0.0001	0.0086
235574_PM_at	GBP4	guanylate binding protein 4	0.67	4.51	<0.0001	0.01
203153_PM_at	IFIT1	interferon-induced protein with tetratricopeptide repeats 1	0.74	4.43	<0.0001	0.0119
204747_PM_at	IFIT3	interferon-induced protein with tetratricopeptide repeats 3	0.63	4.26	0.0001	0.0157
232383_PM_at	TFEC	transcription factor EC	0.84	4.24	0.0001	0.0162
217518_PM_at	MYOF	myoferlin	0.67	4.14	0.0002	0.0193
242598_PM_at			0.68	4.06	0.0002	0.0218
1563088_PM_a_at	LOC284837	uncharacterized LOC284837	0.68	3.99	0.0003	0.0249
239196_PM_at	ANKRD22	ankyrin repeat domain 22	0.70	3.88	0.0004	0.0313
235643_PM_at	SAMD9L	sterile alpha motif domain containing 9-like	0.72	3.74	0.0006	0.0401
202869_PM_at	OAS1	2'-5'-oligoadenylate synthetase 1, 40/46kDa	0.63	3.64	0.0008	0.0478
227626_PM_at	PAQR8	progestin and adipoQ receptor family member VIII	0.61	3.62	0.0009	0.0492
205476_PM_at	CCL20	chemokine (C-C motif) ligand 20	-1.29	-3.72	0.0007	0.0422
233121_PM_at		Homo sapiens cDNA FLJ12299 fis, clone MAMMA1001851	-0.82	-3.82	0.0005	0.0346
1564150_PM_a_at	C12orf79	chromosome 12 open reading frame 79	-0.62	-3.84	0.0005	0.0334
203395_PM_s_at	HES1	hairy and enhancer of split 1, (Drosophila)	-0.63	-3.84	0.0005	0.0333
232406_PM_at		Homo sapiens cDNA FLJ13731 fis, clone PLACE3000142	-0.62	-3.85	0.0004	0.0328
230233_PM_at			-0.79	-4.00	0.0003	0.0244
223767_PM_at	GPR84	G protein-coupled receptor 84	-0.64	-4.06	0.0002	0.0218
218856_PM_at	TNFRSF21	tumor necrosis factor receptor superfamily, member 21	-0.60	-4.10	0.0002	0.0205
216243_PM_s_at	IL1RN	interleukin 1 receptor antagonist	-0.63	-4.11	0.0002	0.0201
222180_PM_at	OTTHUMG00000178354, RP11-769O8.3	[illegible]	-0.60	-4.13	0.0002	0.0196
226397_PM_s_at		Homo sapiens cDNA: FLJ21028 fis, clone CAE07155	-0.72	-4.13	0.0002	0.0196
227613_PM_at	ZNF331	zinc finger protein 331	-0.80	-4.15	0.0002	0.0192
1565701_PM_at		Homo sapiens mRNA; cDNA DKFZp451B0818 (from clone DKFZp451B0818)	-0.62	-4.22	0.0002	0.0169
223484_PM_at	C15orf48	chromosome 15 open reading frame 48	-0.97	-4.24	0.0001	0.0162
202948_PM_at	IL1R1	interleukin 1 receptor, type I	-0.64	-4.30	0.0001	0.0149
205479_PM_s_at	PLAU	plasminogen activator, urokinase	-0.59	-4.31	0.0001	0.0146
206648_PM_at	ZNF571	zinc finger protein 571	-0.64	-4.34	0.0001	0.0138
217996_PM_at	PHLDA1	pleckstrin homology-like domain, family A, member 1	-1.10	-4.36	<0.0001	0.0134

Probe Set ID	Gene Name	Gene Description	Log_2 FC	t-Statistic	P	FDR
212158_PM_at	SDC2	syndecan 2	-0.82	-4.38	<0.0001	0.0132
202435_PM_s_at	CYP1B1	cytochrome P450, family 1, subfamily B, polypeptide 1	-0.69	-4.40	<0.0001	0.0126
204614_PM_at	SERPINB2	serpin peptidase inhibitor, clade B (ovalbumin), member 2	-1.23	-4.42	<0.0001	0.0121
237718_PM_at	EIF4E	eukaryotic translation initiation factor 4E	-0.81	-4.56	<0.0001	0.0089
217127_PM_at	CTH	cystathionase (cystathionine gamma-lyase)	-0.70	-4.57	<0.0001	0.0088
1565776_PM_at		Homo sapiens mRNA; cDNA DKFZp667M067 (from clone DKFZp667M067)	-0.84	-4.61	<0.0001	0.0081
202437_PM_s_at	CYP1B1	cytochrome P450, family 1, subfamily B, polypeptide 1	-0.81	-4.63	<0.0001	0.008
224978_PM_s_at	USP36	ubiquitin specific peptidase 36	-0.61	-4.65	<0.0001	0.0076
210139_PM_s_at	PMP22	peripheral myelin protein 22	-0.62	-4.66	<0.0001	0.0076
202436_PM_s_at	CYP1B1	cytochrome P450, family 1, subfamily B, polypeptide 1	-0.75	-4.67	<0.0001	0.0076
211434_PM_s_at	CCRL2	chemokine (C-C motif) receptor-like 2	-0.72	-4.74	<0.0001	0.0069
213638_PM_at	PHACTR1	phosphatase and actin regulator 1	-0.99	-4.75	<0.0001	0.0069
210118_PM_s_at	IL1A	interleukin 1, alpha	-1.54	-4.85	<0.0001	0.0057
209803_PM_s_at	PHLDA2	pleckstrin homology-like domain, family A, member 2	-0.78	-4.85	<0.0001	0.0057
209959_PM_at	NR4A3	nuclear receptor subfamily 4, group A, member 3	-1.36	-4.97	<0.0001	0.0043
1555638_PM_a_at	SAMSN1	SAM domain, SH3 domain and nuclear localization signals 1	-0.70	-4.97	<0.0001	0.0043
239876_PM_at			-0.74	-5.00	<0.0001	0.0041
1556499_PM_s_at	COL1A1	collagen, type I, alpha 1	-0.71	-5.01	<0.0001	0.0041
1553785_PM_at	RASGEF1B	RasGEF domain family, member 1B	-0.86	-5.05	<0.0001	0.0039
231972_PM_at		Homo sapiens cDNA: FLJ21028 fis, clone CAE07155	-0.82	-5.06	<0.0001	0.0038
1563621_PM_at		Homo sapiens mRNA; cDNA DKFZp667O0416 (from clone DKFZp667O0416)	-0.73	-5.06	<0.0001	0.0038
223394_PM_at	SERTAD1	SERTA domain containing 1	-0.62	-5.10	<0.0001	0.0036
204567_PM_s_at	ABCG1	ATP-binding cassette, sub-family G (WHITE), member 1	-0.59	-5.10	<0.0001	0.0036
222900_PM_at	NRIP3	nuclear receptor interacting protein 3	-0.80	-5.14	<0.0001	0.0033
227099_PM_s_at	C11orf96	chromosome 11 open reading frame 96	-0.68	-5.25	<0.0001	0.0027
205330_PM_at	MN1	meningioma (disrupted in balanced translocation) 1	-0.77	-5.32	<0.0001	0.0023
217998_PM_at	PHLDA1	pleckstrin homology-like domain, family A, member 1	-0.86	-5.37	<0.0001	0.0022
210004_PM_at	OLR1	oxidized low density lipoprotein (lectin-like) receptor 1	-1.48	-5.37	<0.0001	0.0022
217997_PM_at	PHLDA1	pleckstrin homology-like domain, family A, member 1	-1.19	-5.39	<0.0001	0.0022
244044_PM_at			-0.66	-5.39	<0.0001	0.0022
201490_PM_s_at	PPIF	peptidylprolyl isomerase F	-0.68	-5.41	<0.0001	0.0022
228536_PM_at	PRMT10	protein arginine methyltransferase 10 (putative)	-0.66	-5.43	<0.0001	0.0021
1557049_PM_at	BTBD19	BTB (POZ) domain containing 19	-0.82	-5.49	<0.0001	0.0019
226099_PM_at	ELL2	elongation factor, RNA polymerase II, 2	-0.71	-5.57	<0.0001	0.0016
217999_PM_s_at	PHLDA1	pleckstrin homology-like domain, family A, member 1	-0.98	-5.66	<0.0001	0.0014
209324_PM_s_at	RGS16	regulator of G-protein signaling 16	-0.84	-5.68	<0.0001	0.0014
231779_PM_at	IRAK2	interleukin-1 receptor-associated kinase 2	-0.68	-5.73	<0.0001	0.0013
244868_PM_at			-0.81	-5.82	<0.0001	0.0011
204790_PM_at	SMAD7	SMAD family member 7	-0.70	-5.84	<0.0001	0.0011

Probe Set ID	Gene Name	Gene Description	Log_2 FC	t-Statistic	P	FDR
207978_PM_s_at	NR4A3	nuclear receptor subfamily 4, group A, member 3	-1.72	-5.87	<0.0001	0.001
219557_PM_s_at	NRIP3	nuclear receptor interacting protein 3	-1.14	-5.94	<0.0001	0.0009
237204_PM_at		ESTs, Moderately similar to OVCA1	-0.66	-5.97	<0.0001	0.0009
226833_PM_at	CYB5D1	cytochrome b5 domain containing 1	-0.77	-5.99	<0.0001	0.0009
225842_PM_at	PHLDA1	pleckstrin homology-like domain, family A, member 1	-1.02	-6.05	<0.0001	0.0009
1565868_PM_at	CD44	CD44 molecule (Indian blood group)	-0.67	-6.29	<0.0001	0.0005
227029_PM_at	FAM177A1	family with sequence similarity 177, member A1	-0.65	-6.39	<0.0001	0.0005
209270_PM_at	LAMB3	laminin, beta 3	-1.03	-6.88	<0.0001	0.0002
209383_PM_at	DDIT3	DNA-damage-inducible transcript 3	-0.92	-6.95	<0.0001	0.0002
203411_PM_s_at	LMNA	lamin A/C	-0.64	-7.02	<0.0001	0.0002
239451_PM_at			-1.28	-7.47	<0.0001	0.0002

Table 75: Differentially expressed genes (Combined Study Groups, Day 0 vs. 2). Sorted by descending t-statistic. Probe set annotations were obtained from Affymetrix (HT HG-U133 Plus PM Annotations, Release 34,10/24/13).

Probe Set ID	Gene Name	Gene Description	Log_2 FC	t-Statistic	P	FDR
209773_PM_s_at	RRM2	ribonucleotide reductase M2	1.01	6.09	<0.0001	0.0004
227618_PM_at			0.74	5.87	<0.0001	0.0006
229228_PM_at	CREB5, LOC401317	cAMP responsive element binding protein 5,uncharacterized LOC401317	0.65	5.81	<0.0001	0.0006
229510_PM_at	MS4A14	membrane-spanning 4-domains, subfamily A, member 14	0.68	5.73	<0.0001	0.0007
1557302_PM_at	ZNF585B	zinc finger protein 585B	0.62	5.58	<0.0001	0.0009
213626_PM_at	CBR4	carbonyl reductase 4	0.63	5.42	<0.0001	0.0012
229670_PM_at			0.66	5.35	<0.0001	0.0014
227038_PM_at	SGMS2	sphingomyelin synthase 2	0.61	5.31	<0.0001	0.0015
202589_PM_at	TYMS	thymidylate synthetase	0.68	5.28	<0.0001	0.0016
225509_PM_at	SAP30L	SAP30-like	0.59	5.22	<0.0001	0.0018
1562289_PM_at		Homo sapiens mRNA; cDNA DKFZp434N0220 (from clone DKFZp434N0220)	0.59	5.22	<0.0001	0.0019
202503_PM_s_at	KIAA0101	KIAA0101	0.78	5.21	<0.0001	0.0019
213653_PM_at	METTL3	methyltransferase like 3	0.60	5.20	<0.0001	0.0019
226603_PM_at	SAMD9L	sterile alpha motif domain containing 9-like	0.81	5.19	<0.0001	0.0019
228157_PM_at	ZNF207	zinc finger protein 207	0.63	5.13	<0.0001	0.002
1554696_PM_s_at	TYMS	thymidylate synthetase	0.59	5.07	<0.0001	0.0022
1563509_PM_at		Homo sapiens mRNA; cDNA DKFZp313O229 (from clone DKFZp313O229)	0.60	5.06	<0.0001	0.0022
228455_PM_at			0.60	5.04	<0.0001	0.0022
1566557_PM_at	BAIAP2-AS1	BAIAP2 antisense RNA 1 (head to head)	0.62	4.94	<0.0001	0.0028
209200_PM_at	MEF2C	myocyte enhancer factor 2C	0.66	4.91	<0.0001	0.0029
230036_PM_at	SAMD9L	sterile alpha motif domain containing 9-like	0.68	4.84	<0.0001	0.0034
1555882_PM_at	SPIN3	spindlin family, member 3	0.62	4.81	<0.0001	0.0036

Probe Set ID	Gene Name	Gene Description	Log_2 FC	t-Statistic	P	FDR
232383_PM_at	TFEC	transcription factor EC	0.79	4.80	<0.0001	0.0036
201890_PM_at	RRM2	ribonucleotide reductase M2	0.65	4.77	<0.0001	0.0037
206513_PM_at	AIM2	absent in melanoma 2	0.67	4.76	<0.0001	0.0038
240061_PM_at			0.67	4.72	<0.0001	0.0041
223155_PM_at	HDHD2	haloacid dehalogenase-like hydrolase domain containing 2	0.61	4.70	<0.0001	0.0042
213830_PM_at	YME1L1	YME1-like 1 ATPase	0.61	4.68	<0.0001	0.0044
232000_PM_at		Homo sapiens cDNA FLJ11508 fis, clone HEMBA1002162	0.69	4.68	<0.0001	0.0044
227626_PM_at	PAQR8	progestin and adipoQ receptor family member VIII	0.72	4.59	<0.0001	0.0053
213238_PM_at	ATP10D	ATPase, class V, type 10D	0.62	4.56	<0.0001	0.0055
243709_PM_at	SLC38A9	solute carrier family 38, member 9	0.60	4.49	<0.0001	0.0062
235625_PM_at	VPS41	vacuolar protein sorting 41 homolog (S. cerevisiae)	0.60	4.49	<0.0001	0.0063
239301_PM_at			0.63	4.44	<0.0001	0.0067
242598_PM_at			0.81	4.42	<0.0001	0.007
243824_PM_at	LOC100996870	uncharacterized LOC100996870	0.77	4.42	<0.0001	0.007
224962_PM_at	C9orf69	chromosome 9 open reading frame 69	0.60	4.42	<0.0001	0.0071
1555037_PM_a_at	IDH1	isocitrate dehydrogenase 1 (NADP+), soluble	0.59	4.37	<0.0001	0.0077
210166_PM_at	TLR5	toll-like receptor 5	0.67	4.34	0.0001	0.008
230918_PM_at			0.68	4.34	0.0001	0.008
241917_PM_at			0.67	4.28	0.0001	0.0089
1552386_PM_at	GAPT	GRB2-binding adaptor protein, transmembrane	0.63	4.26	0.0001	0.0092
227052_PM_at			0.61	4.24	0.0001	0.0095
219243_PM_at	GIMAP4	GTPase, IMAP family member 4	0.61	4.19	0.0002	0.01
226423_PM_at	PAQR8	progestin and adipoQ receptor family member VIII	0.71	4.18	0.0002	0.0103
222691_PM_at	SLC35B3	solute carrier family 35, member B3	0.65	4.15	0.0002	0.0107
1563088_PM_a_at	LOC284837	uncharacterized LOC284837	0.70	4.12	0.0002	0.0114
224358_PM_s_at	MS4A7	membrane-spanning 4-domains, subfamily A, member 7	0.62	4.07	0.0002	0.0123
240238_PM_at			0.63	3.96	0.0003	0.0149
227426_PM_at	SOS1	son of sevenless homolog 1 (Drosophila)	0.63	3.94	0.0003	0.0152
221895_PM_at	MOSPD2	motile sperm domain containing 2	0.61	3.92	0.0004	0.0159
218230_PM_at	ARFIP1	ADP-ribosylation factor interacting protein 1	0.67	3.90	0.0004	0.0164
205898_PM_at	CX3CR1	chemokine (C-X3-C motif) receptor 1	0.86	3.87	0.0004	0.017
202869_PM_at	OAS1	2'-5'-oligoadenylate synthetase 1, 40/46kDa	0.61	3.86	0.0004	0.0176
205552_PM_s_at	OAS1	2'-5'-oligoadenylate synthetase 1, 40/46kDa	0.61	3.82	0.0005	0.0189
209995_PM_s_at	TCL1A	T-cell leukemia/lymphoma 1A	0.75	3.80	0.0005	0.0194
39318_PM_at	TCL1A	T-cell leukemia/lymphoma 1A	0.68	3.78	0.0006	0.0201
228190_PM_at	ATG4C	autophagy related 4C, cysteine peptidase	0.61	3.74	0.0006	0.0214
214329_PM_x_at	TNFSF10	tumor necrosis factor (ligand) superfamily, member 10	0.72	3.73	0.0006	0.0217
231956_PM_at	RNF213	ring finger protein 213	0.61	3.68	0.0007	0.0239
224989_PM_at		Homo sapiens cDNA FLJ13289 fis, clone OVARC1001170	0.62	3.64	0.0008	0.0252

Probe Set ID	Gene Name	Gene Description	Log_2 FC	t-Statistic	P	FDR
206978_PM_at	CCR2	chemokine (C-C motif) receptor 2	0.74	3.63	0.0009	0.0256
216050_PM_at		Homo sapiens cDNA: FLJ20931 fis, clone ADSE01282	0.68	3.56	0.001	0.029
206641_PM_at	TNFRSF17	tumor necrosis factor receptor superfamily, member 17	0.62	3.41	0.0016	0.0374
205220_PM_at	HCAR3	hydroxycarboxylic acid receptor 3	-0.62	-3.24	0.0025	0.05
211924_PM_s_at	PLAUR	plasminogen activator, urokinase receptor	-0.62	-3.30	0.0022	0.0452
218880_PM_at	FOSL2	FOS-like antigen 2	-0.74	-3.33	0.002	0.0427
204103_PM_at	CCL4	chemokine (C-C motif) ligand 4	-0.70	-3.35	0.0019	0.0413
211302_PM_s_at	PDE4B	phosphodiesterase 4B, cAMP-specific	-0.70	-3.36	0.0018	0.0411
202638_PM_s_at	ICAM1	intercellular adhesion molecule 1	-0.65	-3.38	0.0017	0.0395
1555167_PM_s_at	NAMPT	nicotinamide phosphoribosyltransferase	-0.63	-3.41	0.0016	0.0373
225612_PM_s_at	B3GNT5	UDP-GlcNAc:betaGal beta-1,3-N-acetylglucosaminyltransferase 5	-0.70	-3.42	0.0015	0.0369
202637_PM_s_at	ICAM1	intercellular adhesion molecule 1	-0.66	-3.44	0.0015	0.036
244840_PM_x_at	DOCK4	dedicator of cytokinesis 4	-0.76	-3.44	0.0015	0.0356
233899_PM_x_at	ZBTB10	zinc finger and BTB domain containing 10	-0.63	-3.44	0.0014	0.0355
213146_PM_at	KDM6B	lysine (K)-specific demethylase 6B	-0.61	-3.46	0.0014	0.0345
1569477_PM_at		Homo sapiens, clone IMAGE:4291396, mRNA	-0.65	-3.59	0.001	0.0271
203504_PM_s_at	ABCA1	ATP-binding cassette, sub-family A (ABC1), member 1	-0.74	-3.63	0.0009	0.0256
37028_PM_at	PPP1R15A	protein phosphatase 1, regulatory subunit 15A	-0.61	-3.64	0.0008	0.0253
240859_PM_at	ZFYVE16	zinc finger, FYVE domain containing 16	-0.59	-3.64	0.0008	0.0252
201325_PM_s_at	EMP1	epithelial membrane protein 1	-0.64	-3.65	0.0008	0.0249
235592_PM_at			-0.96	-3.66	0.0008	0.0244
1565776_PM_at		Homo sapiens mRNA; cDNA DKFZp667M067 (from clone DKFZp667M067)	-0.80	-3.66	0.0008	0.0244
237496_PM_at		ESTs, Weakly similar to ALU7_HUMAN ALU SUBFAMILY SQ SEQUENCE CONTAMINATION WARNING ENTRY (H.sapiens)	-0.71	-3.67	0.0008	0.0244
226034_PM_at	DUSP4	dual specificity phosphatase 4	-0.77	-3.67	0.0008	0.0242
206025_PM_s_at	TNFAIP6	tumor necrosis factor, alpha-induced protein 6	-0.80	-3.68	0.0007	0.0237
202948_PM_at	IL1R1	interleukin 1 receptor, type I	-0.61	-3.70	0.0007	0.0232
230380_PM_at	THAP2	THAP domain containing, apoptosis associated protein 2	-0.59	-3.70	0.0007	0.0232
225955_PM_at	METRNL	meteorin, glial cell differentiation regulator-like	-0.61	-3.75	0.0006	0.021
206157_PM_at	PTX3	pentraxin 3, long	-0.74	-3.77	0.0006	0.0205
1564093_PM_at	NEK1	NIMA-related kinase 1	-0.70	-3.82	0.0005	0.0188
1554786_PM_at	CASS4	Cas scaffolding protein family member 4	-0.70	-3.94	0.0003	0.0152
201631_PM_s_at	IER3	immediate early response 3	-0.64	-3.95	0.0003	0.0149
240103_PM_at	LOC100996457	uncharacterized LOC100996457	-1.02	-3.96	0.0003	0.0148
243659_PM_at			-0.87	-3.97	0.0003	0.0147
1558691_PM_a_at	DOCK4	dedicator of cytokinesis 4	-0.78	-3.99	0.0003	0.0143
242727_PM_at	ARL5B	ADP-ribosylation factor-like 5B	-0.68	-4.01	0.0003	0.0137
229437_PM_at	MIR155, MIR155HG	microRNA 155,MIR155 host gene (non-protein coding)	-0.60	-4.01	0.0003	0.0137

Probe Set ID	Gene Name	Gene Description	Log_2 FC	t-Statistic	P	FDR
207850_PM_at	CXCL3	chemokine (C-X-C motif) ligand 3	-1.08	-4.02	0.0003	0.0135
219312_PM_s_at	ZBTB10	zinc finger and BTB domain containing 10	-0.72	-4.07	0.0002	0.0123
1564150_PM_a_at	C12orf79	chromosome 12 open reading frame 79	-0.68	-4.09	0.0002	0.0122
202014_PM_at	PPP1R15A	protein phosphatase 1, regulatory subunit 15A	-0.75	-4.10	0.0002	0.0118
230127_PM_at	OTTHUMG00000184015, RP6-99M1.2	NULL	-0.65	-4.12	0.0002	0.0115
205027_PM_s_at	MAP3K8	mitogen-activated protein kinase kinase kinase 8	-0.62	-4.17	0.0002	0.0104
224836_PM_at	TP53INP2	tumor protein p53 inducible nuclear protein 2	-0.80	-4.18	0.0002	0.0102
210954_PM_s_at	TSC22D2	TSC22 domain family, member 2	-0.61	-4.19	0.0002	0.0101
230511_PM_at	CREM	cAMP responsive element modulator	-0.99	-4.19	0.0002	0.0101
209967_PM_s_at	CREM	cAMP responsive element modulator	-0.91	-4.20	0.0002	0.01
1554980_PM_a_at	ATF3	activating transcription factor 3	-0.70	-4.22	0.0002	0.0097
240207_PM_at			-0.61	-4.23	0.0001	0.0096
220612_PM_at		Homo sapiens PRO0641 protein (PRO0641), mRNA.	-0.66	-4.23	0.0001	0.0096
203505_PM_at	ABCA1	ATP-binding cassette, sub-family A (ABC1), member 1	-0.68	-4.24	0.0001	0.0095
205193_PM_at	MAFF	v-maf musculoaponeurotic fibrosarcoma oncogene homolog F (avian)	-0.96	-4.26	0.0001	0.0093
202933_PM_s_at	YES1	v-yes-1 Yamaguchi sarcoma viral oncogene homolog 1	-0.60	-4.28	0.0001	0.0089
203888_PM_at	THBD	thrombomodulin	-0.95	-4.29	0.0001	0.0088
208868_PM_s_at	GABARAPL1	GABA(A) receptor-associated protein like 1	-0.64	-4.31	0.0001	0.0085
204014_PM_at	DUSP4	dual specificity phosphatase 4	-0.64	-4.32	0.0001	0.0083
215889_PM_at	SKIL	SKI-like oncogene	-0.71	-4.34	0.0001	0.008
237252_PM_at	THBD	thrombomodulin	-0.67	-4.34	0.0001	0.008
203887_PM_s_at	THBD	thrombomodulin	-0.98	-4.36	0.0001	0.0078
201324_PM_at	EMP1	epithelial membrane protein 1	-0.85	-4.40	<0.0001	0.0073
202437_PM_s_at	CYP1B1	cytochrome P450, family 1, subfamily B, polypeptide 1	-0.86	-4.40	<0.0001	0.0073
204363_PM_at	F3	coagulation factor III (thromboplastin, tissue factor)	-0.65	-4.41	<0.0001	0.0071
225539_PM_at	ZBTB21	zinc finger and BTB domain containing 21	-0.66	-4.47	<0.0001	0.0064
219228_PM_at	ZNF331	zinc finger protein 331	-1.03	-4.47	<0.0001	0.0064
36711_PM_at	MAFF	v-maf musculoaponeurotic fibrosarcoma oncogene homolog F (avian)	-0.98	-4.48	<0.0001	0.0063
208869_PM_s_at	GABARAPL1	GABA(A) receptor-associated protein like 1	-0.64	-4.49	<0.0001	0.0063
206983_PM_at	CCR6	chemokine (C-C motif) receptor 6	-0.59	-4.49	<0.0001	0.0062
209099_PM_x_at	JAG1	jagged 1	-0.66	-4.50	<0.0001	0.0062
216066_PM_at	ABCA1	ATP-binding cassette, sub-family A (ABC1), member 1	-0.60	-4.51	<0.0001	0.0061
203394_PM_s_at	HES1	hairy and enhancer of split 1, (Drosophila)	-0.72	-4.51	<0.0001	0.0061
211840_PM_s_at	PDE4D	phosphodiesterase 4D, cAMP-specific	-0.64	-4.53	<0.0001	0.0059
225754_PM_at	AP1G1	adaptor-related protein complex 1, gamma 1 subunit	-0.66	-4.58	<0.0001	0.0054
240024_PM_at	SEC14L2	SEC14-like 2 (S. cerevisiae)	-0.62	-4.59	<0.0001	0.0053
202988_PM_s_at	RGS1	regulator of G-protein signaling 1	-0.83	-4.64	<0.0001	0.0048
210118_PM_s_at	IL1A	interleukin 1, alpha	-1.48	-4.68	<0.0001	0.0044

Probe Set ID	Gene Name	Gene Description	Log_2 FC	t-Statistic	P	FDR
202932_PM_at	YES1	v-yes-1 Yamaguchi sarcoma viral oncogene homolog 1	-0.73	-4.71	<0.0001	0.0042
202684_PM_s_at	RNMT	RNA (guanine-7-) methyltransferase	-0.62	-4.72	<0.0001	0.0041
205476_PM_at	CCL20	chemokine (C-C motif) ligand 20	-1.64	-4.72	<0.0001	0.0041
202435_PM_s_at	CYP1B1	cytochrome P450, family 1, subfamily B, polypeptide 1	-0.76	-4.73	<0.0001	0.004
243771_PM_at			-0.70	-4.74	<0.0001	0.0039
222670_PM_s_at	MAFB	v-maf musculoaponeurotic fibrosarcoma oncogene homolog B (avian)	-0.68	-4.76	<0.0001	0.0038
233952_PM_s_at	ZBTB21	zinc finger and BTB domain containing 21	-0.67	-4.77	<0.0001	0.0038
214508_PM_x_at	CREM	cAMP responsive element modulator	-0.92	-4.78	<0.0001	0.0037
1554929_PM_at	SIK3	SIK family kinase 3	-0.62	-4.78	<0.0001	0.0037
239827_PM_at	RGCC	regulator of cell cycle	-0.96	-4.78	<0.0001	0.0037
227140_PM_at	INHBA	inhibin, beta A	-0.74	-4.79	<0.0001	0.0036
202436_PM_s_at	CYP1B1	cytochrome P450, family 1, subfamily B, polypeptide 1	-0.79	-4.80	<0.0001	0.0036
204567_PM_s_at	ABCG1	ATP-binding cassette, sub-family G (WHITE), member 1	-0.72	-4.80	<0.0001	0.0036
212659_PM_s_at	IL1RN	interleukin 1 receptor antagonist	-0.78	-4.80	<0.0001	0.0036
207630_PM_s_at	CREM	cAMP responsive element modulator	-1.06	-4.81	<0.0001	0.0035
210793_PM_s_at	NUP98	nucleoporin 98kDa	-0.59	-4.84	<0.0001	0.0034
230218_PM_at	HIC1	hypermethylated in cancer 1	-0.70	-4.85	<0.0001	0.0034
217996_PM_at	PHLDA1	pleckstrin homology-like domain, family A, member 1	-1.44	-4.90	<0.0001	0.0031
218856_PM_at	TNFRSF21	tumor necrosis factor receptor superfamily, member 21	-0.76	-4.92	<0.0001	0.0029
218631_PM_at	AVPI1	arginine vasopressin-induced 1	-0.62	-4.93	<0.0001	0.0028
206374_PM_at	DUSP8	dual specificity phosphatase 8	-0.69	-4.96	<0.0001	0.0026
1554095_PM_at	RBM33	RNA binding motif protein 33	-0.65	-4.97	<0.0001	0.0026
227093_PM_at	USP36	ubiquitin specific peptidase 36	-0.67	-5.01	<0.0001	0.0024
1557166_PM_at	PDCD4	programmed cell death 4 (neoplastic transformation inhibitor)	-0.82	-5.03	<0.0001	0.0023
227099_PM_s_at	C11orf96	chromosome 11 open reading frame 96	-0.74	-5.05	<0.0001	0.0022
244054_PM_at			-0.90	-5.05	<0.0001	0.0022
209325_PM_s_at	RGS16	regulator of G-protein signaling 16	-0.60	-5.06	<0.0001	0.0022
1554906_PM_a_at	MPHOSPH6	M-phase phosphoprotein 6	-0.71	-5.10	<0.0001	0.0021
1556676_PM_a_at	OTTHUMG000001[illegible], RP11-425D10.10	[illegible]	-0.66	-5.12	<0.0001	0.002
205479_PM_s_at	PLAU	plasminogen activator, urokinase	-0.72	-5.12	<0.0001	0.002
212158_PM_at	SDC2	syndecan 2	-0.97	-5.13	<0.0001	0.002
210837_PM_s_at	PDE4D	phosphodiesterase 4D, cAMP-specific	-0.86	-5.15	<0.0001	0.002
1570432_PM_at		Homo sapiens clone pp10199 unknown mRNA.	-0.77	-5.16	<0.0001	0.002
243605_PM_at			-0.66	-5.16	<0.0001	0.002
1552711_PM_a_at	CYB5D1	cytochrome b5 domain containing 1	-0.63	-5.16	<0.0001	0.002
204491_PM_at	PDE4D	phosphodiesterase 4D, cAMP-specific	-0.74	-5.16	<0.0001	0.002
230652_PM_at	ARAF	v-raf murine sarcoma 3611 viral oncogene homolog	-0.76	-5.17	<0.0001	0.0019

Probe Set ID	Gene Name	Gene Description	Log_2 FC	t-Statistic	P	FDR
231972_PM_at		Homo sapiens cDNA: FLJ21028 fis, clone CAE07155	-0.81	-5.20	<0.0001	0.0019
204015_PM_s_at	DUSP4	dual specificity phosphatase 4	-0.81	-5.20	<0.0001	0.0019
201195_PM_s_at	SLC7A5	solute carrier family 7 (amino acid transporter light chain, L system), member 5	-0.96	-5.20	<0.0001	0.0019
244868_PM_at			-0.84	-5.20	<0.0001	0.0019
215485_PM_s_at	ICAM1	intercellular adhesion molecule 1	-0.70	-5.21	<0.0001	0.0019
226833_PM_at	CYB5D1	cytochrome b5 domain containing 1	-0.77	-5.26	<0.0001	0.0017
1554600_PM_s_at	LMNA	lamin A/C	-0.60	-5.29	<0.0001	0.0016
1555638_PM_a_at	SAMSN1	SAM domain, SH3 domain and nuclear localization signals 1	-0.88	-5.33	<0.0001	0.0015
201489_PM_at	PPIF	peptidylprolyl isomerase F	-0.71	-5.36	<0.0001	0.0013
239835_PM_at	KBTBD8	kelch repeat and BTB (POZ) domain containing 8	-0.77	-5.38	<0.0001	0.0013
227613_PM_at	ZNF331	zinc finger protein 331	-1.24	-5.40	<0.0001	0.0012
241985_PM_at	JMY	junction mediating and regulatory protein, p53 cofactor	-0.90	-5.41	<0.0001	0.0012
221563_PM_at	DUSP10	dual specificity phosphatase 10	-0.61	-5.42	<0.0001	0.0012
227333_PM_at		Homo sapiens cDNA: FLJ23546 fis, clone LNG08361	-0.59	-5.42	<0.0001	0.0012
223767_PM_at	GPR84	G protein-coupled receptor 84	-0.90	-5.44	<0.0001	0.0012
204087_PM_s_at	SLC5A6	solute carrier family 5 (sodium-dependent vitamin transporter), member 6	-0.62	-5.45	<0.0001	0.0011
226922_PM_at	RANBP2	RAN binding protein 2	-0.60	-5.45	<0.0001	0.0011
223028_PM_s_at	SNX9	sorting nexin 9	-0.62	-5.47	<0.0001	0.0011
219199_PM_at	AFF4	AF4/FMR2 family, member 4	-0.63	-5.49	<0.0001	0.0011
1556499_PM_s_at	COL1A1	collagen, type I, alpha 1	-0.89	-5.54	<0.0001	0.0009
217127_PM_at	CTH	cystathionase (cystathionine gamma-lyase)	-0.77	-5.54	<0.0001	0.0009
239567_PM_at			-0.72	-5.54	<0.0001	0.0009
230233_PM_at			-1.24	-5.56	<0.0001	0.0009
217997_PM_at	PHLDA1	pleckstrin homology-like domain, family A, member 1	-1.44	-5.57	<0.0001	0.0009
210836_PM_x_at	PDE4D	phosphodiesterase 4D, cAMP-specific	-0.76	-5.57	<0.0001	0.0009
204614_PM_at	SERPINB2	serpin peptidase inhibitor, clade B (ovalbumin), member 2	-1.72	-5.58	<0.0001	0.0009
228536_PM_at	PRMT10	protein arginine methyltransferase 10 (putative)	-0.76	-5.59	<0.0001	0.0009
218851_PM_s_at	SFT2D3, WDR33	SFT2 domain containing 3,WD repeat domain 33	-0.73	-5.59	<0.0001	0.0009
211423_PM_s_at	SC5D	sterol-C5-desaturase	-0.70	-5.61	<0.0001	0.0009
202068_PM_s_at	LDLR	low density lipoprotein receptor	-0.69	-5.62	<0.0001	0.0009
244578_PM_at	LCP2	lymphocyte cytosolic protein 2 (SH2 domain containing leukocyte protein of 76kDa)	-0.68	-5.66	<0.0001	0.0008
206648_PM_at	ZNF571	zinc finger protein 571	-0.96	-5.67	<0.0001	0.0008
223887_PM_at	GPR132	G protein-coupled receptor 132	-0.75	-5.68	<0.0001	0.0008
204435_PM_at	NUPL1	nucleoporin like 1	-0.66	-5.68	<0.0001	0.0008
222900_PM_at	NRIP3	nuclear receptor interacting protein 3	-0.81	-5.68	<0.0001	0.0008
233121_PM_at		Homo sapiens cDNA FLJ12299 fis, clone MAMMA1001851	-1.03	-5.69	<0.0001	0.0008
211434_PM_s_at	CCRL2	chemokine (C-C motif) receptor-like 2	-1.01	-5.69	<0.0001	0.0008
226397_PM_s_at		Homo sapiens cDNA: FLJ21028 fis, clone CAE07155	-1.05	-5.78	<0.0001	0.0007

Probe Set ID	Gene Name	Gene Description	Log_2 FC	t-Statistic	P	FDR
203395_PM_s_at	HES1	hairy and enhancer of split 1, (Drosophila)	-0.77	-5.79	<0.0001	0.0006
226982_PM_at	ELL2	elongation factor, RNA polymerase II, 2	-0.88	-5.81	<0.0001	0.0006
217999_PM_s_at	PHLDA1	pleckstrin homology-like domain, family A, member 1	-1.06	-5.87	<0.0001	0.0006
212722_PM_s_at	JMJD6	jumonji domain containing 6	-0.69	-5.87	<0.0001	0.0006
206173_PM_x_at	GABPB1	GA binding protein transcription factor, beta subunit 1	-0.69	-5.91	<0.0001	0.0005
224978_PM_s_at	USP36	ubiquitin specific peptidase 36	-0.78	-5.92	<0.0001	0.0005
213638_PM_at	PHACTR1	phosphatase and actin regulator 1	-1.23	-5.92	<0.0001	0.0005
239876_PM_at			-1.01	-5.94	<0.0001	0.0005
238756_PM_at	GAS2L3	growth arrest-specific 2 like 3	-0.83	-5.97	<0.0001	0.0005
223915_PM_at	BCOR	BCL6 corepressor	-0.67	-5.99	<0.0001	0.0005
217998_PM_at	PHLDA1	pleckstrin homology-like domain, family A, member 1	-1.02	-6.01	<0.0001	0.0004
225842_PM_at	PHLDA1	pleckstrin homology-like domain, family A, member 1	-1.18	-6.02	<0.0001	0.0004
237204_PM_at		ESTs, Moderately similar to OVCA1	-0.75	-6.05	<0.0001	0.0004
1565868_PM_at	CD44	CD44 molecule (Indian blood group)	-0.76	-6.05	<0.0001	0.0004
207826_PM_s_at	ID3	inhibitor of DNA binding 3, dominant negative helix-loop-helix protein	-0.66	-6.07	<0.0001	0.0004
243829_PM_at	BRAF	v-raf murine sarcoma viral oncogene homolog B1	-0.61	-6.08	<0.0001	0.0004
201490_PM_s_at	PPIF	peptidylprolyl isomerase F	-0.82	-6.08	<0.0001	0.0004
241970_PM_at			-0.59	-6.11	<0.0001	0.0004
213452_PM_at	ZNF184	zinc finger protein 184	-0.73	-6.13	<0.0001	0.0004
216350_PM_s_at	ZNF10	zinc finger protein 10	-0.68	-6.15	<0.0001	0.0004
201712_PM_s_at	RANBP2	RAN binding protein 2	-0.66	-6.16	<0.0001	0.0004
228749_PM_at	ZDBF2	zinc finger, DBF-type containing 2	-0.66	-6.16	<0.0001	0.0004
237718_PM_at	EIF4E	eukaryotic translation initiation factor 4E	-1.21	-6.22	<0.0001	0.0003
205330_PM_at	MN1	meningioma (disrupted in balanced translocation) 1	-1.04	-6.24	<0.0001	0.0003
203411_PM_s_at	LMNA	lamin A/C	-0.59	-6.29	<0.0001	0.0003
210004_PM_at	OLR1	oxidized low density lipoprotein (lectin-like) receptor 1	-1.64	-6.31	<0.0001	0.0003
220370_PM_s_at	USP36	ubiquitin specific peptidase 36	-0.77	-6.36	<0.0001	0.0003
204790_PM_at	SMAD7	SMAD family member 7	-0.80	-6.37	<0.0001	0.0003
223484_PM_at	C15orf48	chromosome 15 open reading frame 48	-1.60	-6.42	<0.0001	0.0003
209803_PM_s_at	PHLDA2	pleckstrin homology-like domain, family A, member 2	-1.12	-6.44	<0.0001	0.0003
232406_PM_at		Homo sapiens cDNA FLJ13731 fis, clone PLACE3000142	-0.97	-6.47	<0.0001	0.0003
209270_PM_at	LAMB3	laminin, beta 3	-1.15	-6.51	<0.0001	0.0003
205013_PM_s_at	ADORA2A, SPECC1L-ADORA2A	adenosine A2a receptor,SPECC1L-ADORA2A readthrough	-0.75	-6.53	<0.0001	0.0003
1557049_PM_at	BTBD19	BTB (POZ) domain containing 19	-0.92	-6.54	<0.0001	0.0003
231779_PM_at	IRAK2	interleukin-1 receptor-associated kinase 2	-0.70	-6.55	<0.0001	0.0003
209959_PM_at	NR4A3	nuclear receptor subfamily 4, group A, member 3	-2.10	-6.61	<0.0001	0.0003
236402_PM_at	BRAF	v-raf murine sarcoma viral oncogene homolog B1	-0.71	-6.68	<0.0001	0.0003

Probe Set ID	Gene Name	Gene Description	Log_2 FC	t-Statistic	P	FDR
219557_PM_s_at	NRIP3	nuclear receptor interacting protein 3	-1.37	-6.68	<0.0001	0.0003
1553785_PM_at	RASGEF1B	RasGEF domain family, member 1B	-1.32	-6.81	<0.0001	0.0002
223394_PM_at	SERTAD1	SERTA domain containing 1	-0.80	-6.82	<0.0001	0.0002
226099_PM_at	ELL2	elongation factor, RNA polymerase II, 2	-0.97	-6.98	<0.0001	0.0002
209324_PM_s_at	RGS16	regulator of G-protein signaling 16	-0.97	-7.02	<0.0001	0.0002
227029_PM_at	FAM177A1	family with sequence similarity 177, member A1	-0.73	-7.21	<0.0001	0.0001
207978_PM_s_at	NR4A3	nuclear receptor subfamily 4, group A, member 3	-2.20	-7.27	<0.0001	0.0001
209383_PM_at	DDIT3	DNA-damage-inducible transcript 3	-1.09	-8.07	<0.0001	<0.0001
239451_PM_at			-1.46	-8.61	<0.0001	<0.0001

Table 76: Differentially expressed genes (Combined Study Groups, Day 0 vs. 7). Sorted by descending t-statistic. Probe set annotations were obtained from Affymetrix (HT HG-U133 Plus PM Annotations, Release 34,10/24/13).

Probe Set ID	Gene Name	Gene Description	Log_2 FC	t-Statistic	P	FDR
235286_PM_at			0.74	7.87	<0.0001	<0.0001
229228_PM_at	CREB5, LOC401317	cAMP responsive element binding protein 5,uncharacterized LOC401317	0.83	7.63	<0.0001	<0.0001
242943_PM_at	ST8SIA4	ST8 alpha-N-acetyl-neuraminide alpha-2,8-sialyltransferase 4	0.69	7.29	<0.0001	<0.0001
230036_PM_at	SAMD9L	sterile alpha motif domain containing 9-like	0.82	7.23	<0.0001	<0.0001
223220_PM_s_at	PARP9	poly (ADP-ribose) polymerase family, member 9	0.61	7.02	<0.0001	<0.0001
210166_PM_at	TLR5	toll-like receptor 5	0.91	6.78	<0.0001	0.0001
239108_PM_at	FAR2	fatty acyl CoA reductase 2	0.60	6.76	<0.0001	0.0001
225509_PM_at	SAP30L	SAP30-like	0.70	6.60	<0.0001	0.0001
1566501_PM_at		Homo sapiens cDNA FLJ20787 fis, clone COL02178.	0.78	6.56	<0.0001	0.0002
232382_PM_s_at	PCMTD1	protein-L-isoaspartate (D-aspartate) O-methyltransferase domain containing 1	0.59	6.51	<0.0001	0.0002
226603_PM_at	SAMD9L	sterile alpha motif domain containing 9-like	0.91	6.48	<0.0001	0.0002
1552334_PM_at	TRIOBP	TRIO and F-actin binding protein	0.61	6.46	<0.0001	0.0002
227618_PM_at			0.83	6.45	<0.0001	0.0002
206513_PM_at	AIM2	absent in melanoma 2	0.83	6.39	<0.0001	0.0002
213830_PM_at	YME1L1	YME1-like 1 ATPase	0.73	6.37	<0.0001	0.0002
217143_PM_s_at	TRDC, TRDC	T cell receptor delta constant	0.66	6.34	<0.0001	0.0002
202687_PM_s_at	TNFSF10	tumor necrosis factor (ligand) superfamily, member 10	0.67	6.32	<0.0001	0.0002
218943_PM_s_at	DDX58	DEAD (Asp-Glu-Ala-Asp) box polypeptide 58	0.62	6.24	<0.0001	0.0002
226853_PM_at	BMP2K	BMP2 inducible kinase	0.62	6.24	<0.0001	0.0002
230261_PM_at	ST8SIA4	ST8 alpha-N-acetyl-neuraminide alpha-2,8-sialyltransferase 4	0.66	6.23	<0.0001	0.0002
232829_PM_at	OR52K3P	olfactory receptor, family 52, subfamily K, member 3 pseudogene	0.64	6.18	<0.0001	0.0002
230860_PM_at	CEP19	centrosomal protein 19kDa	0.64	6.17	<0.0001	0.0002
225669_PM_at	IFNAR1	interferon (alpha, beta and omega) receptor 1	0.69	6.11	<0.0001	0.0003
232383_PM_at	TFEC	transcription factor EC	1.03	6.08	<0.0001	0.0003

Probe Set ID	Gene Name	Gene Description	Log_2 FC	t-Statistic	P	FDR
216191_PM_s_at	YME1L1	YME1-like 1 ATPase	0.64	6.05	<0.0001	0.0003
222981_PM_s_at	RAB10	RAB10, member RAS oncogene family	0.59	5.99	<0.0001	0.0003
202688_PM_at	TNFSF10	tumor necrosis factor (ligand) superfamily, member 10	0.72	5.97	<0.0001	0.0003
230836_PM_at	ST8SIA4	ST8 alpha-N-acetyl-neuraminide alpha-2,8-sialyltransferase 4	0.70	5.94	<0.0001	0.0003
1554948_PM_at		Homo sapiens, Similar to hypothetical protein PRO2852, clone MGC:23787 IMAGE:4249212, mRNA, complete cds.	0.72	5.93	<0.0001	0.0003
240061_PM_at			0.75	5.90	<0.0001	0.0003
229510_PM_at	MS4A14	membrane-spanning 4-domains, subfamily A, member 14	0.72	5.90	<0.0001	0.0003
236995_PM_x_at	TFEC	transcription factor EC	0.64	5.88	<0.0001	0.0004
203153_PM_at	IFIT1	interferon-induced protein with tetratricopeptide repeats 1	0.87	5.87	<0.0001	0.0004
243736_PM_at			0.63	5.82	<0.0001	0.0004
204007_PM_at	FCGR3B	Fc fragment of IgG, low affinity IIIb, receptor (CD16b)	0.79	5.82	<0.0001	0.0004
64883_PM_at	MOSPD2	motile sperm domain containing 2	0.62	5.79	<0.0001	0.0004
208304_PM_at	CCR3	chemokine (C-C motif) receptor 3	0.62	5.79	<0.0001	0.0004
1562031_PM_at	JAK2	Janus kinase 2	0.63	5.75	<0.0001	0.0005
203128_PM_at	SPTLC2	serine palmitoyltransferase, long chain base subunit 2	0.59	5.73	<0.0001	0.0005
222793_PM_at	DDX58	DEAD (Asp-Glu-Ala-Asp) box polypeptide 58	0.65	5.70	<0.0001	0.0005
221895_PM_at	MOSPD2	motile sperm domain containing 2	0.75	5.67	<0.0001	0.0005
210556_PM_at	NFATC3	nuclear factor of activated T-cells, cytoplasmic, calcineurin-dependent 3	0.67	5.64	<0.0001	0.0005
224358_PM_s_at	MS4A7	membrane-spanning 4-domains, subfamily A, member 7	0.72	5.61	<0.0001	0.0005
229450_PM_at	IFIT3	interferon-induced protein with tetratricopeptide repeats 3	0.80	5.57	<0.0001	0.0006
201193_PM_at	IDH1	isocitrate dehydrogenase 1 (NADP+), soluble	0.63	5.57	<0.0001	0.0006
229670_PM_at			0.72	5.56	<0.0001	0.0006
217104_PM_at	ST20	suppressor of tumorigenicity 20	0.64	5.53	<0.0001	0.0006
1568592_PM_at	TRIM69	tripartite motif containing 69	0.63	5.51	<0.0001	0.0006
211138_PM_s_at	KMO	kynurenine 3-monooxygenase (kynurenine 3-hydroxylase)	0.61	5.49	<0.0001	0.0006
225290_PM_at	ETNK1	ethanolamine kinase 1	0.62	5.45	<0.0001	0.0007
214329_PM_x_at	TNFSF10	tumor necrosis factor (ligand) superfamily, member 10	0.94	5.45	<0.0001	0.0007
225095_PM_at	SPTLC2	serine palmitoyltransferase, long chain base subunit 2	0.59	5.45	<0.0001	0.0007
243954_PM_at	LINC00877	long intergenic non-protein coding RNA 877	0.62	5.43	<0.0001	0.0007
230098_PM_at	PHF20L1	PHD finger protein 20-like 1	0.63	5.42	<0.0001	0.0007
226022_PM_at	SASH1	SAM and SH3 domain containing 1	0.62	5.42	<0.0001	0.0007
212632_PM_at	STX7	syntaxin 7	0.67	5.41	<0.0001	0.0007
206207_PM_at	CLC	Charcot-Leyden crystal galectin	0.72	5.41	<0.0001	0.0007
1552386_PM_at	GAPT	GRB2-binding adaptor protein, transmembrane	0.76	5.41	<0.0001	0.0007
1563088_PM_a_at	LOC284837	uncharacterized LOC284837	0.74	5.40	<0.0001	0.0007
227038_PM_at	SGMS2	sphingomyelin synthase 2	0.75	5.35	<0.0001	0.0008
224009_PM_x_at	DHRS9	dehydrogenase/reductase (SDR family) member 9	0.71	5.34	<0.0001	0.0008
226757_PM_at	IFIT2	interferon-induced protein with tetratricopeptide repeats 2	0.61	5.33	<0.0001	0.0008

Probe Set ID	Gene Name	Gene Description	Log_2 FC	t-Statistic	P	FDR
209200_PM_at	MEF2C	myocyte enhancer factor 2C	0.66	5.29	<0.0001	0.0009
242598_PM_at			0.96	5.28	<0.0001	0.0009
222388_PM_s_at	VPS35	vacuolar protein sorting 35 homolog (S. cerevisiae)	0.60	5.28	<0.0001	0.0009
201798_PM_s_at	MYOF	myoferlin	0.68	5.28	<0.0001	0.0009
218230_PM_at	ARFIP1	ADP-ribosylation factor interacting protein 1	0.81	5.27	<0.0001	0.0009
219093_PM_at	PID1	phosphotyrosine interaction domain containing 1	0.60	5.26	<0.0001	0.0009
223155_PM_at	HDHD2	haloacid dehalogenase-like hydrolase domain containing 2	0.60	5.25	<0.0001	0.0009
226423_PM_at	PAQR8	progestin and adipoQ receptor family member VIII	0.82	5.22	<0.0001	0.001
223583_PM_at	TNFAIP8L2	tumor necrosis factor, alpha-induced protein 8-like 2	0.70	5.20	<0.0001	0.001
209960_PM_at	HGF	hepatocyte growth factor (hepapoietin A; scatter factor)	0.64	5.17	<0.0001	0.001
1566557_PM_at	BAIAP2-AS1	BAIAP2 antisense RNA 1 (head to head)	0.63	5.17	<0.0001	0.0011
227626_PM_at	PAQR8	progestin and adipoQ receptor family member VIII	0.78	5.15	<0.0001	0.0011
218303_PM_x_at	KRCC1	lysine-rich coiled-coil 1	0.61	5.10	<0.0001	0.0012
235625_PM_at	VPS41	vacuolar protein sorting 41 homolog (S. cerevisiae)	0.69	5.09	<0.0001	0.0012
213238_PM_at	ATP10D	ATPase, class V, type 10D	0.76	5.07	<0.0001	0.0013
232724_PM_at	MS4A6A	membrane-spanning 4-domains, subfamily A, member 6A	0.60	5.07	<0.0001	0.0013
224962_PM_at	C9orf69	chromosome 9 open reading frame 69	0.72	5.05	<0.0001	0.0013
219243_PM_at	GIMAP4	GTPase, IMAP family member 4	0.69	5.03	<0.0001	0.0014
213626_PM_at	CBR4	carbonyl reductase 4	0.66	5.01	<0.0001	0.0014
226047_PM_at	MRVI1	murine retrovirus integration site 1 homolog	0.66	5.00	<0.0001	0.0015
200799_PM_at	HSPA1A, HSPA1B	heat shock 70kDa protein 1A,heat shock 70kDa protein 1B	0.77	4.99	<0.0001	0.0015
1562289_PM_at		Homo sapiens mRNA; cDNA DKFZp434N0220 (from clone DKFZp434N0220)	0.63	4.97	<0.0001	0.0016
238992_PM_at	POLI	polymerase (DNA directed) iota	0.59	4.94	<0.0001	0.0017
227052_PM_at			0.64	4.94	<0.0001	0.0017
230918_PM_at			0.72	4.93	<0.0001	0.0017
219607_PM_s_at	MS4A4A	membrane-spanning 4-domains, subfamily A, member 4A	0.63	4.92	<0.0001	0.0017
202451_PM_at	GTF2H1	general transcription factor IIH, polypeptide 1, 62kDa	0.60	4.92	<0.0001	0.0017
204006_PM_s_at	FCGR3A, FCGR3B	Fc fragment of IgG, low affinity IIIa, receptor (CD16a),Fc fragment of IgG, low affinity IIIb, receptor (CD16b)	0.76	4.91	<0.0001	0.0017
205552_PM_s_at	OAS1	2'-5'-oligoadenylate synthetase 1, 40/46kDa	0.69	4.91	<0.0001	0.0018
244298_PM_at			0.59	4.90	<0.0001	0.0018
222691_PM_at	SLC35B3	solute carrier family 35, member B3	0.65	4.87	<0.0001	0.0018
1552553_PM_a_at	NLRC4	NLR family, CARD domain containing 4	0.65	4.85	<0.0001	0.0019
229007_PM_at	LOC283788	FSHD region gene 1 pseudogene	0.60	4.85	<0.0001	0.0019
205898_PM_at	CX3CR1	chemokine (C-X3-C motif) receptor 1	1.04	4.83	<0.0001	0.002
225989_PM_at	HERC4	HECT and RLD domain containing E3 ubiquitin protein ligase 4	0.60	4.78	<0.0001	0.0022
1555037_PM_a_at	IDH1	isocitrate dehydrogenase 1 (NADP+), soluble	0.67	4.77	<0.0001	0.0022
207008_PM_at	CXCR2	chemokine (C-X-C motif) receptor 2	0.68	4.77	<0.0001	0.0022

Probe Set ID	Gene Name	Gene Description	Log_2 FC	t-Statistic	P	FDR
1556185_PM_a_at	CTB-167B5.2, OTTHUMG00000179513	NULL	0.74	4.76	<0.0001	0.0022
220146_PM_at	TLR7	toll-like receptor 7	0.62	4.76	<0.0001	0.0022
243824_PM_at	LOC100996870	uncharacterized LOC100996870	0.81	4.76	<0.0001	0.0022
204972_PM_at	OAS2	2'-5'-oligoadenylate synthetase 2, 69/71kDa	0.63	4.70	<0.0001	0.0024
228280_PM_at	ZC3HAV1L	zinc finger CCCH-type, antiviral 1-like	0.59	4.69	<0.0001	0.0025
232958_PM_at		Homo sapiens cDNA FLJ13595 fis, clone PLACE1009595	0.67	4.67	<0.0001	0.0026
228190_PM_at	ATG4C	autophagy related 4C, cysteine peptidase	0.67	4.67	<0.0001	0.0026
227426_PM_at	SOS1	son of sevenless homolog 1 (Drosophila)	0.66	4.65	<0.0001	0.0027
235443_PM_at	LOC100131067	uncharacterized LOC100131067	0.61	4.60	<0.0001	0.003
213653_PM_at	METTL3	methyltransferase like 3	0.60	4.59	<0.0001	0.003
236583_PM_at	GIMAP1	GTPase, IMAP family member 1	0.64	4.48	<0.0001	0.0038
224989_PM_at		Homo sapiens cDNA FLJ13289 fis, clone OVARC1001170	0.69	4.47	<0.0001	0.0039
232000_PM_at		Homo sapiens cDNA FLJ11508 fis, clone HEMBA1002162	0.66	4.44	<0.0001	0.0041
211163_PM_s_at	TNFRSF10C	tumor necrosis factor receptor superfamily, member 10c, decoy without an intracellular domain	0.65	4.42	<0.0001	0.0043
216984_PM_x_at	IGLJ2, IGLJ2, IGLJ3, IGLJ3, IGLV2-14, IGLV2-14	immunoglobulin lambda joining 2,immunoglobulin lambda joining 3,immunoglobulin lambda variable 2-14	0.63	4.37	<0.0001	0.0045
202869_PM_at	OAS1	2'-5'-oligoadenylate synthetase 1, 40/46kDa	0.68	4.33	0.0001	0.0049
216050_PM_at		Homo sapiens cDNA: FLJ20931 fis, clone ADSE01282	0.75	4.26	0.0001	0.0057
235643_PM_at	SAMD9L	sterile alpha motif domain containing 9-like	0.59	4.09	0.0002	0.0081
235306_PM_at	GIMAP8	GTPase, IMAP family member 8	0.60	4.06	0.0002	0.0086
231093_PM_at	FCRL3	Fc receptor-like 3	0.59	4.05	0.0002	0.0087
206978_PM_at	CCR2	chemokine (C-C motif) receptor 2	0.77	3.94	0.0003	0.0107
206834_PM_at	HBD	hemoglobin, delta	0.65	3.85	0.0004	0.0128
231956_PM_at	RNF213	ring finger protein 213	0.62	3.83	0.0005	0.0132
209995_PM_s_at	TCL1A	T-cell leukemia/lymphoma 1A	0.60	3.78	0.0005	0.0148
225207_PM_at	PDK4	pyruvate dehydrogenase kinase, isozyme 4	0.86	3.75	0.0006	0.0155
217148_PM_x_at	IGLV2-14, IGLV2-14	immunoglobulin lambda variable 2-14	0.63	3.70	0.0007	0.0169
205592_PM_at	SLC4A1	solute carrier family 4, anion exchanger, member 1 (erythrocyte membrane protein band 3, Diego blood group)	0.72	3.66	0.0008	0.0184
238653_PM_at			0.59	3.59	0.0009	0.0211
205950_PM_s_at	CA1	carbonic anhydrase I	0.72	3.14	0.0033	0.0488
205067_PM_at	IL1B	interleukin 1, beta	-1.06	-3.21	0.0027	0.0428
235086_PM_at	THBS1	thrombospondin 1	-0.99	-3.21	0.0027	0.0426
237496_PM_at		ESTs, Weakly similar to ALU7_HUMAN ALU SUBFAMILY SQ SEQUENCE CONTAMINATION WARNING ENTRY (H.sapiens)	-0.75	-3.22	0.0026	0.0418

Probe Set ID	Gene Name	Gene Description	Log_2 FC	t-Statistic	P	FDR
39402_PM_at	IL1B	interleukin 1, beta	-1.02	-3.24	0.0025	0.0398
1569477_PM_at		Homo sapiens, clone IMAGE:4291396, mRNA	-0.60	-3.25	0.0024	0.0396
230380_PM_at	THAP2	THAP domain containing, apoptosis associated protein 2	-0.63	-3.25	0.0024	0.0396
207075_PM_at	NLRP3	NLR family, pyrin domain containing 3	-0.62	-3.25	0.0024	0.0391
244840_PM_x_at	DOCK4	dedicator of cytokinesis 4	-0.76	-3.31	0.002	0.0354
203504_PM_s_at	ABCA1	ATP-binding cassette, sub-family A (ABC1), member 1	-0.66	-3.32	0.002	0.0348
209774_PM_x_at	CXCL2	chemokine (C-X-C motif) ligand 2	-0.93	-3.32	0.002	0.0347
205220_PM_at	HCAR3	hydroxycarboxylic acid receptor 3	-0.73	-3.37	0.0017	0.0318
240038_PM_at			-1.02	-3.40	0.0016	0.0298
201466_PM_s_at	JUN	jun proto-oncogene	-0.59	-3.49	0.0012	0.0256
218856_PM_at	TNFRSF21	tumor necrosis factor receptor superfamily, member 21	-0.64	-3.51	0.0012	0.0246
215630_PM_at		Homo sapiens cDNA FLJ14102 fis, clone MAMMA1000940	-0.74	-3.52	0.0011	0.0239
201340_PM_s_at	ENC1	ectodermal-neural cortex 1 (with BTB domain)	-0.60	-3.53	0.0011	0.0238
241740_PM_at		ESTs, Moderately similar to ALU7_HUMAN ALU SUBFAMILY SQ SEQUENCE CONTAMINATION WARNING ENTRY (H.sapiens)	-0.71	-3.53	0.0011	0.0237
1564430_PM_at		Homo sapiens, clone IMAGE:2960704, mRNA	-0.63	-3.54	0.0011	0.0234
233674_PM_at		Homo sapiens cDNA: FLJ22633 fis, clone HSI06502	-0.61	-3.55	0.0011	0.0229
233127_PM_at		Homo sapiens cDNA FLJ12412 fis, clone MAMMA1003004	-0.68	-3.65	0.0008	0.0187
238756_PM_at	GAS2L3	growth arrest-specific 2 like 3	-0.71	-3.66	0.0008	0.0184
225955_PM_at	METRNL	meteorin, glial cell differentiation regulator-like	-0.63	-3.68	0.0007	0.0175
212158_PM_at	SDC2	syndecan 2	-0.86	-3.71	0.0007	0.0168
1554980_PM_a_at	ATF3	activating transcription factor 3	-0.76	-3.73	0.0006	0.0162
239827_PM_at	RGCC	regulator of cell cycle	-0.80	-3.77	0.0006	0.0149
1557166_PM_at	PDCD4	programmed cell death 4 (neoplastic transformation inhibitor)	-0.73	-3.78	0.0005	0.0148
37028_PM_at	PPP1R15A	protein phosphatase 1, regulatory subunit 15A	-0.67	-3.80	0.0005	0.0142
240024_PM_at	SEC14L2	SEC14-like 2 (S. cerevisiae)	-0.60	-3.80	0.0005	0.014
218880_PM_at	FOSL2	FOS-like antigen 2	-0.80	-3.85	0.0004	0.0129
235592_PM_at			-1.00	-3.88	0.0004	0.0121
224836_PM_at	TP53INP2	tumor protein p53 inducible nuclear protein 2	-0.72	-3.90	0.0004	0.0116
202637_PM_s_at	ICAM1	intercellular adhesion molecule 1	-0.76	-3.91	0.0004	0.0116
1561882_PM_at	SYTL3	synaptotagmin-like 3	-0.61	-3.91	0.0004	0.0114
202988_PM_s_at	RGS1	regulator of G-protein signaling 1	-0.81	-3.92	0.0004	0.0111
202638_PM_s_at	ICAM1	intercellular adhesion molecule 1	-0.76	-3.94	0.0003	0.0108
203505_PM_at	ABCA1	ATP-binding cassette, sub-family A (ABC1), member 1	-0.63	-3.94	0.0003	0.0107
207113_PM_s_at	TNF	tumor necrosis factor	-0.73	-3.95	0.0003	0.0107
1564093_PM_at	NEK1	NIMA-related kinase 1	-0.78	-3.96	0.0003	0.0105
208869_PM_s_at	GABARAPL1	GABA(A) receptor-associated protein like 1	-0.62	-3.97	0.0003	0.0102
1554836_PM_at	USP36	ubiquitin specific peptidase 36	-0.60	-3.97	0.0003	0.0102
231972_PM_at		Homo sapiens cDNA: FLJ21028 fis, clone CAE07155	-0.76	-4.00	0.0003	0.0097

Probe Set ID	Gene Name	Gene Description	Log_2 FC	t-Statistic	P	FDR
202437_PM_s_at	CYP1B1	cytochrome P450, family 1, subfamily B, polypeptide 1	-0.72	-4.00	0.0003	0.0096
210258_PM_at	RGS13	regulator of G-protein signaling 13	-0.59	-4.01	0.0003	0.0095
212659_PM_s_at	IL1RN	interleukin 1 receptor antagonist	-0.62	-4.02	0.0003	0.0093
201195_PM_s_at	SLC7A5	solute carrier family 7 (amino acid transporter light chain, L system), member 5	-0.80	-4.05	0.0002	0.0088
231484_PM_at			-0.60	-4.07	0.0002	0.0084
219312_PM_s_at	ZBTB10	zinc finger and BTB domain containing 10	-0.77	-4.08	0.0002	0.0083
226034_PM_at	DUSP4	dual specificity phosphatase 4	-0.82	-4.11	0.0002	0.0077
204363_PM_at	F3	coagulation factor III (thromboplastin, tissue factor)	-0.62	-4.11	0.0002	0.0077
216243_PM_s_at	IL1RN	interleukin 1 receptor antagonist	-0.60	-4.11	0.0002	0.0077
226397_PM_s_at		Homo sapiens cDNA: FLJ21028 fis, clone CAE07155	-0.89	-4.19	0.0002	0.0067
232406_PM_at		Homo sapiens cDNA FLJ13731 fis, clone PLACE3000142	-0.81	-4.20	0.0002	0.0065
1565776_PM_at		Homo sapiens mRNA; cDNA DKFZp667M067 (from clone DKFZp667M067)	-0.75	-4.20	0.0002	0.0064
1562255_PM_at	SYTL3	synaptotagmin-like 3	-0.78	-4.23	0.0001	0.0061
36711_PM_at	MAFF	v-maf musculoaponeurotic fibrosarcoma oncogene homolog F (avian)	-1.02	-4.23	0.0001	0.006
244578_PM_at	LCP2	lymphocyte cytosolic protein 2 (SH2 domain containing leukocyte protein of 76kDa)	-0.62	-4.24	0.0001	0.0059
204103_PM_at	CCL4	chemokine (C-C motif) ligand 4	-0.87	-4.25	0.0001	0.0058
233458_PM_at	LOC101060521, POLR3E	DNA-directed RNA polymerase III subunit RPC5-like,polymerase (RNA) III (DNA directed) polypeptide E (80kD)	-0.62	-4.26	0.0001	0.0056
202435_PM_s_at	CYP1B1	cytochrome P450, family 1, subfamily B, polypeptide 1	-0.71	-4.29	0.0001	0.0054
209967_PM_s_at	CREM	cAMP responsive element modulator	-1.05	-4.30	0.0001	0.0053
202436_PM_s_at	CYP1B1	cytochrome P450, family 1, subfamily B, polypeptide 1	-0.70	-4.30	0.0001	0.0052
240207_PM_at			-0.65	-4.32	0.0001	0.005
204014_PM_at	DUSP4	dual specificity phosphatase 4	-0.66	-4.33	0.0001	0.0049
233309_PM_at		Homo sapiens cDNA FLJ11759 fis, clone HEMBA1005616	-0.66	-4.34	0.0001	0.0049
205193_PM_at	MAFF	v-maf musculoaponeurotic fibrosarcoma oncogene homolog F (avian)	-1.02	-4.36	<0.0001	0.0047
229715_PM_at	OTTHUMG00000179015, RP1-239B22.5	NULL	-0.61	-4.36	<0.0001	0.0046
1554096_PM_a_at	RBM33	RNA binding motif protein 33	-0.61	-4.37	<0.0001	0.0045
206173_PM_x_at	GABPB1	GA binding protein transcription factor, beta subunit 1	-0.65	-4.38	<0.0001	0.0045
243659_PM_at			-0.98	-4.38	<0.0001	0.0045
202014_PM_at	PPP1R15A	protein phosphatase 1, regulatory subunit 15A	-0.77	-4.39	<0.0001	0.0045
202068_PM_s_at	LDLR	low density lipoprotein receptor	-0.65	-4.39	<0.0001	0.0044
1554906_PM_a_at	MPHOSPH6	M-phase phosphoprotein 6	-0.62	-4.39	<0.0001	0.0044
203394_PM_s_at	HES1	hairy and enhancer of split 1, (Drosophila)	-0.77	-4.40	<0.0001	0.0044
1555638_PM_a_at	SAMSN1	SAM domain, SH3 domain and nuclear localization signals 1	-0.89	-4.40	<0.0001	0.0043
217127_PM_at	CTH	cystathionase (cystathionine gamma-lyase)	-0.78	-4.41	<0.0001	0.0043
202933_PM_s_at	YES1	v-yes-1 Yamaguchi sarcoma viral oncogene homolog 1	-0.65	-4.41	<0.0001	0.0043
214230_PM_at	CDC42	cell division cycle 42	-0.59	-4.43	<0.0001	0.0042
215485_PM_s_at	ICAM1	intercellular adhesion molecule 1	-0.60	-4.44	<0.0001	0.0041

Probe Set ID	Gene Name	Gene Description	Log_2 FC	t-Statistic	P	FDR
201631_PM_s_at	IER3	immediate early response 3	-0.86	-4.45	<0.0001	0.004
204790_PM_at	SMAD7	SMAD family member 7	-0.71	-4.46	<0.0001	0.0039
237204_PM_at		ESTs, Moderately similar to OVCA1	-0.69	-4.46	<0.0001	0.0039
216350_PM_s_at	ZNF10	zinc finger protein 10	-0.64	-4.48	<0.0001	0.0038
206983_PM_at	CCR6	chemokine (C-C motif) receptor 6	-0.63	-4.49	<0.0001	0.0037
1570432_PM_at		Homo sapiens clone pp10199 unknown mRNA.	-0.67	-4.49	<0.0001	0.0037
230233_PM_at			-1.15	-4.50	<0.0001	0.0037
201465_PM_s_at	JUN	jun proto-oncogene	-0.86	-4.51	<0.0001	0.0036
1553266_PM_at	CNOT6L	CCR4-NOT transcription complex, subunit 6-like	-0.64	-4.51	<0.0001	0.0036
230511_PM_at	CREM	cAMP responsive element modulator	-1.12	-4.51	<0.0001	0.0036
227140_PM_at	INHBA	inhibin, beta A	-0.77	-4.53	<0.0001	0.0034
202932_PM_at	YES1	v-yes-1 Yamaguchi sarcoma viral oncogene homolog 1	-0.81	-4.55	<0.0001	0.0033
1564150_PM_a_at	C12orf79	chromosome 12 open reading frame 79	-0.96	-4.57	<0.0001	0.0031
202684_PM_s_at	RNMT	RNA (guanine-7-) methyltransferase	-0.66	-4.59	<0.0001	0.003
218631_PM_at	AVPI1	arginine vasopressin-induced 1	-0.73	-4.59	<0.0001	0.003
233952_PM_s_at	ZBTB21	zinc finger and BTB domain containing 21	-0.70	-4.62	<0.0001	0.0029
1560485_PM_at	HIVEP1	human immunodeficiency virus type I enhancer binding protein 1	-0.61	-4.62	<0.0001	0.0029
230652_PM_at	ARAF	v-raf murine sarcoma 3611 viral oncogene homolog	-0.75	-4.63	<0.0001	0.0028
206157_PM_at	PTX3	pentraxin 3, long	-0.88	-4.64	<0.0001	0.0027
217996_PM_at	PHLDA1	pleckstrin homology-like domain, family A, member 1	-1.47	-4.66	<0.0001	0.0027
222180_PM_at	OTTHUMG00000178814, RP11-769O8.3	[illegible]	-0.69	-4.66	<0.0001	0.0027
209325_PM_s_at	RGS16	regulator of G-protein signaling 16	-0.60	-4.67	<0.0001	0.0026
219228_PM_at	ZNF331	zinc finger protein 331	-1.18	-4.69	<0.0001	0.0025
227099_PM_s_at	C11orf96	chromosome 11 open reading frame 96	-0.74	-4.70	<0.0001	0.0025
244054_PM_at			-0.92	-4.70	<0.0001	0.0025
207630_PM_s_at	CREM	cAMP responsive element modulator	-1.18	-4.71	<0.0001	0.0024
225539_PM_at	ZBTB21	zinc finger and BTB domain containing 21	-0.75	-4.71	<0.0001	0.0024
230218_PM_at	HIC1	hypermethylated in cancer 1	-0.61	-4.72	<0.0001	0.0024
212722_PM_s_at	JMJD6	jumonji domain containing 6	-0.63	-4.72	<0.0001	0.0024
223787_PM_s_at	CCDC174	coiled-coil domain containing 174	-0.59	-4.73	<0.0001	0.0024
226833_PM_at	CYB5D1	cytochrome b5 domain containing 1	-0.71	-4.73	<0.0001	0.0023
242058_PM_at			-0.61	-4.74	<0.0001	0.0023
233899_PM_x_at	ZBTB10	zinc finger and BTB domain containing 10	-0.82	-4.77	<0.0001	0.0022
204491_PM_at	PDE4D	phosphodiesterase 4D, cAMP-specific	-0.72	-4.77	<0.0001	0.0022
242727_PM_at	ARL5B	ADP-ribosylation factor-like 5B	-0.79	-4.77	<0.0001	0.0022
241970_PM_at			-0.61	-4.77	<0.0001	0.0022
213638_PM_at	PHACTR1	phosphatase and actin regulator 1	-1.09	-4.78	<0.0001	0.0022

Probe Set ID	Gene Name	Gene Description	Log_2 FC	t-Statistic	P	FDR
220612_PM_at		Homo sapiens PRO0641 protein (PRO0641), mRNA.	-0.66	-4.78	<0.0001	0.0022
214508_PM_x_at	CREM	cAMP responsive element modulator	-1.04	-4.80	<0.0001	0.0021
223767_PM_at	GPR84	G protein-coupled receptor 84	-0.74	-4.85	<0.0001	0.0019
209803_PM_s_at	PHLDA2	pleckstrin homology-like domain, family A, member 2	-1.01	-4.88	<0.0001	0.0018
223915_PM_at	BCOR	BCL6 corepressor	-0.59	-4.93	<0.0001	0.0017
237718_PM_at	EIF4E	eukaryotic translation initiation factor 4E	-1.14	-4.94	<0.0001	0.0017
1553785_PM_at	RASGEF1B	RasGEF domain family, member 1B	-1.15	-4.95	<0.0001	0.0016
240103_PM_at	LOC100996457	uncharacterized LOC100996457	-1.22	-4.95	<0.0001	0.0016
243771_PM_at			-0.82	-4.97	<0.0001	0.0016
210837_PM_s_at	PDE4D	phosphodiesterase 4D, cAMP-specific	-0.96	-5.00	<0.0001	0.0015
215072_PM_x_at	C10orf137	chromosome 10 open reading frame 137	-0.66	-5.00	<0.0001	0.0015
206683_PM_at	ZNF165	zinc finger protein 165	-0.60	-5.01	<0.0001	0.0014
201712_PM_s_at	RANBP2	RAN binding protein 2	-0.63	-5.02	<0.0001	0.0014
1554095_PM_at	RBM33	RNA binding motif protein 33	-0.66	-5.06	<0.0001	0.0013
203395_PM_s_at	HES1	hairy and enhancer of split 1, (Drosophila)	-0.79	-5.07	<0.0001	0.0013
224098_PM_at		Homo sapiens PRO1546 mRNA, complete cds.	-0.61	-5.08	<0.0001	0.0013
236646_PM_at	TMEM52B	transmembrane protein 52B	-0.63	-5.09	<0.0001	0.0012
226982_PM_at	ELL2	elongation factor, RNA polymerase II, 2	-0.87	-5.13	<0.0001	0.0012
210004_PM_at	OLR1	oxidized low density lipoprotein (lectin-like) receptor 1	-1.56	-5.16	<0.0001	0.0011
211423_PM_s_at	SC5D	sterol-C5-desaturase	-0.73	-5.17	<0.0001	0.0011
226922_PM_at	RANBP2	RAN binding protein 2	-0.59	-5.18	<0.0001	0.001
213452_PM_at	ZNF184	zinc finger protein 184	-0.70	-5.18	<0.0001	0.001
228749_PM_at	ZDBF2	zinc finger, DBF-type containing 2	-0.65	-5.22	<0.0001	0.001
201489_PM_at	PPIF	peptidylprolyl isomerase F	-0.76	-5.22	<0.0001	0.001
233121_PM_at		Homo sapiens cDNA FLJ12299 fis, clone MAMMA1001851	-1.02	-5.27	<0.0001	0.0009
204567_PM_s_at	ABCG1	ATP-binding cassette, sub-family G (WHITE), member 1	-0.69	-5.28	<0.0001	0.0009
217999_PM_s_at	PHLDA1	pleckstrin homology-like domain, family A, member 1	-0.98	-5.32	<0.0001	0.0008
1557049_PM_at	BTBD19	BTB (POZ) domain containing 19	-0.85	-5.34	<0.0001	0.0008
227613_PM_at	ZNF331	zinc finger protein 331	-1.39	-5.37	<0.0001	0.0008
1552711_PM_a_at	CYB5D1	cytochrome b5 domain containing 1	-0.64	-5.37	<0.0001	0.0007
201490_PM_s_at	PPIF	peptidylprolyl isomerase F	-0.87	-5.41	<0.0001	0.0007
211434_PM_s_at	CCRL2	chemokine (C-C motif) receptor-like 2	-0.94	-5.43	<0.0001	0.0007
223394_PM_at	SERTAD1	SERTA domain containing 1	-0.80	-5.43	<0.0001	0.0007
217998_PM_at	PHLDA1	pleckstrin homology-like domain, family A, member 1	-1.02	-5.44	<0.0001	0.0007
204541_PM_at	SEC14L2	SEC14-like 2 (S. cerevisiae)	-0.59	-5.44	<0.0001	0.0007
238693_PM_at	PHC3	polyhomeotic homolog 3 (Drosophila)	-0.61	-5.46	<0.0001	0.0007
207826_PM_s_at	ID3	inhibitor of DNA binding 3, dominant negative helix-loop-helix protein	-0.67	-5.46	<0.0001	0.0007
231779_PM_at	IRAK2	interleukin-1 receptor-associated kinase 2	-0.64	-5.46	<0.0001	0.0007
218851_PM_s_at	SFT2D3, WDR33	SFT2 domain containing 3,WD repeat domain 33	-0.73	-5.46	<0.0001	0.0007

Probe Set ID	Gene Name	Gene Description	Log_2 FC	t-Statistic	P	FDR
210836_PM_x_at	PDE4D	phosphodiesterase 4D, cAMP-specific	-0.85	-5.49	<0.0001	0.0006
1556499_PM_s_at	COL1A1	collagen, type I, alpha 1	-0.83	-5.50	<0.0001	0.0006
216066_PM_at	ABCA1	ATP-binding cassette, sub-family A (ABC1), member 1	-0.75	-5.50	<0.0001	0.0006
204435_PM_at	NUPL1	nucleoporin like 1	-0.74	-5.52	<0.0001	0.0006
241985_PM_at	JMY	junction mediating and regulatory protein, p53 cofactor	-0.88	-5.52	<0.0001	0.0006
223484_PM_at	C15orf48	chromosome 15 open reading frame 48	-1.48	-5.53	<0.0001	0.0006
207850_PM_at	CXCL3	chemokine (C-X-C motif) ligand 3	-1.33	-5.54	<0.0001	0.0006
204614_PM_at	SERPINB2	serpin peptidase inhibitor, clade B (ovalbumin), member 2	-1.79	-5.54	<0.0001	0.0006
217997_PM_at	PHLDA1	pleckstrin homology-like domain, family A, member 1	-1.54	-5.58	<0.0001	0.0005
205479_PM_s_at	PLAU	plasminogen activator, urokinase	-0.74	-5.60	<0.0001	0.0005
209959_PM_at	NR4A3	nuclear receptor subfamily 4, group A, member 3	-2.16	-5.61	<0.0001	0.0005
227093_PM_at	USP36	ubiquitin specific peptidase 36	-0.73	-5.62	<0.0001	0.0005
226099_PM_at	ELL2	elongation factor, RNA polymerase II, 2	-0.98	-5.62	<0.0001	0.0005
219557_PM_s_at	NRIP3	nuclear receptor interacting protein 3	-1.29	-5.63	<0.0001	0.0005
228536_PM_at	PRMT10	protein arginine methyltransferase 10 (putative)	-0.76	-5.64	<0.0001	0.0005
223028_PM_s_at	SNX9	sorting nexin 9	-0.71	-5.65	<0.0001	0.0005
232081_PM_at	ABCG1	ATP-binding cassette, sub-family G (WHITE), member 1	-0.68	-5.67	<0.0001	0.0005
203835_PM_at	LRRC32	leucine rich repeat containing 32	-0.65	-5.67	<0.0001	0.0005
207978_PM_s_at	NR4A3	nuclear receptor subfamily 4, group A, member 3	-2.15	-5.70	<0.0001	0.0005
224978_PM_s_at	USP36	ubiquitin specific peptidase 36	-0.87	-5.71	<0.0001	0.0005
211840_PM_s_at	PDE4D	phosphodiesterase 4D, cAMP-specific	-0.78	-5.72	<0.0001	0.0005
208115_PM_x_at	C10orf137	chromosome 10 open reading frame 137	-0.67	-5.72	<0.0001	0.0005
239835_PM_at	KBTBD8	kelch repeat and BTB (POZ) domain containing 8	-0.76	-5.73	<0.0001	0.0005
204015_PM_s_at	DUSP4	dual specificity phosphatase 4	-0.82	-5.75	<0.0001	0.0005
1556676_PM_a_at	OTTHUMG00000175917, RP11-425D10.10	[illegible]	-0.78	-5.75	<0.0001	0.0005
1554290_PM_at	HERC3	HECT and RLD domain containing E3 ubiquitin protein ligase 3	-0.65	-5.77	<0.0001	0.0004
225842_PM_at	PHLDA1	pleckstrin homology-like domain, family A, member 1	-1.13	-5.78	<0.0001	0.0004
227029_PM_at	FAM177A1	family with sequence similarity 177, member A1	-0.75	-5.81	<0.0001	0.0004
1563505_PM_at	DUSP16	dual specificity phosphatase 16	-0.62	-5.82	<0.0001	0.0004
223887_PM_at	GPR132	G protein-coupled receptor 132	-0.81	-5.85	<0.0001	0.0004
244868_PM_at			-0.91	-5.95	<0.0001	0.0003
212750_PM_at	PPP1R16B	protein phosphatase 1, regulatory subunit 16B	-0.62	-5.98	<0.0001	0.0003
242040_PM_at	GCNT7	glucosaminyl (N-acetyl) transferase family member 7	-0.64	-6.00	<0.0001	0.0003
224979_PM_s_at	USP36	ubiquitin specific peptidase 36	-0.68	-6.03	<0.0001	0.0003
205476_PM_at	CCL20	chemokine (C-C motif) ligand 20	-2.03	-6.07	<0.0001	0.0003
212086_PM_x_at	LMNA	lamin A/C	-0.60	-6.08	<0.0001	0.0003
238645_PM_at			-0.66	-6.08	<0.0001	0.0003

Probe Set ID	Gene Name	Gene Description	Log_2 FC	t-Statistic	P	FDR
206648_PM_at	ZNF571	zinc finger protein 571	-1.00	-6.13	<0.0001	0.0002
203411_PM_s_at	LMNA	lamin A/C	-0.62	-6.14	<0.0001	0.0002
215501_PM_s_at	DUSP10	dual specificity phosphatase 10	-0.70	-6.18	<0.0001	0.0002
239567_PM_at			-0.90	-6.24	<0.0001	0.0002
222900_PM_at	NRIP3	nuclear receptor interacting protein 3	-0.87	-6.29	<0.0001	0.0002
204087_PM_s_at	SLC5A6	solute carrier family 5 (sodium-dependent vitamin transporter), member 6	-0.71	-6.32	<0.0001	0.0002
210118_PM_s_at	IL1A	interleukin 1, alpha	-1.98	-6.33	<0.0001	0.0002
220370_PM_s_at	USP36	ubiquitin specific peptidase 36	-0.82	-6.35	<0.0001	0.0002
205330_PM_at	MN1	meningioma (disrupted in balanced translocation) 1	-0.93	-6.38	<0.0001	0.0002
221563_PM_at	DUSP10	dual specificity phosphatase 10	-0.66	-6.40	<0.0001	0.0002
1565868_PM_at	CD44	CD44 molecule (Indian blood group)	-0.77	-6.49	<0.0001	0.0002
209383_PM_at	DDIT3	DNA-damage-inducible transcript 3	-1.04	-6.51	<0.0001	0.0002
239876_PM_at			-1.01	-6.52	<0.0001	0.0002
206374_PM_at	DUSP8	dual specificity phosphatase 8	-0.73	-6.71	<0.0001	0.0001
206404_PM_at	FGF9	fibroblast growth factor 9 (glia-activating factor)	-0.84	-6.83	<0.0001	0.0001
209270_PM_at	LAMB3	laminin, beta 3	-1.15	-6.94	<0.0001	<0.0001
1555279_PM_at	ARMC8	armadillo repeat containing 8	-0.68	-7.15	<0.0001	<0.0001
209324_PM_s_at	RGS16	regulator of G-protein signaling 16	-1.04	-7.41	<0.0001	<0.0001
239451_PM_at			-1.47	-8.34	<0.0001	<0.0001

Table 77: Differentially expressed genes (Combined Study Groups, Day 0 vs. 14). Sorted by descending t-statistic. Probe set annotations were obtained from Affymetrix (HT HG-U133 Plus PM Annotations, Release 34,10/24/13).

Gene Name	Gene Description	Log_2 FC	t-Statistic	P	FDR
PARP9	poly (ADP-ribose) polymerase family, member 9	0.69	6.80	<0.0001	0.0003
GBP1	guanylate binding protein 1, interferon-inducible	1.14	6.54	<0.0001	0.0003
OTTHUMG00000159342		0.79	6.52	<0.0001	0.0003
RP1-93H18.6		0.79	6.52	<0.0001	0.0003
GBP4	guanylate binding protein 4	0.75	6.48	<0.0001	0.0003
EPSTI1	epithelial stromal interaction 1 (breast)	0.72	6.13	<0.0001	0.0007
GBP5	guanylate binding protein 5	0.79	6.10	<0.0001	0.0007
STAT1	signal transducer and activator of transcription 1, 91kDa	0.74	6.03	<0.0001	0.0007
AIM2	absent in melanoma 2	0.88	5.95	<0.0001	0.0007
FCGR1B		0.95	5.83	<0.0001	0.0009
FCGR1C		0.95	5.83	<0.0001	0.0009
IFI44L	interferon-induced protein 44-like	0.63	5.79	<0.0001	0.001
SERPING1	serpin peptidase inhibitor, clade G (C1 inhibitor), member 1	0.99	5.68	<0.0001	0.0012
DDX60	DEAD (Asp-Glu-Ala-Asp) box polypeptide 60	0.59	5.40	<0.0001	0.002
IFIT2	interferon-induced protein with tetratricopeptide repeats 2	0.76	5.33	<0.0001	0.0022

Gene Name	Gene Description	Log_2 FC	t-Statistic	P	FDR
GBP1P1	guanylate binding protein 1, interferon-inducible pseudogene 1	0.83	5.25	<0.0001	0.0023
SAMD9L	sterile alpha motif domain containing 9-like	0.80	5.25	<0.0001	0.0023
WARS	tryptophanyl-tRNA synthetase	0.61	5.19	<0.0001	0.0026
IFIT3	interferon-induced protein with tetratricopeptide repeats 3	0.74	5.05	<0.0001	0.0035
SMCO4	single-pass membrane protein with coiled-coil domains 4	0.59	4.97	<0.0001	0.0042
TNFSF10	tumor necrosis factor (ligand) superfamily, member 10	0.81	4.95	<0.0001	0.0043
MYOF	myoferlin	0.76	4.91	<0.0001	0.0046
IFI35	interferon-induced protein 35	0.59	4.72	<0.0001	0.0068
CXCL10	chemokine (C-X-C motif) ligand 10	1.08	4.71	<0.0001	0.0068
ANKRD22	ankyrin repeat domain 22	0.87	4.47	<0.0001	0.01
IFIT1	interferon-induced protein with tetratricopeptide repeats 1	0.74	4.43	<0.0001	0.011
TFEC	transcription factor EC	0.67	4.32	0.0001	0.0131
LOC284837	uncharacterized LOC284837	0.68	3.99	0.0003	0.0222
OAS1	2'-5'-oligoadenylate synthetase 1, 40/46kDa	0.59	3.63	0.0009	0.0449
ZNF331	zinc finger protein 331	-0.72	-3.59	0.001	0.0472
CCL20	chemokine (C-C motif) ligand 20	-1.29	-3.72	0.0007	0.0379
C12orf79	chromosome 12 open reading frame 79	-0.62	-3.84	0.0005	0.0295
NR4A3	nuclear receptor subfamily 4, group A, member 3	-0.97	-3.92	0.0004	0.0256
GPR84	G protein-coupled receptor 84	-0.64	-4.06	0.0002	0.0194
OTTHUMG00000178354		-0.60	-4.13	0.0002	0.0174
RP11-769O8.3		-0.60	-4.13	0.0002	0.0174
C15orf48	chromosome 15 open reading frame 48	-0.97	-4.24	0.0001	0.0149
ZNF571	zinc finger protein 571	-0.64	-4.34	0.0001	0.0129
IL1A	interleukin 1, alpha	-0.73	-4.42	<0.0001	0.0112
SERPINB2	serpin peptidase inhibitor, clade B (ovalbumin), member 2	-1.23	-4.42	<0.0001	0.0112
CYP1B1	cytochrome P450, family 1, subfamily B, polypeptide 1	-0.59	-4.64	<0.0001	0.0075
PMP22	peripheral myelin protein 22	-0.62	-4.66	<0.0001	0.0073
CCRL2	chemokine (C-C motif) receptor-like 2	-0.72	-4.74	<0.0001	0.0065
SERTAD1	SERTA domain containing 1	-0.62	-5.10	<0.0001	0.0032
C11orf96	chromosome 11 open reading frame 96	-0.68	-5.25	<0.0001	0.0023
MN1	meningioma (disrupted in balanced translocation) 1	-0.77	-5.32	<0.0001	0.0022
OLR1	oxidized low density lipoprotein (lectin-like) receptor 1	-1.48	-5.37	<0.0001	0.002
PRMT10	protein arginine methyltransferase 10 (putative)	-0.66	-5.43	<0.0001	0.002
CYB5D1	cytochrome b5 domain containing 1	-0.65	-5.54	<0.0001	0.0016
CTH	cystathionase (cystathionine gamma-lyase)	-0.62	-5.76	<0.0001	0.001
SMAD7	SMAD family member 7	-0.70	-5.84	<0.0001	0.0009
PHLDA1	pleckstrin homology-like domain, family A, member 1	-0.95	-5.95	<0.0001	0.0007
NRIP3	nuclear receptor interacting protein 3	-0.97	-5.96	<0.0001	0.0007
FAM177A1	family with sequence similarity 177, member A1	-0.65	-6.39	<0.0001	0.0004

Gene Name	Gene Description	Log_2 FC	t-Statistic	P	FDR
LAMB3	laminin, beta 3	-1.03	-6.88	<0.0001	0.0003
DDIT3	DNA-damage-inducible transcript 3	-0.92	-6.95	<0.0001	0.0003

Table 78: Differentially expressed genes (vaccine study comparisons, DVC-LVS/USAMRIID-LVS (Tularemia), Day 2 vs. 0). The average log_2 fold change was used to combine probe sets with the same gene symbol prior to the analysis. Results are sorted by descending t-statistic. Gene annotations were obtained from Affymetrix (HT HG-U133 Plus PM Annotations, Release 34,10/24/13).

Gene Name	Gene Description	Log_2 FC	t-Statistic	P	FDR
RRM2	ribonucleotide reductase M2	0.83	5.95	<0.0001	0.0004
MS4A14	membrane-spanning 4-domains, subfamily A, member 14	0.68	5.73	<0.0001	0.0005
KIAA0101	KIAA0101	0.78	5.21	<0.0001	0.0015
METTL3	methyltransferase like 3	0.60	5.20	<0.0001	0.0015
TFEC	transcription factor EC	0.63	5.05	<0.0001	0.0018
AIM2	absent in melanoma 2	0.67	4.76	<0.0001	0.0031
HDHD2	haloacid dehalogenase-like hydrolase domain containing 2	0.61	4.70	<0.0001	0.0033
SAMD9L	sterile alpha motif domain containing 9-like	0.68	4.64	<0.0001	0.0038
ATP10D	ATPase, class V, type 10D	0.62	4.56	<0.0001	0.0044
PAQR8	progestin and adipoQ receptor family member VIII	0.71	4.51	<0.0001	0.0048
SLC38A9	solute carrier family 38, member 9	0.60	4.49	<0.0001	0.0049
C9orf69	chromosome 9 open reading frame 69	0.60	4.42	<0.0001	0.0058
TLR5	toll-like receptor 5	0.67	4.34	0.0001	0.0065
GAPT	GRB2-binding adaptor protein, transmembrane	0.63	4.26	0.0001	0.0076
TNFSF10	tumor necrosis factor (ligand) superfamily, member 10	0.59	4.25	0.0001	0.0078
GIMAP4	GTPase, IMAP family member 4	0.61	4.19	0.0002	0.0085
LOC284837	uncharacterized LOC284837	0.70	4.12	0.0002	0.0097
MS4A7	membrane-spanning 4-domains, subfamily A, member 7	0.62	4.07	0.0002	0.0102
OAS1	2'-5'-oligoadenylate synthetase 1, 40/46kDa	0.61	3.96	0.0003	0.0123
TCL1A	T-cell leukemia/lymphoma 1A	0.71	3.84	0.0005	0.0149
CCR2	chemokine (C-C motif) receptor 2	0.59	3.82	0.0005	0.0155
ATG4C	autophagy related 4C, cysteine peptidase	0.61	3.74	0.0006	0.0174
TNFRSF17	tumor necrosis factor receptor superfamily, member 17	0.62	3.41	0.0016	0.0314
TNFAIP6	tumor necrosis factor, alpha-induced protein 6	-0.70	-3.21	0.0027	0.0439
HCAR3	hydroxycarboxylic acid receptor 3	-0.62	-3.24	0.0025	0.042
CCL4	chemokine (C-C motif) ligand 4	-0.70	-3.35	0.0019	0.0349
NAMPT	nicotinamide phosphoribosyltransferase	-0.63	-3.41	0.0016	0.0313
METRNL	meteorin, glial cell differentiation regulator-like	-0.61	-3.75	0.0006	0.017
PTX3	pentraxin 3, long	-0.74	-3.77	0.0006	0.0166
PPP1R15A	protein phosphatase 1, regulatory subunit 15A	-0.68	-3.91	0.0004	0.0129
IER3	immediate early response 3	-0.64	-3.95	0.0003	0.0123

Gene Name	Gene Description	Log_2 FC	t-Statistic	P	FDR
ARL5B	ADP-ribosylation factor-like 5B	-0.68	-4.01	0.0003	0.0114
MIR155		-0.60	-4.01	0.0003	0.0114
MIR155HG		-0.60	-4.01	0.0003	0.0114
CXCL3	chemokine (C-X-C motif) ligand 3	-1.08	-4.02	0.0003	0.0112
C12orf79	chromosome 12 open reading frame 79	-0.68	-4.09	0.0002	0.01
OTTHUMG00000184015		-0.65	-4.12	0.0002	0.0097
RP6-99M1.2		-0.65	-4.12	0.0002	0.0097
ICAM1	intercellular adhesion molecule 1	-0.67	-4.17	0.0002	0.0089
MAP3K8	mitogen-activated protein kinase kinase kinase 8	-0.62	-4.17	0.0002	0.0089
TP53INP2	tumor protein p53 inducible nuclear protein 2	-0.80	-4.18	0.0002	0.0088
MAFF	v-maf musculoaponeurotic fibrosarcoma oncogene homolog F (avian)	-0.97	-4.40	<0.0001	0.0059
F3	coagulation factor III (thromboplastin, tissue factor)	-0.65	-4.41	<0.0001	0.0058
IL1A	interleukin 1, alpha	-0.74	-4.43	<0.0001	0.0055
THBD	thrombomodulin	-0.87	-4.49	<0.0001	0.0049
CCR6	chemokine (C-C motif) receptor 6	-0.59	-4.49	<0.0001	0.0049
CREM	cAMP responsive element modulator	-0.66	-4.51	<0.0001	0.0048
GABARAPL1	GABA(A) receptor-associated protein like 1	-0.64	-4.52	<0.0001	0.0048
DUSP4	dual specificity phosphatase 4	-0.74	-4.53	<0.0001	0.0047
PHACTR1	phosphatase and actin regulator 1	-0.87	-4.60	<0.0001	0.0041
ABCA1	ATP-binding cassette, sub-family A (ABC1), member 1	-0.63	-4.63	<0.0001	0.0039
YES1	v-yes-1 Yamaguchi sarcoma viral oncogene homolog 1	-0.66	-4.68	<0.0001	0.0034
RNMT	RNA (guanine-7-) methyltransferase	-0.62	-4.72	<0.0001	0.0032
CCL20	chemokine (C-C motif) ligand 20	-1.64	-4.72	<0.0001	0.0032
ZBTB21	zinc finger and BTB domain containing 21	-0.66	-4.72	<0.0001	0.0032
MAFB	v-maf musculoaponeurotic fibrosarcoma oncogene homolog B (avian)	-0.68	-4.76	<0.0001	0.0031
SIK3	SIK family kinase 3	-0.62	-4.78	<0.0001	0.003
CYP1B1	cytochrome P450, family 1, subfamily B, polypeptide 1	-0.66	-4.90	<0.0001	0.0024
AVPI1	arginine vasopressin-induced 1	-0.62	-4.93	<0.0001	0.0023
RASGEF1B	RasGEF domain family, member 1B	-0.90	-4.99	<0.0001	0.002
ZNF331	zinc finger protein 331	-1.14	-4.99	<0.0001	0.002
PDCD4	programmed cell death 4 (neoplastic transformation inhibitor)	-0.82	-5.03	<0.0001	0.0019
C11orf96	chromosome 11 open reading frame 96	-0.74	-5.05	<0.0001	0.0018
MPHOSPH6	M-phase phosphoprotein 6	-0.71	-5.10	<0.0001	0.0017
OTTHUMG00000175937		-0.66	-5.12	<0.0001	0.0017
RP11-425D10.10		-0.66	-5.12	<0.0001	0.0017
SLC7A5	solute carrier family 7 (amino acid transporter light chain, L system), member 5	-0.96	-5.20	<0.0001	0.0015
KBTBD8	kelch repeat and BTB (POZ) domain containing 8	-0.77	-5.38	<0.0001	0.0011
JMY	junction mediating and regulatory protein, p53 cofactor	-0.90	-5.41	<0.0001	0.001
RGCC	regulator of cell cycle	-0.76	-5.42	<0.0001	0.001

Gene Name	Gene Description	Log_2 FC	t-Statistic	P	FDR
GPR84	G protein-coupled receptor 84	-0.90	-5.44	<0.0001	0.001
SLC5A6	solute carrier family 5 (sodium-dependent vitamin transporter), member 6	-0.62	-5.45	<0.0001	0.001
CYB5D1	cytochrome b5 domain containing 1	-0.70	-5.53	<0.0001	0.0008
SERPINB2	serpin peptidase inhibitor, clade B (ovalbumin), member 2	-1.72	-5.58	<0.0001	0.0007
PRMT10	protein arginine methyltransferase 10 (putative)	-0.76	-5.59	<0.0001	0.0007
ZNF571	zinc finger protein 571	-0.96	-5.67	<0.0001	0.0006
CCRL2	chemokine (C-C motif) receptor-like 2	-1.01	-5.69	<0.0001	0.0006
NR4A3	nuclear receptor subfamily 4, group A, member 3	-1.52	-5.82	<0.0001	0.0004
GPR132	G protein-coupled receptor 132	-0.61	-5.82	<0.0001	0.0004
USP36	ubiquitin specific peptidase 36	-0.63	-5.87	<0.0001	0.0004
GABPB1	GA binding protein transcription factor, beta subunit 1	-0.69	-5.91	<0.0001	0.0004
PDE4D	phosphodiesterase 4D, cAMP-specific	-0.59	-5.91	<0.0001	0.0004
PPIF	peptidylprolyl isomerase F	-0.77	-5.92	<0.0001	0.0004
ELL2	elongation factor, RNA polymerase II, 2	-0.62	-5.96	<0.0001	0.0004
PHLDA1	pleckstrin homology-like domain, family A, member 1	-1.10	-5.98	<0.0001	0.0004
ID3	inhibitor of DNA binding 3, dominant negative helix-loop-helix protein	-0.66	-6.07	<0.0001	0.0003
RANBP2	RAN binding protein 2	-0.63	-6.08	<0.0001	0.0003
PLAU	plasminogen activator, urokinase	-0.64	-6.09	<0.0001	0.0003
GAS2L3	growth arrest-specific 2 like 3	-0.63	-6.10	<0.0001	0.0003
ZNF184	zinc finger protein 184	-0.73	-6.13	<0.0001	0.0003
ZDBF2	zinc finger, DBF-type containing 2	-0.66	-6.16	<0.0001	0.0003
MN1	meningioma (disrupted in balanced translocation) 1	-1.04	-6.24	<0.0001	0.0003
OLR1	oxidized low density lipoprotein (lectin-like) receptor 1	-1.64	-6.31	<0.0001	0.0002
SMAD7	SMAD family member 7	-0.80	-6.37	<0.0001	0.0002
C15orf48	chromosome 15 open reading frame 48	-1.60	-6.42	<0.0001	0.0002
CTH	cystathionase (cystathionine gamma-lyase)	-0.66	-6.48	<0.0001	0.0002
LAMB3	laminin, beta 3	-1.15	-6.51	<0.0001	0.0002
ADORA2A		-0.75	-6.53	<0.0001	0.0002
SPECC1L-ADORA2A		-0.75	-6.53	<0.0001	0.0002
NRIP3	nuclear receptor interacting protein 3	-1.09	-6.69	<0.0001	0.0002
SERTAD1	SERTA domain containing 1	-0.80	-6.82	<0.0001	0.0002
FAM177A1	family with sequence similarity 177, member A1	-0.73	-7.21	<0.0001	0.0001
DDIT3	DNA-damage-inducible transcript 3	-1.09	-8.07	<0.0001	<0.0001

Table 79: Differentially expressed genes (vaccine study comparisons, DVC-LVS/USAMRIID-LVS (Tularemia), Day 7 vs. 0). The average log_2 fold change was used to combine probe sets with the same gene symbol prior to the analysis. Results are sorted by descending t-statistic. Gene annotations were obtained from Affymetrix (HT HG-U133 Plus PM Annotations, Release 34,10/24/13).

Gene Name	Gene Description	Log_2 FC	t-Statistic	P	FDR
IFI44L	interferon-induced protein 44-like	2.46	7.77	<0.0001	0.0006
SIGLEC1	sialic acid binding Ig-like lectin 1, sialoadhesin	1.71	7.65	<0.0001	0.0006
HERC6	HECT and RLD domain containing E3 ubiquitin protein ligase family member 6	0.59	6.49	<0.0001	0.0047
EPSTI1	epithelial stromal interaction 1 (breast)	1.28	6.05	<0.0001	0.0105
CMPK2	cytidine monophosphate (UMP-CMP) kinase 2, mitochondrial	1.34	5.89	<0.0001	0.0105
USP18	ubiquitin specific peptidase 18	1.06	5.88	<0.0001	0.0105
LOC200772	uncharacterized LOC200772	0.75	5.83	<0.0001	0.0105
IRF7	interferon regulatory factor 7	0.96	5.83	<0.0001	0.0105
IFI44	interferon-induced protein 44	1.44	5.75	<0.0001	0.0107
MX1	myxovirus (influenza virus) resistance 1, interferon-inducible protein p78 (mouse)	1.20	5.74	<0.0001	0.0107
OAS1	2'-5'-oligoadenylate synthetase 1, 40/46kDa	1.38	5.71	<0.0001	0.0107
LINC00487	long intergenic non-protein coding RNA 487	0.74	5.53	<0.0001	0.0153
ISG15	ISG15 ubiquitin-like modifier	1.06	5.40	<0.0001	0.02
XAF1	XIAP associated factor 1	0.99	5.23	<0.0001	0.0283
APOBEC3A		0.96	5.15	<0.0001	0.029
APOBEC3A_B		0.96	5.15	<0.0001	0.029
RSAD2	radical S-adenosyl methionine domain containing 2	1.39	5.05	<0.0001	0.0355
LAMP3	lysosomal-associated membrane protein 3	0.89	4.98	<0.0001	0.0358
OAS2	2'-5'-oligoadenylate synthetase 2, 69/71kDa	0.84	4.96	<0.0001	0.0358
IFIT1	interferon-induced protein with tetratricopeptide repeats 1	1.68	4.94	<0.0001	0.0358
SCO2		0.83	4.82	<0.0001	0.0396
FANCL	Fanconi anemia, complementation group L	0.67	4.81	<0.0001	0.0396
SASH1	SAM and SH3 domain containing 1	0.59	4.76	<0.0001	0.0435
DDX60	DEAD (Asp-Glu-Ala-Asp) box polypeptide 60	0.98	4.74	<0.0001	0.0435
IFI6	interferon, alpha-inducible protein 6	1.02	4.67	<0.0001	0.0483
OAS3	2'-5'-oligoadenylate synthetase 3, 100kDa	0.87	4.66	<0.0001	0.0483

Table 80: Differentially expressed genes (vaccine study comparisons, YF-17D (Yellow Fever), Day 3 vs. 0). The average log_2 fold change was used to combine probe sets with the same gene symbol prior to the analysis. Results are sorted by descending t-statistic. Gene annotations were obtained from Affymetrix (HT HG-U133 Plus PM Annotations, Release 34,10/24/13).

Gene Name	Gene Description	Log_2 FC	t-Statistic	P	FDR
HERC6	HECT and RLD domain containing E3 ubiquitin protein ligase family member 6	1.30	13.93	<0.0001	<0.0001
USP18	ubiquitin specific peptidase 18	2.32	12.75	<0.0001	<0.0001
IFI44L	interferon-induced protein 44-like	3.61	11.82	<0.0001	<0.0001
KLHDC7B	kelch domain containing 7B	0.79	11.73	<0.0001	<0.0001
MX1	myxovirus (influenza virus) resistance 1, interferon-inducible protein p78 (mouse)	2.18	11.68	<0.0001	<0.0001
RGL1	ral guanine nucleotide dissociation stimulator-like 1	1.25	11.27	<0.0001	<0.0001
EPSTI1	epithelial stromal interaction 1 (breast)	2.05	11.07	<0.0001	<0.0001

Gene Name	Gene Description	Log_2 FC	t-Statistic	P	FDR
LAMP3	lysosomal-associated membrane protein 3	2.23	10.97	<0.0001	<0.0001
SIGLEC1	sialic acid binding Ig-like lectin 1, sialoadhesin	2.60	10.85	<0.0001	<0.0001
PARP12		1.00	10.66	<0.0001	<0.0001
XAF1	XIAP associated factor 1	1.83	10.62	<0.0001	<0.0001
IFI6	interferon, alpha-inducible protein 6	1.91	10.58	<0.0001	<0.0001
IFI44	interferon-induced protein 44	2.42	10.47	<0.0001	<0.0001
OAS2	2'-5'-oligoadenylate synthetase 2, 69/71kDa	1.56	10.07	<0.0001	<0.0001
EIF2AK2	eukaryotic translation initiation factor 2-alpha kinase 2	1.02	10.03	<0.0001	<0.0001
PARP9	poly (ADP-ribose) polymerase family, member 9	1.39	9.85	<0.0001	<0.0001
RSAD2	radical S-adenosyl methionine domain containing 2	2.79	9.73	<0.0001	<0.0001
CMPK2	cytidine monophosphate (UMP-CMP) kinase 2, mitochondrial	2.26	9.51	<0.0001	<0.0001
ISG15	ISG15 ubiquitin-like modifier	2.05	9.50	<0.0001	<0.0001
PNPT1		0.98	9.45	<0.0001	<0.0001
HERC5		1.65	9.31	<0.0001	<0.0001
SPATS2L	spermatogenesis associated, serine-rich 2-like	0.84	9.27	<0.0001	<0.0001
MX2		1.53	9.25	<0.0001	<0.0001
DDX60	DEAD (Asp-Glu-Ala-Asp) box polypeptide 60	1.83	9.19	<0.0001	<0.0001
IRF7	interferon regulatory factor 7	1.51	9.17	<0.0001	<0.0001
OAS3	2'-5'-oligoadenylate synthetase 3, 100kDa	1.64	9.16	<0.0001	<0.0001
LINC00487	long intergenic non-protein coding RNA 487	1.78	9.02	<0.0001	<0.0001
OAS1	2'-5'-oligoadenylate synthetase 1, 40/46kDa	2.11	8.84	<0.0001	<0.0001
OASL	2'-5'-oligoadenylate synthetase-like	1.43	8.69	<0.0001	<0.0001
IFIT1	interferon-induced protein with tetratricopeptide repeats 1	3.05	8.60	<0.0001	<0.0001
STAT1	signal transducer and activator of transcription 1, 91kDa	1.01	8.55	<0.0001	<0.0001
BST2		0.83	8.53	<0.0001	<0.0001
DTX3L		0.90	8.48	<0.0001	<0.0001
RNF213	ring finger protein 213	0.59	8.47	<0.0001	<0.0001
IFIT3	interferon-induced protein with tetratricopeptide repeats 3	2.33	8.36	<0.0001	<0.0001
IFI27	interferon, alpha-inducible protein 27	3.16	8.35	<0.0001	<0.0001
PARP14	poly (ADP-ribose) polymerase family, member 14	0.68	8.33	<0.0001	<0.0001
LGALS3BP	lectin, galactoside-binding, soluble, 3 binding protein	1.27	8.18	<0.0001	<0.0001
LOC100507535		0.73	8.08	<0.0001	<0.0001
SERPING1	serpin peptidase inhibitor, clade G (C1 inhibitor), member 1	2.13	8.07	<0.0001	<0.0001
IFI35	interferon-induced protein 35	1.40	7.92	<0.0001	<0.0001
DHX58		0.79	7.89	<0.0001	<0.0001
LY6E	lymphocyte antigen 6 complex, locus E	1.44	7.81	<0.0001	<0.0001
TRIM22		0.83	7.78	<0.0001	<0.0001
NEXN	nexilin (F actin binding protein)	1.48	7.72	<0.0001	<0.0001
ZBP1	Z-DNA binding protein 1	0.76	7.72	<0.0001	<0.0001

Gene Name	Gene Description	Log_2 FC	t-Statistic	P	FDR
SP100	SP100 nuclear antigen	0.60	7.63	<0.0001	<0.0001
CD38	CD38 molecule	0.84	7.55	<0.0001	<0.0001
UBE2L6		0.99	7.48	<0.0001	<0.0001
APOL6	apolipoprotein L, 6	0.64	7.46	<0.0001	<0.0001
CXCL10	chemokine (C-X-C motif) ligand 10	2.35	7.31	<0.0001	<0.0001
LAP3		1.06	7.30	<0.0001	<0.0001
GBP1	guanylate binding protein 1, interferon-inducible	1.24	7.25	<0.0001	<0.0001
APOBEC3A		1.39	7.23	<0.0001	<0.0001
APOBEC3A_B		1.39	7.23	<0.0001	<0.0001
SAMD9L	sterile alpha motif domain containing 9-like	1.77	7.17	<0.0001	<0.0001
FTSJD2		0.61	6.98	<0.0001	<0.0001
SAMD9	sterile alpha motif domain containing 9	1.17	6.95	<0.0001	<0.0001
SAMD4A	sterile alpha motif domain containing 4A	0.72	6.82	<0.0001	0.0001
RRM2	ribonucleotide reductase M2	0.87	6.78	<0.0001	0.0001
SASH1	SAM and SH3 domain containing 1	0.78	6.72	<0.0001	0.0001
PLSCR1	phospholipid scramblase 1	0.81	6.70	<0.0001	0.0001
SCO2		1.23	6.64	<0.0001	0.0002
IFIT5		0.83	6.63	<0.0001	0.0002
RTP4	receptor (chemosensory) transporter protein 4	0.97	6.54	<0.0001	0.0002
CHMP5		0.71	6.52	<0.0001	0.0002
DDX58	DEAD (Asp-Glu-Ala-Asp) box polypeptide 58	0.94	6.50	<0.0001	0.0002
DDX60L	DEAD (Asp-Glu-Ala-Asp) box polypeptide 60-like	0.90	6.47	<0.0001	0.0002
CCR5	chemokine (C-C motif) receptor 5 (gene/pseudogene)	0.71	6.46	<0.0001	0.0002
TOR1B		0.78	6.46	<0.0001	0.0002
TMEM255A	transmembrane protein 255A	1.28	6.45	<0.0001	0.0002
MARCKS	myristoylated alanine-rich protein kinase C substrate	1.15	6.40	<0.0001	0.0003
SP110		0.70	6.31	<0.0001	0.0003
RIN2	Ras and Rab interactor 2	0.79	6.16	<0.0001	0.0004
TDRD7		0.75	6.15	<0.0001	0.0004
IFIH1	interferon induced with helicase C domain 1	0.95	6.15	<0.0001	0.0004
KIAA1958	KIAA1958	0.67	6.11	<0.0001	0.0005
FBXO6	F-box protein 6	0.98	6.02	<0.0001	0.0006
TYMP	thymidine phosphorylase	0.90	6.01	<0.0001	0.0006
IFITM1		0.88	5.96	<0.0001	0.0006
TLR7	toll-like receptor 7	0.87	5.96	<0.0001	0.0006
GMPR	guanosine monophosphate reductase	0.67	5.95	<0.0001	0.0006
CCR1	chemokine (C-C motif) receptor 1	1.13	5.92	<0.0001	0.0007
C3AR1	complement component 3a receptor 1	1.05	5.88	<0.0001	0.0007
MS4A4A	membrane-spanning 4-domains, subfamily A, member 4A	1.29	5.85	<0.0001	0.0008

Gene Name	Gene Description	Log_2 FC	t-Statistic	P	FDR
PI4K2B		0.61	5.66	<0.0001	0.0011
C1QA	complement component 1, q subcomponent, A chain	0.71	5.63	<0.0001	0.0012
LGALS9	lectin, galactoside-binding, soluble, 9	0.59	5.61	<0.0001	0.0012
MT2A		0.64	5.56	<0.0001	0.0014
MYOF	myoferlin	0.86	5.51	<0.0001	0.0015
FAM46A	family with sequence similarity 46, member A	0.64	5.46	<0.0001	0.0017
CCL2	chemokine (C-C motif) ligand 2	0.95	5.35	<0.0001	0.0022
VRK2		0.67	5.32	<0.0001	0.0023
FCGR1B		0.88	5.32	<0.0001	0.0023
IFIT2	interferon-induced protein with tetratricopeptide repeats 2	1.27	5.28	<0.0001	0.0025
IFI16		0.65	5.27	<0.0001	0.0026
KMO	kynurenine 3-monooxygenase (kynurenine 3-hydroxylase)	1.03	5.24	<0.0001	0.0027
PLAC8		0.76	5.22	<0.0001	0.0028
IFITM3		0.95	5.10	<0.0001	0.0037
CECR1		0.67	5.02	<0.0001	0.0043
FCGR1C		0.84	4.98	<0.0001	0.0047
TNFSF10	tumor necrosis factor (ligand) superfamily, member 10	1.13	4.86	<0.0001	0.0061
FANCL	Fanconi anemia, complementation group L	0.77	4.81	<0.0001	0.0067
RAD51AP1	RAD51 associated protein 1	0.64	4.78	<0.0001	0.007
PSME2		0.61	4.78	<0.0001	0.0071
PGAP1	post-GPI attachment to proteins 1	0.67	4.74	<0.0001	0.0076
FAM225A		0.69	4.73	<0.0001	0.0078
FAM225B		0.69	4.73	<0.0001	0.0078
IFITM2		0.76	4.69	<0.0001	0.0085
LOC200772	uncharacterized LOC200772	0.64	4.68	<0.0001	0.0087
NAPA	N-ethylmaleimide-sensitive factor attachment protein, alpha	0.61	4.63	0.0001	0.0097
SLC31A2		0.64	4.62	0.0001	0.0097
CDKN1C	cyclin-dependent kinase inhibitor 1C (p57, Kip2)	0.61	4.57	0.0001	0.0108
CTSL1	cathepsin L1	0.85	4.52	0.0001	0.0121
MAFB	v-maf musculoaponeurotic fibrosarcoma oncogene homolog B (avian)	0.70	4.51	0.0001	0.0122
ANKRD22	ankyrin repeat domain 22	1.11	4.44	0.0002	0.0139
CX3CR1	chemokine (C-X3-C motif) receptor 1	0.74	4.40	0.0002	0.0151
HPSE	heparanase	0.66	4.39	0.0002	0.0154
TCF7L2	transcription factor 7-like 2 (T-cell specific, HMG-box)	0.63	4.36	0.0002	0.0165
WARS	tryptophanyl-tRNA synthetase	0.59	4.30	0.0002	0.0181
CLEC4F	C-type lectin domain family 4, member F	0.66	4.26	0.0003	0.0197
GBP3	guanylate binding protein 3	0.60	4.20	0.0003	0.0219
MS4A7	membrane-spanning 4-domains, subfamily A, member 7	0.73	4.20	0.0003	0.0219
SLC27A3	solute carrier family 27 (fatty acid transporter), member 3	0.69	4.19	0.0003	0.0219

Gene Name	Gene Description	Log_2 FC	t-Statistic	P	FDR
OTTHUMG00000003319		0.69	4.19	0.0003	0.0219
RP4-781L3.1		0.69	4.19	0.0003	0.0219
DUSP6		0.95	4.13	0.0004	0.0248
TNFSF13B		0.63	4.11	0.0004	0.0259
PSMB9		0.59	4.07	0.0004	0.0276
IL15		0.62	4.02	0.0005	0.0306
TMEM140	transmembrane protein 140	0.68	3.97	0.0006	0.0325
NAGK		0.61	3.91	0.0007	0.0361
NLRP3	NLR family, pyrin domain containing 3	0.75	3.88	0.0007	0.0381
XK	X-linked Kx blood group (McLeod syndrome)	0.66	3.84	0.0008	0.0405
KIT	v-kit Hardy-Zuckerman 4 feline sarcoma viral oncogene homolog	-0.62	-4.18	0.0003	0.0224
NGLY1		-0.60	-4.29	0.0003	0.0184
LOC439911		-0.59	-4.58	0.0001	0.0107
RPS23	ribosomal protein S23	-0.68	-5.75	<0.0001	0.0009
ITGAE		-0.60	-7.05	<0.0001	<0.0001

Table 81: Differentially expressed genes (vaccine study comparisons, YF-17D (Yellow Fever), Day 7 vs. 0). The average log_2 fold change was used to combine probe sets with the same gene symbol prior to the analysis. Results are sorted by descending t-statistic. Gene annotations were obtained from Affymetrix (HT HG-U133 Plus PM Annotations, Release 34,10/24/13).

Gene Name	Gene Description	Log_2 FC	t-Statistic	P	FDR
IGHG2		0.99	6.52	<0.0001	0.002
IGKC	immunoglobulin kappa constant	0.80	5.88	<0.0001	0.0056
IGLL3P		0.98	5.68	<0.0001	0.0082
IGK	immunoglobulin kappa locus	0.96	5.57	<0.0001	0.01
IGKV1-17		1.27	5.33	<0.0001	0.0115
AC127391.1		1.13	5.25	<0.0001	0.0115
AC128677.4		1.13	5.25	<0.0001	0.0115
IGKV1OR10-1		1.13	5.25	<0.0001	0.0115
IGKV1OR-2		1.13	5.25	<0.0001	0.0115
IGKV1OR2-118		1.13	5.25	<0.0001	0.0115
OTTHUMG00000155081		1.13	5.25	<0.0001	0.0115
OTTHUMG00000155090		1.13	5.25	<0.0001	0.0115
AC016745.2		1.23	5.24	<0.0001	0.0115
OTTHUMG00000153338		1.23	5.24	<0.0001	0.0115
IGHM	immunoglobulin heavy constant mu	0.61	5.17	<0.0001	0.012
IGHV4-31		0.66	5.16	<0.0001	0.012
IGLV1-36		1.61	5.13	<0.0001	0.0125
IGLJ2		1.19	5.09	<0.0001	0.0127

Gene Name	Gene Description	Log_2 FC	t-Statistic	P	FDR
IGLJ3	immunoglobulin lambda joining 3	1.01	4.95	<0.0001	0.0151
IGHV3-23		0.80	4.94	<0.0001	0.0153
IGLV1-44	immunoglobulin lambda variable 1-44	0.71	4.87	<0.0001	0.0165
IGHD	immunoglobulin heavy constant delta	0.59	4.84	<0.0001	0.0166
IGKV1-37		1.21	4.80	<0.0001	0.0166
IGKV1D-37		1.21	4.80	<0.0001	0.0166
IGLV1-50		1.12	4.78	<0.0001	0.0168
BLOC1S5-TXNDC5		1.13	4.77	<0.0001	0.017
TXNDC5		1.13	4.77	<0.0001	0.017
IGHG3		0.66	4.71	<0.0001	0.0184
IGLV3-19		0.67	4.61	0.0001	0.0217
IGHA2		0.61	4.52	0.0001	0.0242
IGLV3-10		1.24	4.51	0.0001	0.0245
TNFRSF17	tumor necrosis factor receptor superfamily, member 17	1.42	4.47	0.0001	0.0256
PRDX4		0.61	4.45	0.0002	0.0265
MZB1	marginal zone B and B1 cell-specific protein	1.18	4.38	0.0002	0.0292
IGLV2-14		1.07	4.20	0.0003	0.0351
IGJ	immunoglobulin J polypeptide, linker protein for immunoglobulin alpha and mu polypeptides	0.77	4.19	0.0003	0.0359
IGLV3-1		0.88	4.18	0.0003	0.0359
IGKV2-28		0.81	4.05	0.0004	0.0416
IGKV2D-28		0.81	4.05	0.0004	0.0416
SGK1		-0.72	-4.04	0.0004	0.0416
EGR2	early growth response 2	-0.90	-4.17	0.0003	0.0359
GABARAPL3	GABA(A) receptors associated protein like 3, pseudogene	-0.62	-4.22	0.0003	0.0344
CXCL2	chemokine (C-X-C motif) ligand 2	-0.72	-4.22	0.0003	0.0344
IL1B	interleukin 1, beta	-1.26	-4.31	0.0002	0.031
PFKFB3		-0.72	-4.80	<0.0001	0.0166
IL8	interleukin 8	-1.30	-4.82	<0.0001	0.0166
LOC284454	uncharacterized LOC284454	-0.75	-4.97	<0.0001	0.0147
CD83		-0.78	-5.06	<0.0001	0.0129
EGR3	early growth response 3	-0.85	-5.21	<0.0001	0.0115
OSM	oncostatin M	-0.73	-5.22	<0.0001	0.0115
NFIL3		-0.63	-5.32	<0.0001	0.0115
G0S2		-1.04	-5.36	<0.0001	0.0115
RGS1	regulator of G-protein signaling 1	-0.80	-5.42	<0.0001	0.0115
PTGS2	prostaglandin-endoperoxide synthase 2 (prostaglandin G/H synthase and cyclooxygenase)	-1.15	-5.89	<0.0001	0.0056
NR4A3	nuclear receptor subfamily 4, group A, member 3	-0.64	-6.74	<0.0001	0.002
BRE-AS1		-1.53	-7.68	<0.0001	0.0003
NR4A2		-1.39	-7.81	<0.0001	0.0003

Gene Name	Gene Description	Log_2 FC	t-Statistic	P	FDR

Table 82: Differentially expressed genes (vaccine study comparisons, TIV (Influenza), Day 7 vs. 0). The average log_2 fold change was used to combine probe sets with the same gene symbol prior to the analysis. Results are sorted by descending t-statistic. Gene annotations were obtained from Affymetrix (HT HG-U133 Plus PM Annotations, Release 34,10/24/13).

Gene Name	Gene Description	Log_2 FC	t-Statistic	P	FDR
OAS1	2'-5'-oligoadenylate synthetase 1, 40/46kDa	0.96	7.89	<0.0001	0.0002
SMCO4	single-pass membrane protein with coiled-coil domains 4	0.82	7.11	<0.0001	0.0003
KCTD12		0.60	5.76	<0.0001	0.0011
IDH1	isocitrate dehydrogenase 1 (NADP+), soluble	0.64	5.30	<0.0001	0.0015
RAB10	RAB10, member RAS oncogene family	0.69	5.21	<0.0001	0.0017
TNFSF10	tumor necrosis factor (ligand) superfamily, member 10	0.70	5.18	<0.0001	0.0018
GAPT	GRB2-binding adaptor protein, transmembrane	0.62	4.29	0.0002	0.0069
CCR2	chemokine (C-C motif) receptor 2	0.71	3.94	0.0005	0.0115
GIMAP4	GTPase, IMAP family member 4	0.67	3.86	0.0006	0.0131
SLC25A37	solute carrier family 25 (mitochondrial iron transporter), member 37	-0.62	-3.04	0.0052	0.0496
PFKFB3		-0.66	-3.22	0.0034	0.0373
IL8	interleukin 8	-1.16	-3.27	0.0029	0.0343
THBS1	thrombospondin 1	-0.60	-3.35	0.0024	0.0298
BCL2A1		-0.74	-3.46	0.0018	0.025
MXD1	MAX dimerization protein 1	-0.71	-3.60	0.0013	0.0202
NR4A2		-0.73	-3.69	0.001	0.0173
OSM	oncostatin M	-0.72	-3.90	0.0006	0.0123
CSRNP1	cysteine-serine-rich nuclear protein 1	-0.62	-3.98	0.0005	0.011
NAMPT	nicotinamide phosphoribosyltransferase	-0.92	-4.22	0.0002	0.0075
RPS27		-0.59	-4.26	0.0002	0.007
G0S2		-1.28	-4.27	0.0002	0.007
SLC2A14		-0.65	-4.42	0.0001	0.0057
SLC2A3	solute carrier family 2 (facilitated glucose transporter), member 3	-0.69	-4.68	<0.0001	0.0037
GPR183		-0.62	-4.71	<0.0001	0.0036
TP53INP2	tumor protein p53 inducible nuclear protein 2	-0.63	-4.85	<0.0001	0.0029
ATG2A	autophagy related 2A	-0.67	-4.88	<0.0001	0.0027
PDE4B	phosphodiesterase 4B, cAMP-specific	-0.82	-5.00	<0.0001	0.0022
METRNL	meteorin, glial cell differentiation regulator-like	-0.59	-5.05	<0.0001	0.0021
NXT1		-0.60	-5.05	<0.0001	0.0021
BTG3	BTG family, member 3	-0.62	-5.07	<0.0001	0.002
GABARAPL3	GABA(A) receptors associated protein like 3, pseudogene	-0.75	-5.08	<0.0001	0.002
GABARAPL1	GABA(A) receptor-associated protein like 1	-0.64	-5.11	<0.0001	0.0019
MAFF	v-maf musculoaponeurotic fibrosarcoma oncogene homolog F (avian)	-1.02	-5.16	<0.0001	0.0018

Gene Name	Gene Description	Log_2 FC	t-Statistic	P	FDR
ZNF331	zinc finger protein 331	-1.14	-5.28	<0.0001	0.0015
JOSD1		-0.59	-6.23	<0.0001	0.0006
JMY	junction mediating and regulatory protein, p53 cofactor	-0.63	-6.35	<0.0001	0.0005
ZBTB21	zinc finger and BTB domain containing 21	-0.80	-6.84	<0.0001	0.0003
CREM	cAMP responsive element modulator	-0.79	-7.48	<0.0001	0.0002

Table 83: Differentially expressed genes (vaccine study comparisons, LAIV (Influenza), Day 3 vs. 0). The average log_2 fold change was used to combine probe sets with the same gene symbol prior to the analysis. Results are sorted by descending t-statistic. Gene annotations were obtained from Affymetrix (HT HG-U133 Plus PM Annotations, Release 34,10/24/13).

Gene Name	Gene Description	Log_2 FC	t-Statistic	P	FDR
LOC100506776	uncharacterized LOC100506776	0.69	5.06	<0.0001	0.0026
KCTD12		0.59	4.95	<0.0001	0.0029
DENND2D	DENN/MADD domain containing 2D	0.60	4.93	<0.0001	0.0029
OAS1	2'-5'-oligoadenylate synthetase 1, 40/46kDa	0.80	4.91	<0.0001	0.003
SGK223	homolog of rat pragma of Rnd2	0.60	4.51	0.0001	0.0047
MS4A7	membrane-spanning 4-domains, subfamily A, member 7	0.63	4.39	0.0002	0.0054
IDH1	isocitrate dehydrogenase 1 (NADP+), soluble	0.68	4.28	0.0002	0.0062
GIMAP4	GTPase, IMAP family member 4	0.76	4.25	0.0002	0.0065
GIMAP6		0.62	4.11	0.0004	0.0077
RAB10	RAB10, member RAS oncogene family	0.69	4.09	0.0004	0.0079
SMCO4	single-pass membrane protein with coiled-coil domains 4	0.67	4.02	0.0004	0.0086
COPB2	coatomer protein complex, subunit beta 2 (beta prime)	0.59	3.98	0.0005	0.0092
GAPT	GRB2-binding adaptor protein, transmembrane	0.65	3.83	0.0007	0.0117
CCR2	chemokine (C-C motif) receptor 2	0.75	3.26	0.0031	0.0294
SLC25A37	solute carrier family 25 (mitochondrial iron transporter), member 37	-0.72	-2.96	0.0066	0.0469
SGK1		-0.66	-3.05	0.0052	0.0406
THBS1	thrombospondin 1	-0.68	-3.07	0.005	0.0394
CLC	Charcot-Leyden crystal galectin	-1.62	-3.12	0.0044	0.0361
CXCL2	chemokine (C-X-C motif) ligand 2	-0.59	-3.22	0.0035	0.0315
PROK2	prokineticin 2	-0.76	-3.25	0.0032	0.03
BTG1		-0.63	-3.25	0.0032	0.0299
RAB20	RAB20, member RAS oncogene family	-0.74	-3.29	0.0029	0.0284
S100P	S100 calcium binding protein P	-1.18	-3.38	0.0023	0.0247
PLAUR	plasminogen activator, urokinase receptor	-0.60	-3.58	0.0014	0.0176
CXCL1	chemokine (C-X-C motif) ligand 1 (melanoma growth stimulating activity, alpha)	-0.77	-3.65	0.0012	0.0159
ACSL1	acyl-CoA synthetase long-chain family member 1	-0.88	-3.75	0.0009	0.0133
B3GNT5	UDP-GlcNAc:betaGal beta-1,3-N-acetylglucosaminyltransferase 5	-0.59	-3.93	0.0006	0.01
C5AR1		-0.62	-3.96	0.0005	0.0094

Gene Name	Gene Description	Log_2 FC	t-Statistic	P	FDR
AREGB		-0.95	-3.97	0.0005	0.0093
TREM1	triggering receptor expressed on myeloid cells 1	-0.78	-4.03	0.0004	0.0084
C1orf132		-0.60	-4.04	0.0004	0.0083
TMCC3	transmembrane and coiled-coil domain family 3	-0.59	-4.13	0.0003	0.0074
WHAMM		-0.59	-4.14	0.0003	0.0073
CD83		-0.75	-4.20	0.0003	0.007
MXD1	MAX dimerization protein 1	-0.85	-4.23	0.0003	0.0067
EGR2	early growth response 2	-0.72	-4.24	0.0002	0.0065
NXT1		-0.60	-4.29	0.0002	0.0062
BCL2A1		-0.84	-4.34	0.0002	0.0058
DBF4		-0.59	-4.34	0.0002	0.0058
OSM	oncostatin M	-0.89	-4.37	0.0002	0.0055
NRBF2		-0.59	-4.40	0.0002	0.0053
FAM46C	family with sequence similarity 46, member C	-0.62	-4.44	0.0001	0.0051
BTG3	BTG family, member 3	-0.63	-4.50	0.0001	0.0047
PFKFB3		-1.04	-4.50	0.0001	0.0047
THBD	thrombomodulin	-0.61	-4.54	0.0001	0.0046
SAMSN1	SAM domain, SH3 domain and nuclear localization signals 1	-0.61	-4.66	<0.0001	0.0039
IL8	interleukin 8	-1.47	-4.68	<0.0001	0.0038
EGR3	early growth response 3	-0.74	-4.69	<0.0001	0.0038
MAFF	v-maf musculoaponeurotic fibrosarcoma oncogene homolog F (avian)	-0.95	-4.70	<0.0001	0.0037
PHACTR1	phosphatase and actin regulator 1	-0.74	-4.78	<0.0001	0.0034
OTTHUMG00000176282		-0.70	-4.79	<0.0001	0.0033
RP11-469M7.1		-0.70	-4.79	<0.0001	0.0033
ATG2A	autophagy related 2A	-0.74	-4.85	<0.0001	0.0032
TAF13	TAF13 RNA polymerase II, TATA box binding protein (TBP)-associated factor, 18kDa	-0.60	-4.97	<0.0001	0.0028
MAPK6		-0.64	-5.02	<0.0001	0.0027
TP53INP2	tumor protein p53 inducible nuclear protein 2	-0.60	-5.04	<0.0001	0.0027
BRE-AS1		-1.19	-5.08	<0.0001	0.0025
NFIL3		-0.91	-5.10	<0.0001	0.0025
SLC2A14		-0.84	-5.13	<0.0001	0.0025
FAM53C		-0.64	-5.14	<0.0001	0.0025
ZNF331	zinc finger protein 331	-1.18	-5.17	<0.0001	0.0025
RGS1	regulator of G-protein signaling 1	-0.73	-5.18	<0.0001	0.0025
GABARAPL1	GABA(A) receptor-associated protein like 1	-0.64	-5.26	<0.0001	0.0023
PTGS2	prostaglandin-endoperoxide synthase 2 (prostaglandin G/H synthase and cyclooxygenase)	-1.48	-5.35	<0.0001	0.0022
GABARAPL3	GABA(A) receptors associated protein like 3, pseudogene	-0.80	-5.35	<0.0001	0.0022
SLC2A3	solute carrier family 2 (facilitated glucose transporter), member 3	-0.86	-5.36	<0.0001	0.0022
SPTY2D1	SPT2, Suppressor of Ty, domain containing 1 (S. cerevisiae)	-0.62	-5.39	<0.0001	0.0022

Gene Name	Gene Description	Log_2 FC	t-Statistic	P	FDR
JMY	junction mediating and regulatory protein, p53 cofactor	-0.59	-5.44	<0.0001	0.0022
G0S2		-1.43	-5.48	<0.0001	0.0021
NR4A3	nuclear receptor subfamily 4, group A, member 3	-0.69	-5.56	<0.0001	0.0021
PDE4B	phosphodiesterase 4B, cAMP-specific	-0.94	-5.95	<0.0001	0.0014
GPR183		-0.70	-5.96	<0.0001	0.0014
CSRNP1	cysteine-serine-rich nuclear protein 1	-0.79	-5.97	<0.0001	0.0014
ZNF267	zinc finger protein 267	-0.73	-6.11	<0.0001	0.0013
HSPA13	heat shock protein 70kDa family, member 13	-0.62	-6.28	<0.0001	0.001
NAMPT	nicotinamide phosphoribosyltransferase	-1.25	-6.30	<0.0001	0.001
GTF2B		-0.66	-6.33	<0.0001	0.001
ZBTB21	zinc finger and BTB domain containing 21	-0.90	-6.93	<0.0001	0.0006
NR4A2		-1.25	-6.98	<0.0001	0.0006
CREM	cAMP responsive element modulator	-0.85	-7.61	<0.0001	0.0006

Table 84: Differentially expressed genes (vaccine study comparisons, LAIV (Influenza), Day 7 vs. 0). The average log_2 fold change was used to combine probe sets with the same gene symbol prior to the analysis. Results are sorted by descending t-statistic. Gene annotations were obtained from Affymetrix (HT HG-U133 Plus PM Annotations, Release 34,10/24/13).

Gene Name	Gene Description	Log_2 FC	t-Statistic	P	FDR
IL1A	interleukin 1, alpha	-0.86	-4.95	<0.0001	0.0462
SIGLEC1	sialic acid binding Ig-like lectin 1, sialoadhesin	-1.30	-5.20	<0.0001	0.0462
IFI44L	interferon-induced protein 44-like	-1.83	-5.46	<0.0001	0.0462
LAMB3	laminin, beta 3	-1.05	-6.13	<0.0001	0.0017

Table 85: Differentially expressed genes (vaccine study comparisons, DVC-LVS/USAMRIID-LVS (Tularemia) Day 2 vs. YF-17D (Yellow Fever) Day 3). The average log_2 fold change was used to combine probe sets with the same gene symbol prior to the analysis. Results are sorted by descending t-statistic. Gene annotations were obtained from Affymetrix (HT HG-U133 Plus PM Annotations, Release 34,10/24/13).

Gene Name	Gene Description	Log_2 FC	t-Statistic	P	FDR
LOC100507018	uncharacterized LOC100507018	0.70	5.42	<0.0001	0.0004
RPS23	ribosomal protein S23	0.71	4.78	<0.0001	0.0027
LOC100506328	uncharacterized LOC100506328	0.69	4.54	<0.0001	0.004
N4BP2L2-IT2	N4BPL2 intronic transcript 2 (non-protein coding)	0.68	4.37	<0.0001	0.0068
TAPT1-AS1	TAPT1 antisense RNA 1 (head to head)	0.59	4.27	<0.0001	0.0086
METTL3	methyltransferase like 3	0.63	4.20	<0.0001	0.0104
CTB-167B5.2		1.11	4.15	0.0001	0.0132
OTTHUMG00000179513		1.11	4.15	0.0001	0.0132
ST20	suppressor of tumorigenicity 20	0.73	4.15	0.0001	0.0132

Gene Name	Gene Description	Log_2 FC	t-Statistic	P	FDR
JMY	junction mediating and regulatory protein, p53 cofactor	-0.76	-3.52	0.0008	0.0494
GMPR	guanosine monophosphate reductase	-0.59	-3.59	0.0007	0.0433
FAM225A		-0.64	-3.61	0.0008	0.0467
FAM225B		-0.64	-3.61	0.0008	0.0467
TYMP	thymidine phosphorylase	-0.71	-3.65	0.0006	0.0405
NR4A3	nuclear receptor subfamily 4, group A, member 3	-1.50	-3.68	0.0005	0.0388
CCL4	chemokine (C-C motif) ligand 4	-1.33	-3.70	0.0006	0.039
SAMD9L	sterile alpha motif domain containing 9-like	-1.09	-3.78	0.0005	0.0367
USP36	ubiquitin specific peptidase 36	-0.59	-3.85	0.0003	0.0251
CDKN1A	cyclin-dependent kinase inhibitor 1A (p21, Cip1)	-0.87	-3.89	0.0003	0.0237
NAPA	N-ethylmaleimide-sensitive factor attachment protein, alpha	-0.73	-3.89	0.0003	0.0225
CCL20	chemokine (C-C motif) ligand 20	-2.03	-3.96	0.0002	0.0197
PIWIL4	piwi-like RNA-mediated gene silencing 4	-0.60	-3.99	0.0002	0.0197
PPP1R15A	protein phosphatase 1, regulatory subunit 15A	-1.01	-3.99	0.0002	0.0175
OTTHUMG00000184015		-0.90	-4.02	0.0002	0.016
RP6-99M1.2		-0.90	-4.02	0.0002	0.016
CCRN4L	CCR4 carbon catabolite repression 4-like (S. cerevisiae)	-0.61	-4.06	0.0002	0.0152
AVPI1	arginine vasopressin-induced 1	-0.78	-4.07	0.0002	0.0148
FNIP2	folliculin interacting protein 2	-0.75	-4.09	0.0001	0.0141
MS4A4A	membrane-spanning 4-domains, subfamily A, member 4A	-1.01	-4.11	0.0002	0.02
SLC5A6	solute carrier family 5 (sodium-dependent vitamin transporter), member 6	-0.63	-4.12	0.0001	0.0128
C1QA	complement component 1, q subcomponent, A chain	-0.67	-4.15	0.0001	0.0132
SEMA4A	sema domain, immunoglobulin domain (Ig), transmembrane domain (TM) and short cytoplasmic domain, (semaphorin) 4A	-0.60	-4.18	<0.0001	0.011
RTP4	receptor (chemosensory) transporter protein 4	-0.75	-4.21	0.0001	0.0132
ADORA2B	adenosine A2b receptor	-0.73	-4.21	<0.0001	0.0101
TNF	tumor necrosis factor	-1.34	-4.23	0.0001	0.012
SPHK1	sphingosine kinase 1	-0.64	-4.38	<0.0001	0.0068
RASGEF1B	RasGEF domain family, member 1B	-1.21	-4.39	<0.0001	0.0071
PTX3	pentraxin 3, long	-1.43	-4.43	<0.0001	0.007
HAVCR2	hepatitis A virus cellular receptor 2	-0.61	-4.45	<0.0001	0.0052
GBP1	guanylate binding protein 1, interferon-inducible	-0.90	-4.47	<0.0001	0.0075
C15orf48	chromosome 15 open reading frame 48	-2.21	-4.50	<0.0001	0.0074
DDIT3	DNA-damage-inducible transcript 3	-0.92	-4.59	<0.0001	0.0039
MN1	meningioma (disrupted in balanced translocation) 1	-1.21	-4.61	<0.0001	0.004
NLRP3	NLR family, pyrin domain containing 3	-1.18	-4.63	<0.0001	0.0037
EMP1	epithelial membrane protein 1	-0.88	-4.67	<0.0001	0.0031
FAM46A	family with sequence similarity 46, member A	-0.61	-4.68	<0.0001	0.0055
SERTAD1	SERTA domain containing 1	-0.99	-4.68	<0.0001	0.004

Gene Name	Gene Description	Log_2 FC	t-Statistic	P	FDR
SIK3	SIK family kinase 3	-0.68	-4.74	<0.0001	0.0028
DCUN1D3	DCN1, defective in cullin neddylation 1, domain containing 3	-0.60	-4.75	<0.0001	0.0022
LRRC32	leucine rich repeat containing 32	-0.62	-4.76	<0.0001	0.0022
ZBP1	Z-DNA binding protein 1	-0.60	-4.80	<0.0001	0.0024
C10orf137	chromosome 10 open reading frame 137	-0.60	-4.81	<0.0001	0.0023
CUL1	cullin 1	-0.76	-4.82	<0.0001	0.0019
DMXL2	Dmx-like 2	-0.96	-4.86	<0.0001	0.0017
F3	coagulation factor III (thromboplastin, tissue factor)	-1.09	-4.87	<0.0001	0.0018
PHLDA1	pleckstrin homology-like domain, family A, member 1	-1.36	-4.93	<0.0001	0.0015
OLR1	oxidized low density lipoprotein (lectin-like) receptor 1	-1.56	-4.94	<0.0001	0.0013
ADORA2A		-0.80	-4.99	<0.0001	0.0011
SPECC1L-ADORA2A		-0.80	-4.99	<0.0001	0.0011
CNP	2',3'-cyclic nucleotide 3' phosphodiesterase	-0.59	-5.00	<0.0001	0.0011
PLSCR1	phospholipid scramblase 1	-0.83	-5.01	<0.0001	0.0011
SAMD4A	sterile alpha motif domain containing 4A	-0.61	-5.14	<0.0001	0.0016
VSIG10L	V-set and immunoglobulin domain containing 10 like	-0.59	-5.16	<0.0001	0.0007
IFI35	interferon-induced protein 35	-1.10	-5.17	<0.0001	0.0011
NRIP3	nuclear receptor interacting protein 3	-1.38	-5.17	<0.0001	0.0009
OAS1	2'-5'-oligoadenylate synthetase 1, 40/46kDa	-1.50	-5.28	<0.0001	0.0009
CTSL1	cathepsin L1	-1.20	-5.30	<0.0001	0.0008
IL1A	interleukin 1, alpha	-0.94	-5.31	<0.0001	0.0008
NEXN	nexilin (F actin binding protein)	-1.29	-5.32	<0.0001	0.0006
SAMD9	sterile alpha motif domain containing 9	-1.06	-5.32	<0.0001	0.0008
GADD45B	growth arrest and DNA-damage-inducible, beta	-0.70	-5.35	<0.0001	0.0004
GAS2L3	growth arrest-specific 2 like 3	-0.78	-5.36	<0.0001	0.0004
CYB5D1	cytochrome b5 domain containing 1	-0.82	-5.38	<0.0001	0.0004
MASTL	microtubule associated serine/threonine kinase-like	-1.00	-5.45	<0.0001	0.0006
PHLDA2	pleckstrin homology-like domain, family A, member 2	-0.65	-5.46	<0.0001	0.0005
IL1RN	interleukin 1 receptor antagonist	-0.65	-5.46	<0.0001	0.0003
CCL2	chemokine (C-C motif) ligand 2	-1.34	-5.47	<0.0001	0.0003
DDX60L	DEAD (Asp-Glu-Ala-Asp) box polypeptide 60-like	-0.88	-5.57	<0.0001	0.0006
PGAP1	post-GPI attachment to proteins 1	-0.86	-5.60	<0.0001	0.0008
IFIH1	interferon induced with helicase C domain 1	-0.97	-5.68	<0.0001	0.0005
RIN2	Ras and Rab interactor 2	-1.03	-5.71	<0.0001	0.0001
TMEM255A	transmembrane protein 255A	-1.22	-5.74	<0.0001	0.0006
CCRL2	chemokine (C-C motif) receptor-like 2	-1.39	-5.76	<0.0001	0.0001
C3AR1	complement component 3a receptor 1	-1.25	-5.78	<0.0001	0.0002
STAT1	signal transducer and activator of transcription 1, 91kDa	-0.83	-5.79	<0.0001	0.0002
CXCL10	chemokine (C-X-C motif) ligand 10	-2.11	-5.85	<0.0001	0.0003

Gene Name	Gene Description	Log_2 FC	t-Statistic	P	FDR
GPR84	G protein-coupled receptor 84	-1.40	-5.89	<0.0001	<0.0001
LGALS3BP	lectin, galactoside-binding, soluble, 3 binding protein	-1.07	-6.03	<0.0001	0.0002
LCP2	lymphocyte cytosolic protein 2 (SH2 domain containing leukocyte protein of 76kDa)	-0.80	-6.04	<0.0001	<0.0001
LY6E	lymphocyte antigen 6 complex, locus E	-1.42	-6.07	<0.0001	<0.0001
FOSL1	FOS-like antigen 1	-0.94	-6.08	<0.0001	<0.0001
CCR1	chemokine (C-C motif) receptor 1	-1.40	-6.12	<0.0001	<0.0001
DDX60	DEAD (Asp-Glu-Ala-Asp) box polypeptide 60	-1.40	-6.17	<0.0001	0.0001
PARP14	poly (ADP-ribose) polymerase family, member 14	-0.68	-6.17	<0.0001	<0.0001
SERPING1	serpin peptidase inhibitor, clade G (C1 inhibitor), member 1	-1.79	-6.24	<0.0001	0.0002
IFIT1	interferon-induced protein with tetratricopeptide repeats 1	-2.57	-6.27	<0.0001	<0.0001
IFIT3	interferon-induced protein with tetratricopeptide repeats 3	-2.00	-6.32	<0.0001	<0.0001
LAMB3	laminin, beta 3	-1.32	-6.34	<0.0001	<0.0001
ZCCHC2	zinc finger, CCHC domain containing 2	-0.68	-6.40	<0.0001	<0.0001
PARP9	poly (ADP-ribose) polymerase family, member 9	-1.08	-6.52	<0.0001	<0.0001
KIAA1958	KIAA1958	-0.83	-6.53	<0.0001	<0.0001
MAFB	v-maf musculoaponeurotic fibrosarcoma oncogene homolog B (avian)	-1.38	-6.55	<0.0001	<0.0001
MARCKS	myristoylated alanine-rich protein kinase C substrate	-1.29	-6.59	<0.0001	<0.0001
KLHDC7B	kelch domain containing 7B	-0.64	-6.82	<0.0001	<0.0001
OAS2	2'-5'-oligoadenylate synthetase 2, 69/71kDa	-1.21	-7.00	<0.0001	<0.0001
XAF1	XIAP associated factor 1	-1.47	-7.20	<0.0001	<0.0001
OAS3	2'-5'-oligoadenylate synthetase 3, 100kDa	-1.54	-7.49	<0.0001	<0.0001
IRF7	interferon regulatory factor 7	-1.40	-7.52	<0.0001	<0.0001
ISG15	ISG15 ubiquitin-like modifier	-1.91	-7.73	<0.0001	<0.0001
SPATS2L	spermatogenesis associated, serine-rich 2-like	-0.79	-7.76	<0.0001	<0.0001
CMPK2	cytidine monophosphate (UMP-CMP) kinase 2, mitochondrial	-2.08	-7.78	<0.0001	<0.0001
RSAD2	radical S-adenosyl methionine domain containing 2	-2.48	-7.79	<0.0001	<0.0001
LDLR	low density lipoprotein receptor	-0.87	-7.94	<0.0001	<0.0001
IFI27	interferon, alpha-inducible protein 27	-3.19	-8.02	<0.0001	<0.0001
LINC00487	long intergenic non-protein coding RNA 487	-1.68	-8.03	<0.0001	<0.0001
IFI6	interferon, alpha-inducible protein 6	-1.78	-8.13	<0.0001	<0.0001
EPSTI1	epithelial stromal interaction 1 (breast)	-1.79	-8.25	<0.0001	<0.0001
OASL	2'-5'-oligoadenylate synthetase-like	-1.69	-8.28	<0.0001	<0.0001
IFI44	interferon-induced protein 44	-2.19	-8.66	<0.0001	<0.0001
EIF2AK2	eukaryotic translation initiation factor 2-alpha kinase 2	-1.09	-8.81	<0.0001	<0.0001
MX1	myxovirus (influenza virus) resistance 1, interferon-inducible protein p78 (mouse)	-1.98	-8.97	<0.0001	<0.0001
SIGLEC1	sialic acid binding Ig-like lectin 1, sialoadhesin	-2.46	-9.20	<0.0001	<0.0001
RGL1	ral guanine nucleotide dissociation stimulator-like 1	-1.48	-9.59	<0.0001	<0.0001
LAMP3	lysosomal-associated membrane protein 3	-2.12	-9.63	<0.0001	<0.0001
IFI44L	interferon-induced protein 44-like	-3.30	-9.72	<0.0001	<0.0001

Gene Name	Gene Description	Log_2 FC	t-Statistic	P	FDR
USP18	ubiquitin specific peptidase 18	-2.24	-10.20	<0.0001	<0.0001
HERC6	HECT and RLD domain containing E3 ubiquitin protein ligase family member 6	-1.20	-11.43	<0.0001	<0.0001

Table 86: Differentially expressed genes (vaccine study comparisons, DVC-LVS/USAMRIID-LVS (Tularemia) Day 7 vs. YF-17D (Yellow Fever) Day 7). The average log_2 fold change was used to combine probe sets with the same gene symbol prior to the analysis. Results are sorted by descending t-statistic. Gene annotations were obtained from Affymetrix (HT HG-U133 Plus PM Annotations, Release 34,10/24/13).

Gene Name	Gene Description	Log_2 FC	t-Statistic	P	FDR
METTL3	methyltransferase like 3	0.59	4.28	<0.0001	0.0308
ERV3-2	endogenous retrovirus group 3, member 2	0.67	4.25	0.0001	0.0414
GBP1P1	guanylate binding protein 1, interferon-inducible pseudogene 1	0.82	4.12	0.0001	0.0415
TMEM52B	transmembrane protein 52B	-0.59	-4.19	0.0001	0.046
CYP1B1	cytochrome P450, family 1, subfamily B, polypeptide 1	-0.79	-4.19	<0.0001	0.0411
CCRL2	chemokine (C-C motif) receptor-like 2	-0.71	-4.48	<0.0001	0.0303
OLR1	oxidized low density lipoprotein (lectin-like) receptor 1	-1.35	-4.61	<0.0001	0.0225
IL1A	interleukin 1, alpha	-0.88	-4.69	<0.0001	0.0174
C11orf96	chromosome 11 open reading frame 96	-0.67	-4.91	<0.0001	0.0174
CYB5D1	cytochrome b5 domain containing 1	-0.64	-5.05	<0.0001	0.0134
PHLDA1	pleckstrin homology-like domain, family A, member 1	-0.91	-5.53	<0.0001	0.0087
FAM177A1	family with sequence similarity 177, member A1	-0.76	-5.56	<0.0001	0.0066
CTH	cystathionase (cystathionine gamma-lyase)	-0.65	-5.59	<0.0001	0.0066
NRIP3	nuclear receptor interacting protein 3	-0.95	-5.71	<0.0001	0.0066

Table 87: Differentially expressed genes (vaccine study comparisons, DVC-LVS/USAMRIID-LVS (Tularemia) Day 2 vs. TIV (Influenza) Day 3). The average log_2 fold change was used to combine probe sets with the same gene symbol prior to the analysis. Results are sorted by descending t-statistic. Gene annotations were obtained from Affymetrix (HT HG-U133 Plus PM Annotations, Release 34,10/24/13).

Gene Name	Gene Description	Log_2 FC	t-Statistic	P	FDR
PTPRE	protein tyrosine phosphatase, receptor type, E	0.65	5.80	<0.0001	0.001
PTGS2	prostaglandin-endoperoxide synthase 2 (prostaglandin G/H synthase and cyclooxygenase)	1.14	3.92	0.0002	0.0316
IGKC	immunoglobulin kappa constant	-0.64	-3.73	0.0005	0.0464
IGLV3-1		-0.93	-3.79	0.0005	0.0464
CXCL3	chemokine (C-X-C motif) ligand 3	-1.04	-3.85	0.0004	0.0438
CCL20	chemokine (C-C motif) ligand 20	-1.39	-3.86	0.0004	0.0406
SMAD7	SMAD family member 7	-0.68	-3.99	0.0002	0.0283
ZNF571	zinc finger protein 571	-0.75	-4.03	0.0002	0.0284
SERPINB2	serpin peptidase inhibitor, clade B (ovalbumin), member 2	-1.33	-4.04	0.0002	0.0296
DDIT3	DNA-damage-inducible transcript 3	-0.76	-4.10	0.0001	0.0234

Gene Name	Gene Description	Log_2 FC	t-Statistic	P	FDR
CYP1B1	cytochrome P450, family 1, subfamily B, polypeptide 1	-0.74	-4.13	0.0001	0.0223
PPIF	peptidylprolyl isomerase F	-0.63	-4.13	0.0001	0.0228
MAN1A1	mannosidase, alpha, class 1A, member 1	-0.62	-4.22	0.0001	0.0218
C11orf96	chromosome 11 open reading frame 96	-0.64	-4.22	0.0001	0.0234
MIR155		-0.69	-4.26	<0.0001	0.02
MIR155HG		-0.69	-4.26	<0.0001	0.02
CLEC5A	C-type lectin domain family 5, member A	-0.70	-4.29	0.0001	0.0211
PRMT10	protein arginine methyltransferase 10 (putative)	-0.71	-4.31	<0.0001	0.0161
SIK3	SIK family kinase 3	-0.60	-4.34	<0.0001	0.0176
PDCD4	programmed cell death 4 (neoplastic transformation inhibitor)	-0.79	-4.35	<0.0001	0.0161
ID3	inhibitor of DNA binding 3, dominant negative helix-loop-helix protein	-0.61	-4.37	<0.0001	0.0141
GABPB1	GA binding protein transcription factor, beta subunit 1	-0.60	-4.42	<0.0001	0.0136
ELL2	elongation factor, RNA polymerase II, 2	-0.79	-4.56	<0.0001	0.0107
IGHG2		-0.81	-4.78	<0.0001	0.0103
MPHOSPH6	M-phase phosphoprotein 6	-0.69	-4.81	<0.0001	0.0078
GPR84	G protein-coupled receptor 84	-0.86	-4.85	<0.0001	0.006
IL1A	interleukin 1, alpha	-0.91	-4.97	<0.0001	0.0046
CCRL2	chemokine (C-C motif) receptor-like 2	-0.93	-5.06	<0.0001	0.0047
GPR132	G protein-coupled receptor 132	-0.62	-5.18	<0.0001	0.0023
ADORA2A		-0.72	-5.22	<0.0001	0.0018
SPECC1L-ADORA2A		-0.72	-5.22	<0.0001	0.0018
CYB5D1	cytochrome b5 domain containing 1	-0.70	-5.27	<0.0001	0.0023
SEC24A	SEC24 family, member A (S. cerevisiae)	-0.63	-5.36	<0.0001	0.0014
PHLDA1	pleckstrin homology-like domain, family A, member 1	-1.00	-5.40	<0.0001	0.0023
FAM177A1	family with sequence similarity 177, member A1	-0.75	-5.49	<0.0001	0.0012
GAS2L3	growth arrest-specific 2 like 3	-0.60	-5.57	<0.0001	0.0014
CTH	cystathionase (cystathionine gamma-lyase)	-0.60	-5.62	<0.0001	0.0013
OLR1	oxidized low density lipoprotein (lectin-like) receptor 1	-1.54	-5.72	<0.0001	0.0013
PLAU	plasminogen activator, urokinase	-0.63	-5.87	<0.0001	0.0011
C15orf48	chromosome 15 open reading frame 48	-1.51	-5.95	<0.0001	0.0011
NRIP3	nuclear receptor interacting protein 3	-1.04	-6.30	<0.0001	0.001

Table 88: Differentially expressed genes (vaccine study comparisons, DVC-LVS/USAMRIID-LVS (Tularemia) Day 7 vs. TIV (Influenza) Day 7). The average log_2 fold change was used to combine probe sets with the same gene symbol prior to the analysis. Results are sorted by descending t-statistic. Gene annotations were obtained from Affymetrix (HT HG-U133 Plus PM Annotations, Release 34,10/24/13).

Gene Name	Gene Description	Log_2 FC	t-Statistic	P	FDR
ERV3-2	endogenous retrovirus group 3, member 2	0.64	4.98	<0.0001	0.0142
GBP5	guanylate binding protein 5	0.92	4.85	<0.0001	0.0149

Gene Name	Gene Description	Log_2 FC	t-Statistic	P	FDR
GBP1P1	guanylate binding protein 1, interferon-inducible pseudogene 1	0.85	4.72	<0.0001	0.0187
GABARAPL3	GABA(A) receptors associated protein like 3, pseudogene	0.78	4.64	<0.0001	0.0187
GBP4	guanylate binding protein 4	0.64	4.43	<0.0001	0.0187
SERPING1	serpin peptidase inhibitor, clade G (C1 inhibitor), member 1	0.97	4.41	<0.0001	0.0187
OTTHUMG00000159342		0.69	4.32	<0.0001	0.0189
RP1-93H18.6		0.69	4.32	<0.0001	0.0189
GBP1	guanylate binding protein 1, interferon-inducible	0.87	3.83	0.0003	0.0478
FAM177A1	family with sequence similarity 177, member A1	-0.61	-4.06	0.0001	0.0317
OLR1	oxidized low density lipoprotein (lectin-like) receptor 1	-1.29	-4.18	0.0001	0.0276
CCRL2	chemokine (C-C motif) receptor-like 2	-0.69	-4.36	<0.0001	0.0229
PHLDA1	pleckstrin homology-like domain, family A, member 1	-0.77	-4.64	<0.0001	0.0187
C11orf96	chromosome 11 open reading frame 96	-0.64	-4.66	<0.0001	0.0187
CYB5D1	cytochrome b5 domain containing 1	-0.62	-5.12	<0.0001	0.0142
NRIP3	nuclear receptor interacting protein 3	-0.90	-5.22	<0.0001	0.0128
CYP1B1	cytochrome P450, family 1, subfamily B, polypeptide 1	-0.89	-5.23	<0.0001	0.0084
FKBP5	FK506 binding protein 5	-0.78	-5.44	<0.0001	0.007
LAMB3	laminin, beta 3	-0.99	-6.35	<0.0001	0.0018

Table 89: Differentially expressed genes (vaccine study comparisons, DVC-LVS/USAMRIID-LVS (Tularemia) Day 2 vs. LAIV (Influenza) Day 3). The average log_2 fold change was used to combine probe sets with the same gene symbol prior to the analysis. Results are sorted by descending t-statistic. Gene annotations were obtained from Affymetrix (HT HG-U133 Plus PM Annotations, Release 34,10/24/13).

Gene Name	Gene Description	Log_2 FC	t-Statistic	P	FDR
ST20	suppressor of tumorigenicity 20	0.62	4.73	<0.0001	0.0074
BTG3	BTG family, member 3	0.66	4.45	<0.0001	0.0195
CCR3	chemokine (C-C motif) receptor 3	0.78	4.40	<0.0001	0.0144
PTGS2	prostaglandin-endoperoxide synthase 2 (prostaglandin G/H synthase and cyclooxygenase)	1.47	4.19	0.0001	0.0203
TCN1	transcobalamin I (vitamin B12 binding protein, R binder family)	0.77	4.12	0.0001	0.0231
CLC	Charcot-Leyden crystal galectin	2.17	4.07	0.0003	0.0395
MXD1	MAX dimerization protein 1	0.89	3.90	0.0003	0.0399
CTB-167B5.2		0.85	3.89	0.0002	0.0321
OTTHUMG00000179513		0.85	3.89	0.0002	0.0321
GABARAPL3	GABA(A) receptors associated protein like 3, pseudogene	0.64	3.89	0.0004	0.0443
LOC100996457	uncharacterized LOC100996457	-0.73	-3.74	0.0004	0.0443
IL1A	interleukin 1, alpha	-0.68	-3.75	0.0005	0.0472
JUN	jun proto-oncogene	-0.96	-3.90	0.0003	0.0388
CLEC5A	C-type lectin domain family 5, member A	-0.69	-3.99	0.0002	0.0289
MIR155		-0.69	-4.03	0.0002	0.0263
MIR155HG		-0.69	-4.03	0.0002	0.0263

Gene Name	Gene Description	Log_2 FC	t-Statistic	P	FDR
F3	coagulation factor III (thromboplastin, tissue factor)	-0.63	-4.05	0.0002	0.0287
GPR84	G protein-coupled receptor 84	-0.79	-4.15	0.0001	0.021
ADORA2B	adenosine A2b receptor	-0.59	-4.33	<0.0001	0.0194
SLC5A6	solute carrier family 5 (sodium-dependent vitamin transporter), member 6	-0.59	-4.47	<0.0001	0.0127
ADORA2A		-0.59	-4.52	<0.0001	0.0123
SPECC1L-ADORA2A		-0.59	-4.52	<0.0001	0.0123
PRMT10	protein arginine methyltransferase 10 (putative)	-0.69	-4.53	<0.0001	0.0123
CYP1B1	cytochrome P450, family 1, subfamily B, polypeptide 1	-0.93	-4.55	<0.0001	0.0121
C11orf96	chromosome 11 open reading frame 96	-0.71	-4.69	<0.0001	0.0121
HELZ	helicase with zinc finger	-0.63	-4.69	<0.0001	0.0089
LGALS3	lectin, galactoside-binding, soluble, 3	-0.68	-4.81	<0.0001	0.0054
FAM177A1	family with sequence similarity 177, member A1	-0.71	-4.85	<0.0001	0.0054
GPR132	G protein-coupled receptor 132	-0.60	-4.91	<0.0001	0.0054
OLR1	oxidized low density lipoprotein (lectin-like) receptor 1	-1.44	-4.94	<0.0001	0.0054
MPHOSPH6	M-phase phosphoprotein 6	-0.75	-4.98	<0.0001	0.0054
PHLDA1	pleckstrin homology-like domain, family A, member 1	-0.94	-4.99	<0.0001	0.0062
C15orf48	chromosome 15 open reading frame 48	-1.38	-5.03	<0.0001	0.005
ABCD3	ATP-binding cassette, sub-family D (ALD), member 3	-0.60	-5.05	<0.0001	0.005
FNIP2	folliculin interacting protein 2	-0.69	-5.07	<0.0001	0.005
CYB5D1	cytochrome b5 domain containing 1	-0.69	-5.19	<0.0001	0.005
DCUN1D3	DCN1, defective in cullin neddylation 1, domain containing 3	-0.63	-5.24	<0.0001	0.0037
CCRL2	chemokine (C-C motif) receptor-like 2	-0.97	-5.26	<0.0001	0.005
LRRC32	leucine rich repeat containing 32	-0.60	-5.27	<0.0001	0.0041
PLAU	plasminogen activator, urokinase	-0.60	-5.47	<0.0001	0.0037
FKBP5	FK506 binding protein 5	-0.73	-5.47	<0.0001	0.0025
NRIP3	nuclear receptor interacting protein 3	-1.01	-5.77	<0.0001	0.0021
LAMB3	laminin, beta 3	-1.08	-6.03	<0.0001	0.0021

Table 90: Differentially expressed genes (vaccine study comparisons, DVC-LVS/USAMRIID-LVS (Tularemia) Day 7 vs. LAIV (Influenza) Day 7). The average log_2 fold change was used to combine probe sets with the same gene symbol prior to the analysis. Results are sorted by descending t-statistic. Gene annotations were obtained from Affymetrix (HT HG-U133 Plus PM Annotations, Release 34,10/24/13).

Probe Set ID	Gene Name	Description	Coefficient	Mean LFC Responders	Mean LFC Non-Responders	Ensembl Gene ID(s)	Reactome Pathway(s)
223767_PM_at	GPR84	G protein-coupled receptor 84	-0.5811	-0.88	-0.04	ENSG00000139572	
1565776_PM_at		Homo sapiens mRNA; cDNA DKFZp667M067 (from clone DKFZp667M067)	-0.5552	-1.12	-0.17		
227029_PM_at	FAM177A1	family with sequence similarity 177, member A1	0.3750	-0.59	-0.80	ENSG00000151327	

Probe Set ID	Gene Name	Description	Coefficient	Mean LFC Responders	Mean LFC Non-Responders	Ensembl Gene ID(s)	Reactome Pathway(s)
231779_PM_at	IRAK2	interleukin-1 receptor-associated kinase 2	-0.3229	-0.83	-0.30	ENSG00000134070	TRIF MEDIATED TLR3 SIGNALING; TAK1 ACTIVATES NFKB BY PHOSPHORYLATION AND ACTIVATION OF IKKS COMPLEX; MAP KINASE ACTIVATION IN TLR CASCADE; JNK C JUN KINASES PHOSPHORYLATION AND ACTIVATION MEDIATED BY ACTIVATED HUMAN TAK1; ACTIVATED TAK1 MEDIATES P38 MAPK ACTIVATION; SIGNALING BY ILS; IL1 SIGNALING; IRAK2 MEDIATED ACTIVATION OF TAK1 COMPLEX UPON TLR7 8 OR 9 STIMULATION; TRAF6 MEDIATED INDUCTION OF NFKB AND MAP KINASES UPON TLR7 8 OR 9 ACTIVATION; NFKB AND MAP KINASES ACTIVATION MEDIATED BY TLR4 SIGNALING REPERTOIRE; MYD88 MAL CASCADE INITIATED ON PLASMA MEMBRANE; INNATE IMMUNE SYSTEM; ACTIVATED TLR4 SIGNALLING; IMMUNE SYSTEM; TOLL RECEPTOR CASCADES; NOD1 2 SIGNALING PATHWAY; CYTOKINE SIGNALING IN IMMUNE SYSTEM; NUCLEOTIDE BINDING DOMAIN LEUCINE RICH REPEAT CONTAINING RECEPTOR NLR SIGNALING PATHWAYS
240103_PM_at	LOC100996457	uncharacterized LOC100996457	0.2576	-0.71	-1.27		
1564150_PM_a_at	C12orf79	chromosome 12 open reading frame 79	0.2122	-0.48	-0.96	ENSG00000257242	
212099_PM_at	RHOB	ras homolog family member B	-0.1781	0.49	0.86	ENSG00000143878	SIGNALING BY RHO GTPASES; DEVELOPMENTAL BIOLOGY; SIGNALING BY GPCR; GPVI MEDIATED ACTIVATION CASCADE; AXON GUIDANCE; G ALPHA1213 SIGNALLING EVENTS; GPCR DOWNSTREAM SIGNALING; SEMA4D IN SEMAPHORIN SIGNALING; SEMAPHORIN INTERACTIONS; SEMA4D INDUCED CELL MIGRATION AND GROWTH CONE COLLAPSE; HEMOSTASIS; PLATELET ACTIVATION SIGNALING AND AGGREGATION
AFFX-HUMISGF3A /M97935_MA_at	STAT1	signal transducer and activator of transcription 1, 91kDa	0.1235	0.90	0.61	ENSG00000115415	SIGNALING BY SCF KIT; GROWTH HORMONE RECEPTOR SIGNALING; ANTIVIRAL MECHANISM BY IFN STIMULATED GENES; SIGNALING BY FGFR IN DISEASE; SIGNALING BY FGFR1 MUTANTS; SIGNALING BY FGFR1 FUSION MUTANTS; SIGNALING BY FGFR MUTANTS; SIGNALING BY PDGF; DOWNSTREAM SIGNAL TRANSDUCTION; SIGNALING BY ILS; REGULATION OF IFNG SIGNALING; INTERFERON GAMMA SIGNALING; INTERFERON ALPHA BETA SIGNALING; REGULATION OF IFNA SIGNALING; INTERFERON SIGNALING; IL 6 SIGNALING; IMMUNE SYSTEM; CYTOKINE SIGNALING IN IMMUNE SYSTEM
217502_PM_at	IFIT2	interferon-induced protein with tetratricopeptide repeats 2	0.1118	0.91	0.45	ENSG00000119922	INTERFERON ALPHA BETA SIGNALING; INTERFERON SIGNALING; IMMUNE SYSTEM; CYTOKINE SIGNALING IN IMMUNE SYSTEM
226099_PM_at	ELL2	elongation factor, RNA polymerase II, 2	0.1084	-0.65	-0.85	ENSG00000118985	
236439_PM_at			-0.0797	0.62	0.94		
239661_PM_at			0.0769	0.73	0.39		
232383_PM_at	TFEC	transcription factor EC	-0.0677	0.69	1.21	ENSG00000105967	
231578_PM_at	GBP1	guanylate binding protein 1, interferon-inducible	0.0445	1.51	1.03	ENSG00000117228	INTERFERON GAMMA SIGNALING; INTERFERON SIGNALING; IMMUNE SYSTEM; CYTOKINE SIGNALING IN IMMUNE SYSTEM

Probe Set ID	Gene Name	Description	Coefficient	Mean LFC Responders	Mean LFC Non-Responders	Ensembl Gene ID(s)	Reactome Pathway(s)
203153_PM_at	IFIT1	interferon-induced protein with tetratricopeptide repeats 1	-0.0337	0.62	1.03	ENSG00000185745	ANTIVIRAL MECHANISM BY IFN STIMULATED GENES; INTERFERON ALPHA BETA SIGNALING; INTERFERON SIGNALING; IMMUNE SYSTEM; CYTOKINE SIGNALING IN IMMUNE SYSTEM
235175_PM_at	GBP4	guanylate binding protein 4	-0.0122	0.74	1.01	ENSG00000162654	INTERFERON GAMMA SIGNALING; INTERFERON SIGNALING; IMMUNE SYSTEM; CYTOKINE SIGNALING IN IMMUNE SYSTEM
207978_PM_s_at	NR4A3	nuclear receptor subfamily 4, group A, member 3	0.0068	-1.66	-1.88	ENSG00000119508	GENERIC TRANSCRIPTION PATHWAY; NUCLEAR RECEPTOR TRANSCRIPTION PATHWAY
227099_PM_s_at	C11orf96	chromosome 11 open reading frame 96	0.0033	-0.67	-0.72	ENSG00000187479	
214059_PM_at	IFI44	interferon-induced protein 44	-0.0022	0.53	0.83	ENSG00000137965	

Table 91: Combination of genes differentiating between responders and non-responders (Microagglutination titer, Day 2). Selected genes: 19 out of 117, minimum mean deviance: 1.14, mean misclassification error: 0.26). Sorted by absolute logistic regression coefficient. Probe Set annotations are based on Affymetrix HG-U133 Plus Perfect Match Release 34. Pathway annotations are based on Reactome classifications (MSigDB Version 4.0).

Probe Set ID	Gene Name	Description	Coefficient	Mean LFC Responders	Mean LFC Non-Responders	Ensembl Gene ID(s)	Reactome Pathway(s)
239567_PM_at			0.7756	-0.62	-0.99		
229437_PM_at	MIR155, MIR155HG	microRNA 155,MIR155 host gene (non-protein coding)	-0.6513	-0.80	-0.03	ENSG00000234883	
218851_PM_s_at	SFT2D3, WDR33	SFT2 domain containing 3,WD repeat domain 33	0.6415	-0.60	-1.11	ENSG00000136709	
227140_PM_at	INHBA	inhibin, beta A	-0.5637	-0.95	-0.15	ENSG00000122641	METABOLISM OF AMINO ACIDS AND DERIVATIVES; GLYCOPROTEIN HORMONES; PEPTIDE HORMONE BIOSYNTHESIS
223915_PM_at	BCOR	BCL6 corepressor	-0.4996	-0.80	-0.31	ENSG00000183337	
232383_PM_at	TFEC	transcription factor EC	-0.3052	0.65	1.17	ENSG00000105967	
1565776_PM_at		Homo sapiens mRNA; cDNA DKFZp667M067 (from clone DKFZp667M067)	-0.2787	-1.08	-0.03		
206374_PM_at	DUSP8	dual specificity phosphatase 8	-0.2656	-0.79	-0.40	ENSG00000184545	
1564093_PM_at	NEK1	NIMA-related kinase 1	0.2235	-0.57	-1.07	ENSG00000137601	
240238_PM_at			0.2087	0.84	0.04		
1557302_PM_at	ZNF585B	zinc finger protein 585B	0.2004	0.74	0.30	ENSG00000245680	GENERIC TRANSCRIPTION PATHWAY
234632_PM_x_at		Homo sapiens cDNA: FLJ22614 fis, clone HSI05089	0.1881	-0.52	-0.85		
230218_PM_at	HIC1	hypermethylated in cancer 1	-0.1260	-0.86	-0.26	ENSG00000177374	
212158_PM_at	SDC2	syndecan 2	-0.1224	-1.15	-0.45	ENSG00000169439	HS GAG DEGRADATION; CHONDROITIN SULFATE DERMATAN SULFATE METABOLISM; HS GAG BIOSYNTHESIS; HEPARAN SULFATE HEPARIN HS GAG METABOLISM; GLYCOSAMINOGLYCAN METABOLISM; A TETRASACCHARIDE LINKER SEQUENCE IS REQUIRED FOR GAG SYNTHESIS; METABOLISM OF CARBOHYDRATES
221563_PM_at	DUSP10	dual specificity phosphatase 10	-0.1012	-0.68	-0.42	ENSG00000143507	
204363_PM_at	F3	coagulation factor III (thromboplastin, tissue factor)	-0.0773	-0.82	-0.17	ENSG00000117525	FORMATION OF FIBRIN CLOT CLOTTING CASCADE; HEMOSTASIS
240024_PM_at	SEC14L2	SEC14-like 2 (S. cerevisiae)	-0.0702	-0.77	-0.21	ENSG00000100003	

Probe Set ID	Gene Name	Description	Coefficient	Mean LFC Responders	Mean LFC Non-Responders	Ensembl Gene ID(s)	Reactome Pathway(s)
238756_PM_at	GAS2L3	growth arrest-specific 2 like 3	-0.0544	-0.97	-0.43	ENSG00000139354	
204103_PM_at	CCL4	chemokine (C-C motif) ligand 4	-0.0543	-0.85	-0.28	ENSG00000275302	
212659_PM_s_at	IL1RN	interleukin 1 receptor antagonist	-0.0429	-0.92	-0.36	ENSG00000136689	SIGNALING BY ILS; IL1 SIGNALING; IMMUNE SYSTEM; CYTOKINE SIGNALING IN IMMUNE SYSTEM
207850_PM_at	CXCL3	chemokine (C-X-C motif) ligand 3	-0.0408	-1.33	-0.38	ENSG00000163734	SIGNALING BY GPCR; PEPTIDE LIGAND BINDING RECEPTORS; CLASS A1 RHODOPSIN LIKE RECEPTORS; CHEMOKINE RECEPTORS BIND CHEMOKINES; GPCR DOWNSTREAM SIGNALING; G ALPHA I SIGNALLING EVENTS; GPCR LIGAND BINDING
244026_PM_at			0.0345	-0.51	-1.04		
201490_PM_s_at	PPIF	peptidylprolyl isomerase F	-0.0333	-0.94	-0.50	ENSG00000108179	
208868_PM_s_at	GABARAPL1	GABA(A) receptor-associated protein like 1	0.0325	-0.60	-0.78	ENSG00000139112	
202589_PM_at	TYMS	thymidylate synthetase	0.0316	0.77	0.43	ENSG00000176890	CELL CYCLE; CELL CYCLE MITOTIC; METABOLISM OF NUCLEOTIDES; G1 S TRANSITION; MITOTIC G1 G1 S PHASES; E2F MEDIATED REGULATION OF DNA REPLICATION; G1 S SPECIFIC TRANSCRIPTION; PYRIMIDINE METABOLISM
236402_PM_at	BRAF	v-raf murine sarcoma viral oncogene homolog B1	0.0190	-0.69	-0.79	ENSG00000157764	SIGNALLING BY NGF; SPRY REGULATION OF FGF SIGNALING; NEGATIVE REGULATION OF FGFR SIGNALING; ARMS MEDIATED ACTIVATION; PROLONGED ERK ACTIVATION EVENTS; NGF SIGNALLING VIA TRKA FROM THE PLASMA MEMBRANE; SIGNALLING TO ERKS; SIGNALING BY FGFR IN DISEASE; SIGNALLING TO P38 VIA RIT AND RIN; TRANSMISSION ACROSS CHEMICAL SYNAPSES; NEURONAL SYSTEM; NEUROTRANSMITTER RECEPTOR BINDING AND DOWNSTREAM TRANSMISSION IN THE POSTSYNAPTIC CELL; ACTIVATION OF NMDA RECEPTOR UPON GLUTAMATE BINDING AND POSTSYNAPTIC EVENTS; CREB PHOSPHORYLATION THROUGH THE ACTIVATION OF RAS; POST NMDA RECEPTOR ACTIVATION EVENTS; SIGNALING BY FGFR
1556499_PM_s_at	COL1A1	collagen, type I, alpha 1	-0.0126	-1.01	-0.54	ENSG00000108821	DEVELOPMENTAL BIOLOGY; EXTRACELLULAR MATRIX ORGANIZATION; CELL SURFACE INTERACTIONS AT THE VASCULAR WALL; COLLAGEN FORMATION; PLATELET ADHESION TO EXPOSED COLLAGEN; INTEGRIN CELL SURFACE INTERACTIONS; SIGNALING BY PDGF; GPVI MEDIATED ACTIVATION CASCADE; AXON GUIDANCE; NCAM1 INTERACTIONS; NCAM SIGNALING FOR NEURITE OUT GROWTH; HEMOSTASIS; PLATELET ACTIVATION SIGNALING AND AGGREGATION
206173_PM_x_at	GABPB1	GA binding protein transcription factor, beta subunit 1	0.0114	-0.63	-0.84	ENSG00000104064	
237496_PM_at		ESTs, Weakly similar to ALU7HUMAN ALU SUBFAMILY SQ SEQUENCE CONTAMINATION WARNING ENTRY (H.sapiens)	0.0103	-0.69	-0.78		
223767_PM_at	GPR84	G protein-coupled receptor 84	-0.0101	-1.11	-0.31	ENSG00000139572	
202014_PM_at	PPP1R15A	protein phosphatase 1, regulatory subunit 15A	-0.0076	-0.90	-0.33	ENSG00000087074	DOWNREGULATION OF TGF BETA RECEPTOR SIGNALING; TGF BETA RECEPTOR SIGNALING ACTIVATES SMADS; SIGNALING BY TGF BETA RECEPTOR COMPLEX

Probe Set ID	Gene Name	Description	Coefficient	Mean LFC Responders	Mean LFC Non-Responders	Ensembl Gene ID(s)	Reactome Pathway(s)

Table 92: Combination of genes differentiating between responders and non-responders (Microagglutination titer, Day 7). Selected genes: 31 out of 219, minimum mean deviance: 0.96, mean misclassification error: 0.21). Sorted by absolute logistic regression coefficient. Probe Set annotations are based on Affymetrix HG-U133 Plus Perfect Match Release 34. Pathway annotations are based on Reactome classifications (MSigDB Version 4.0).

Study Visit	Treatment Group	N	Gene Variables	IMO Variables	CV	Canonical Correlation (C1)	Canonical Correlation (C2)	Mean Explained Variance (Gene Variables, C1+C2)	Mean Explained Variance (IMO Variables, C1+C2)
Day 1	Combined Study Groups	37	53	5	0.69	0.66	0.42	0.33	0.72
Day 2	Combined Study Groups	38	117	5	0.30	0.60	0.49	0.23	0.70
Day 7	Combined Study Groups	38	219	5	0.14	0.99	0.98	0.04	0.75
Day 14	Combined Study Groups	39	291	5	0.27	0.89	0.87	0.08	0.71

Table 93: Canonical correlation analysis summary statistics

Figures

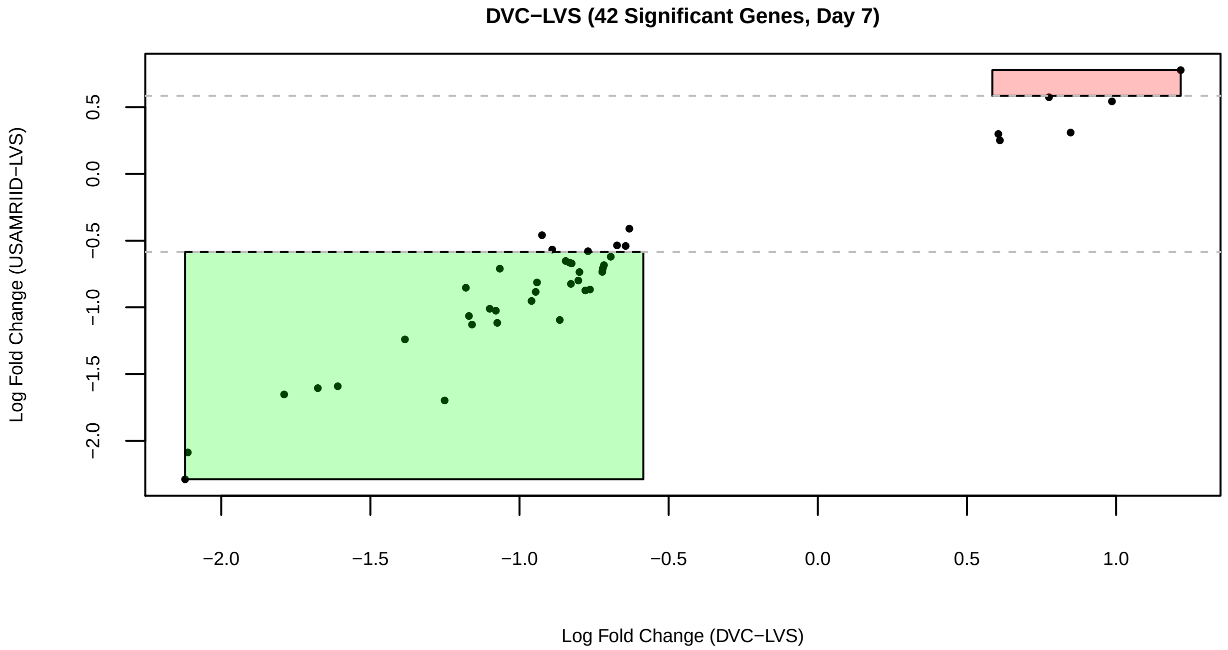

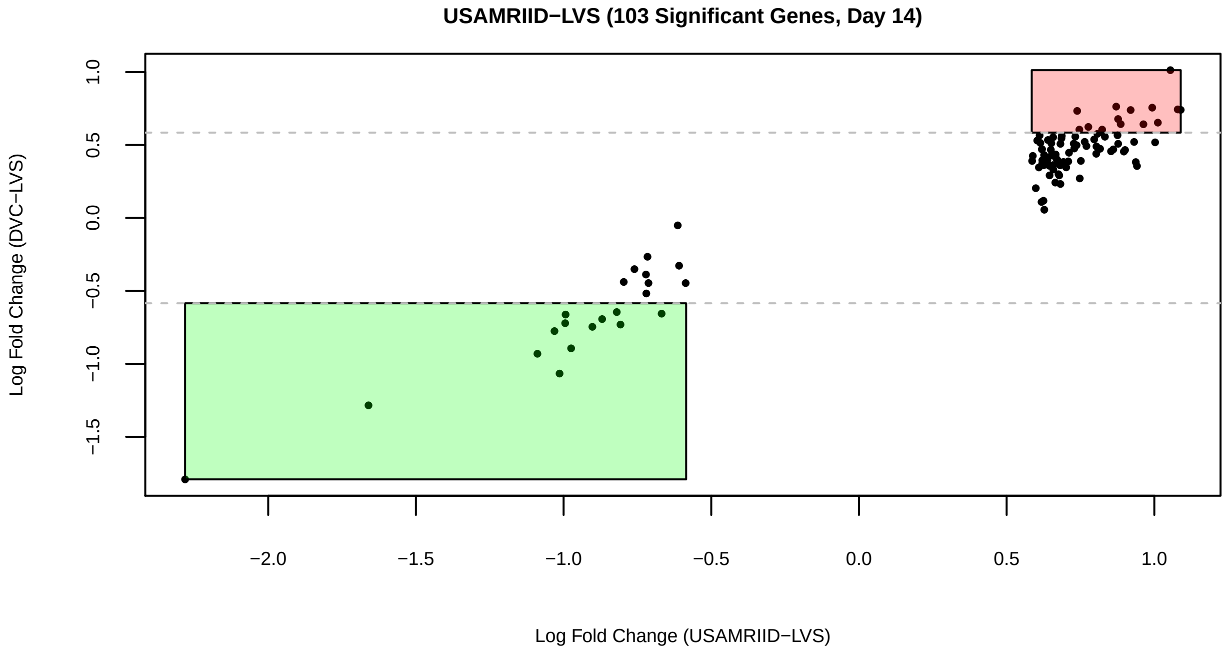

Figure 1: Scatter plot of fold changes of significant genes (DVC-LVS vs. USAMRIID-LVS, Day 7, 14). Fold change region in which both study groups exceed the upper fold change cut off is shaded in red. Fold change region in which both study groups are below the lower fold change cut off is shaded in green

Gene cluster dendrogram with bootstrap probabilities (Combined Study Groups, Day 1−14)

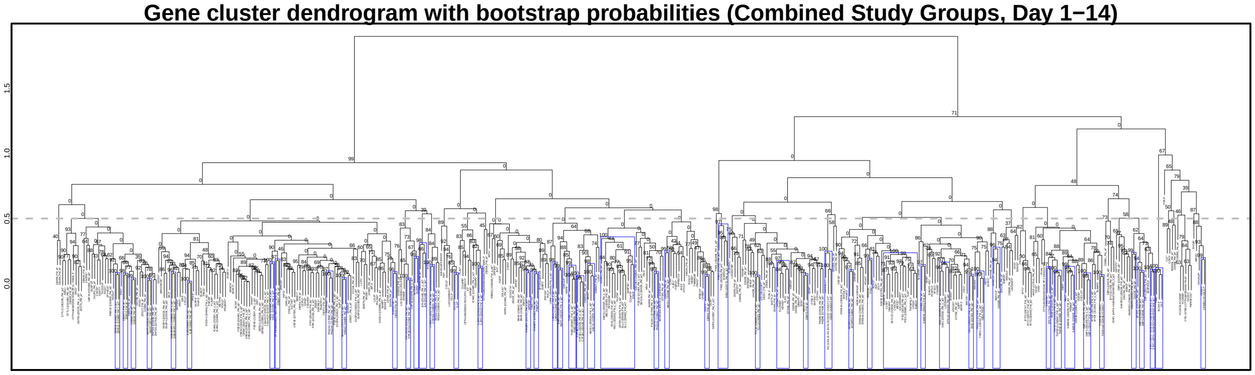

Figure 2: Gene cluster dendrogram with bootstrap probabilities (Combined Study Groups, Day 1-14). The y axis shows the distance at which clusters were formed by the complete linkage clustering algorithm based on uncentered Pearson correlation distance between log fold changes. Multiscale bootstrap probabilities are shown at each branch intersection. Significant clusters are highlighted in blue.

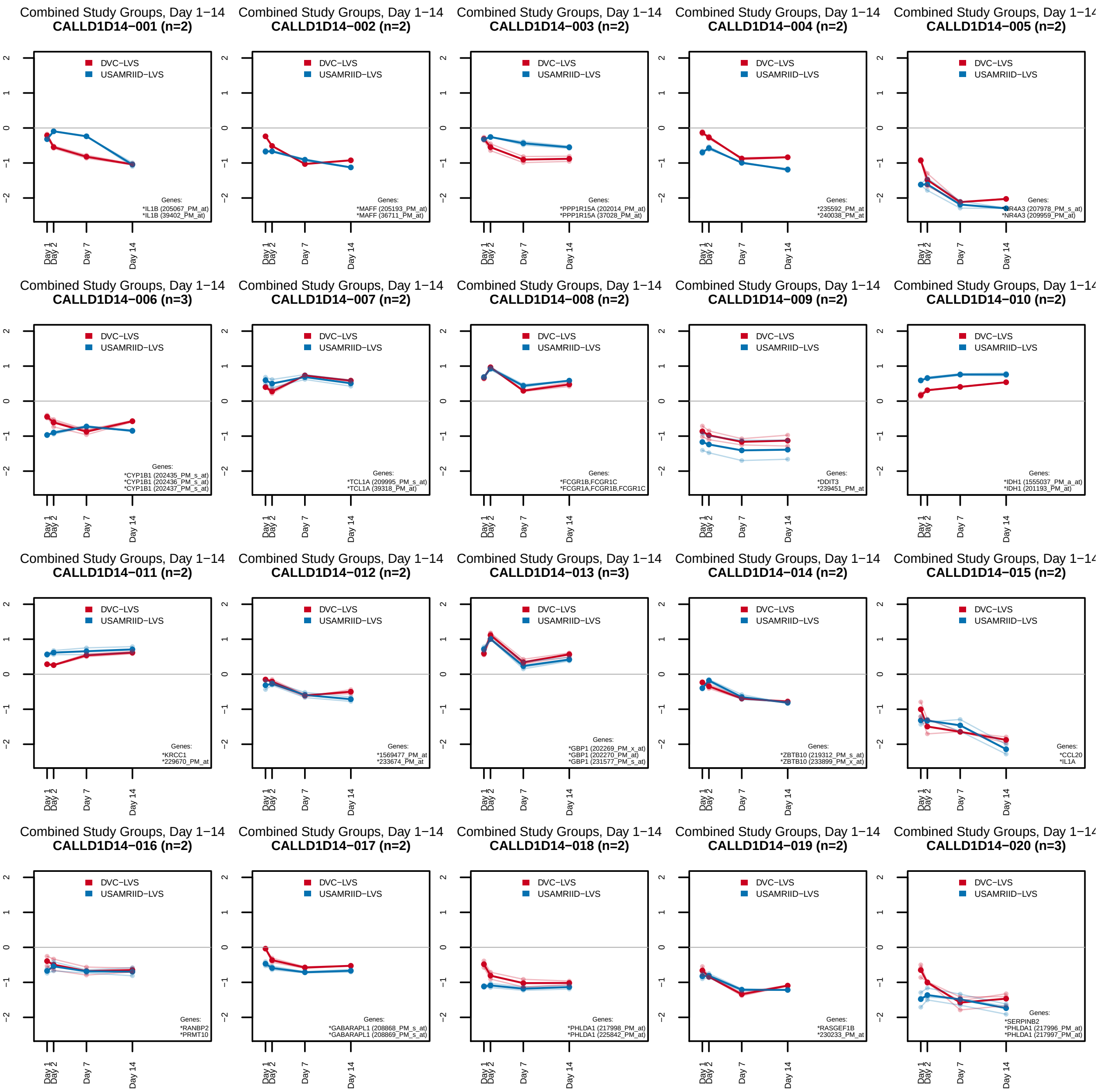

Figure 3: Gene cluster time trends of baseline log_2 fold change by study group (Combined Study Groups, Day 1-14). Header indicates cluster ID. Mean log_2 fold change across cluster genes is drawn in bold. Individual mean gene log_2 fold changes are plotted in lighter colors.

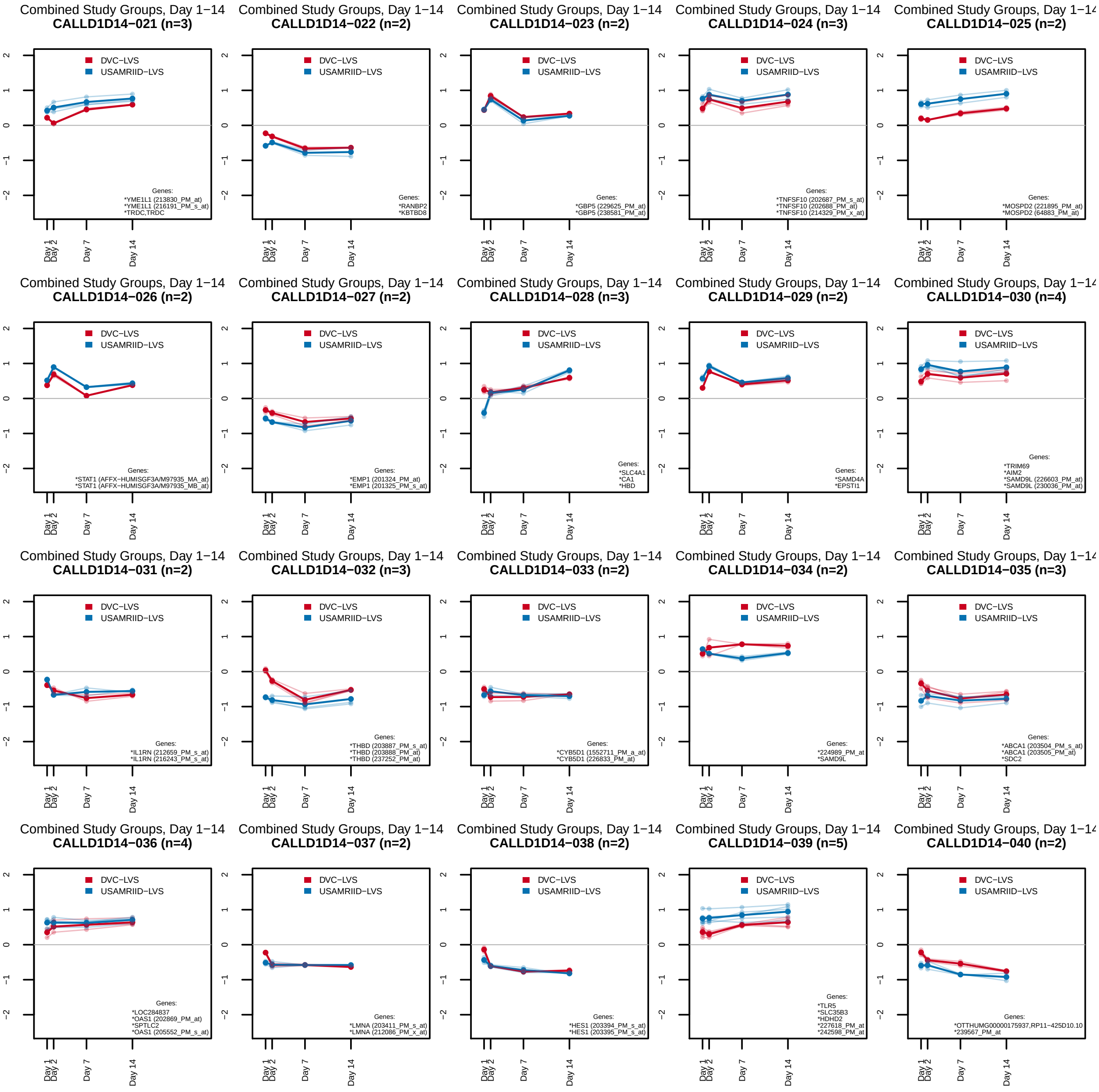

Figure 4: Gene cluster time trends of baseline log_2 fold change by study group (Combined Study Groups, Day 1-14). Header indicates cluster ID. Mean log_2 fold change across cluster genes is drawn in bold. Individual mean gene log_2 fold changes are plotted in lighter colors.

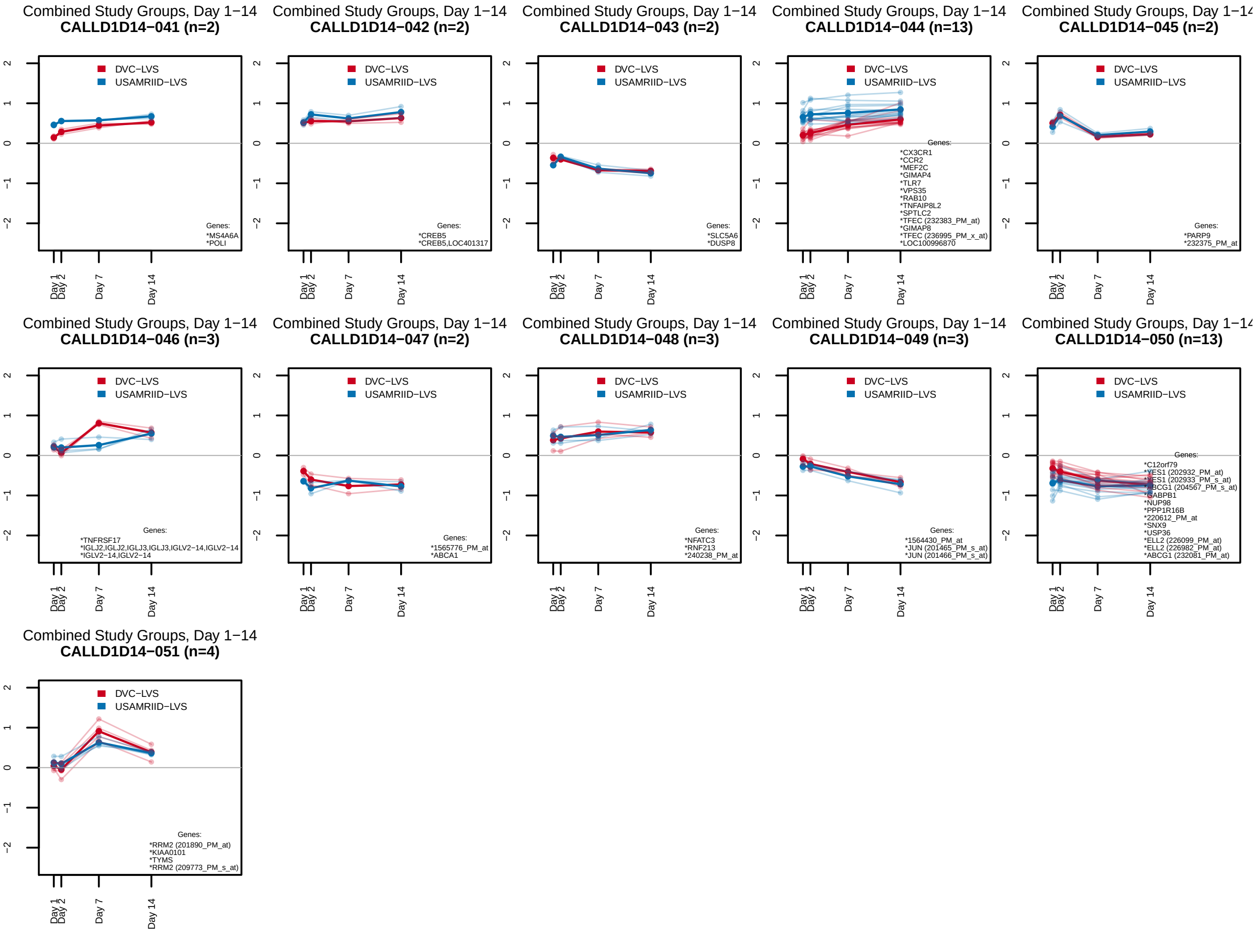

Figure 5: Gene cluster time trends of baseline log_2 fold change by study group (Combined Study Groups, Day 1-14). Header indicates cluster ID. Mean log_2 fold change across cluster genes is drawn in bold. Individual mean gene log_2 fold changes are plotted in lighter colors.

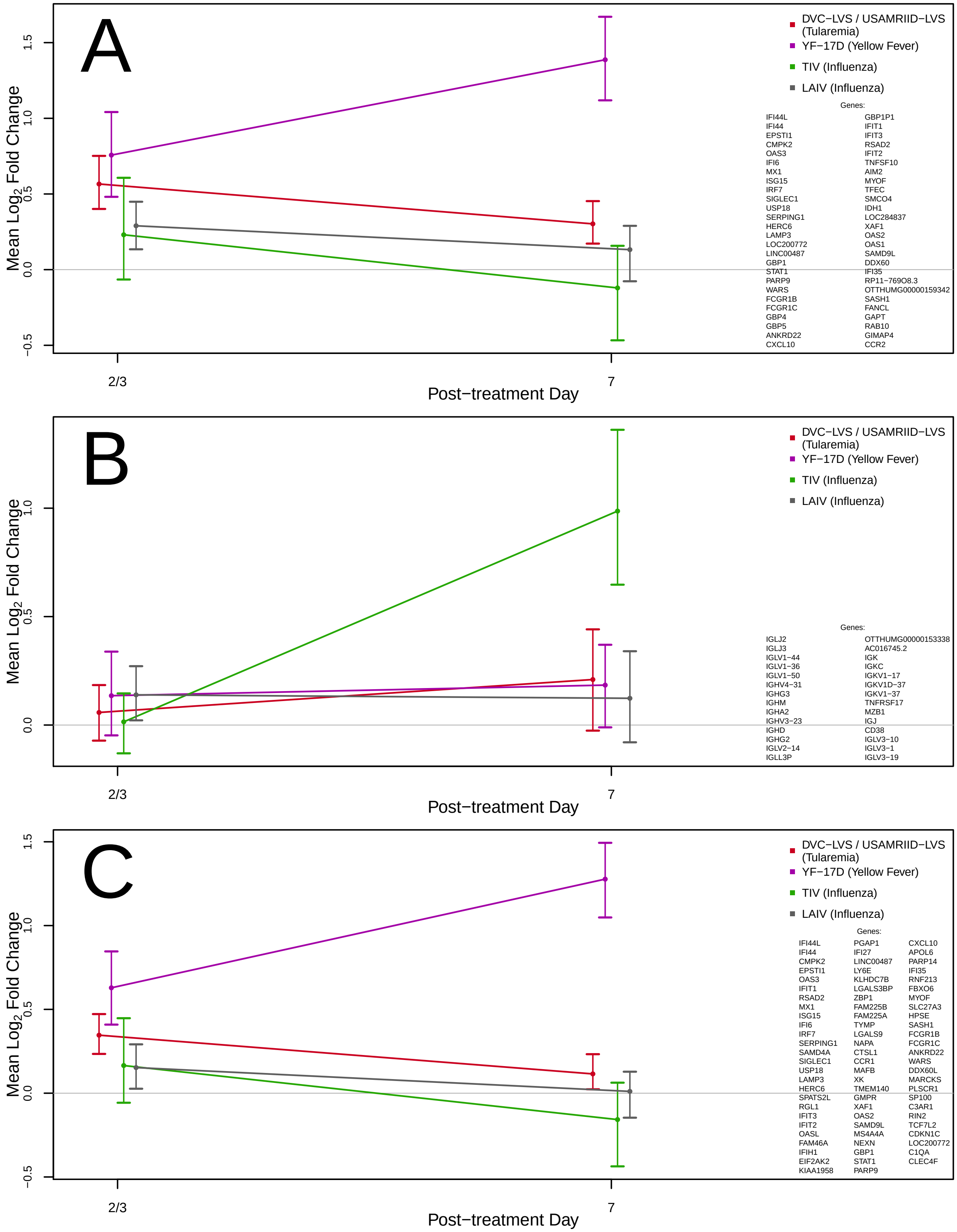

Figure 6: Time trends of mean log_2 fold change and associated 95% bootstrap confidence intervals for gene clusters identified in subject-level heatmaps.

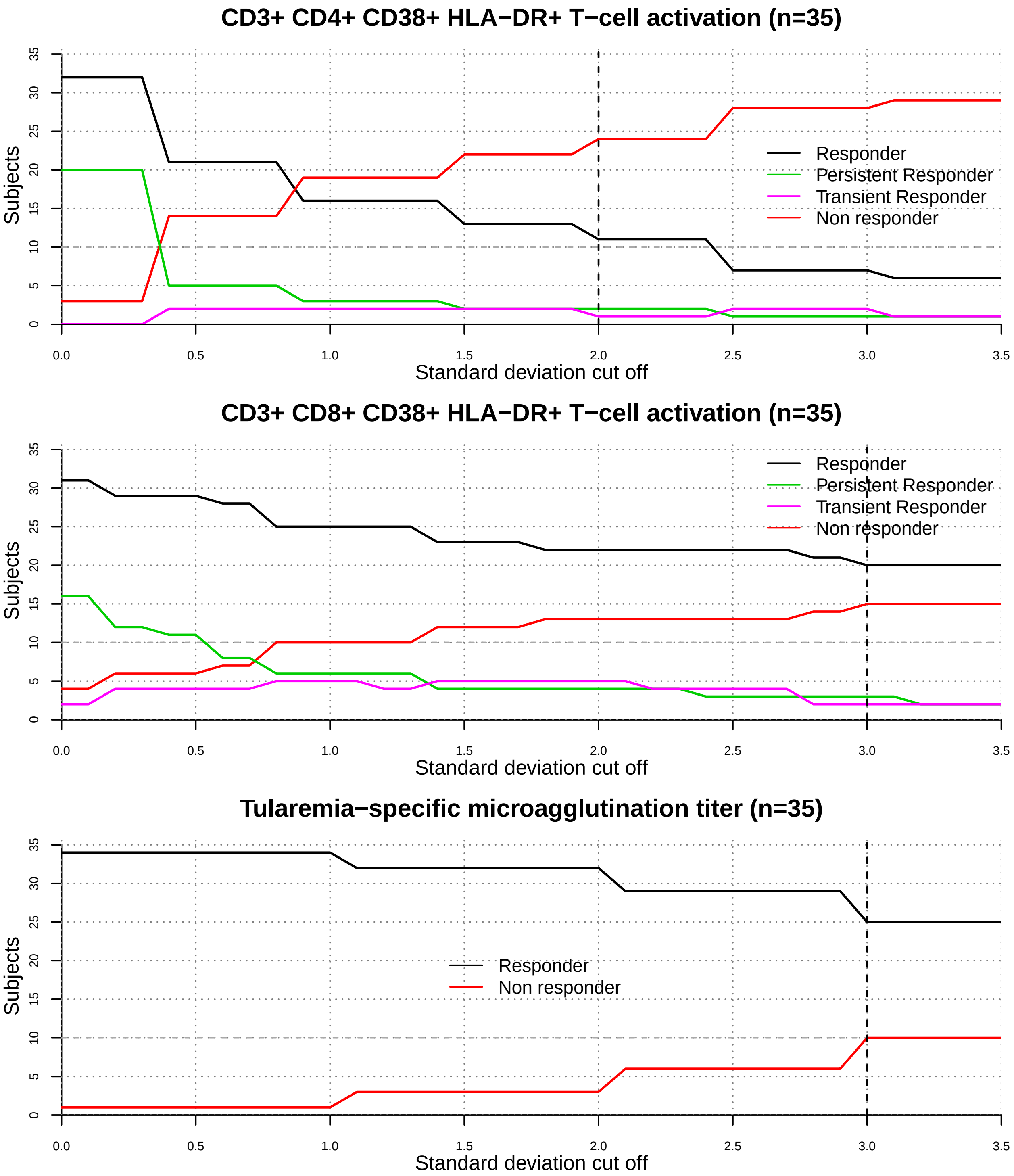

Figure 7: Number of subjects with microarray data for all post-vaccination days that pass a certain immune response cut off by assay. Y-axis: number of subjects, x-axis: the number of standard deviations above the baseline mean used to define responders (see Section 2.8 for details). The dashed black vertical line indicates the standard deviation cut off that was used for the analysis.

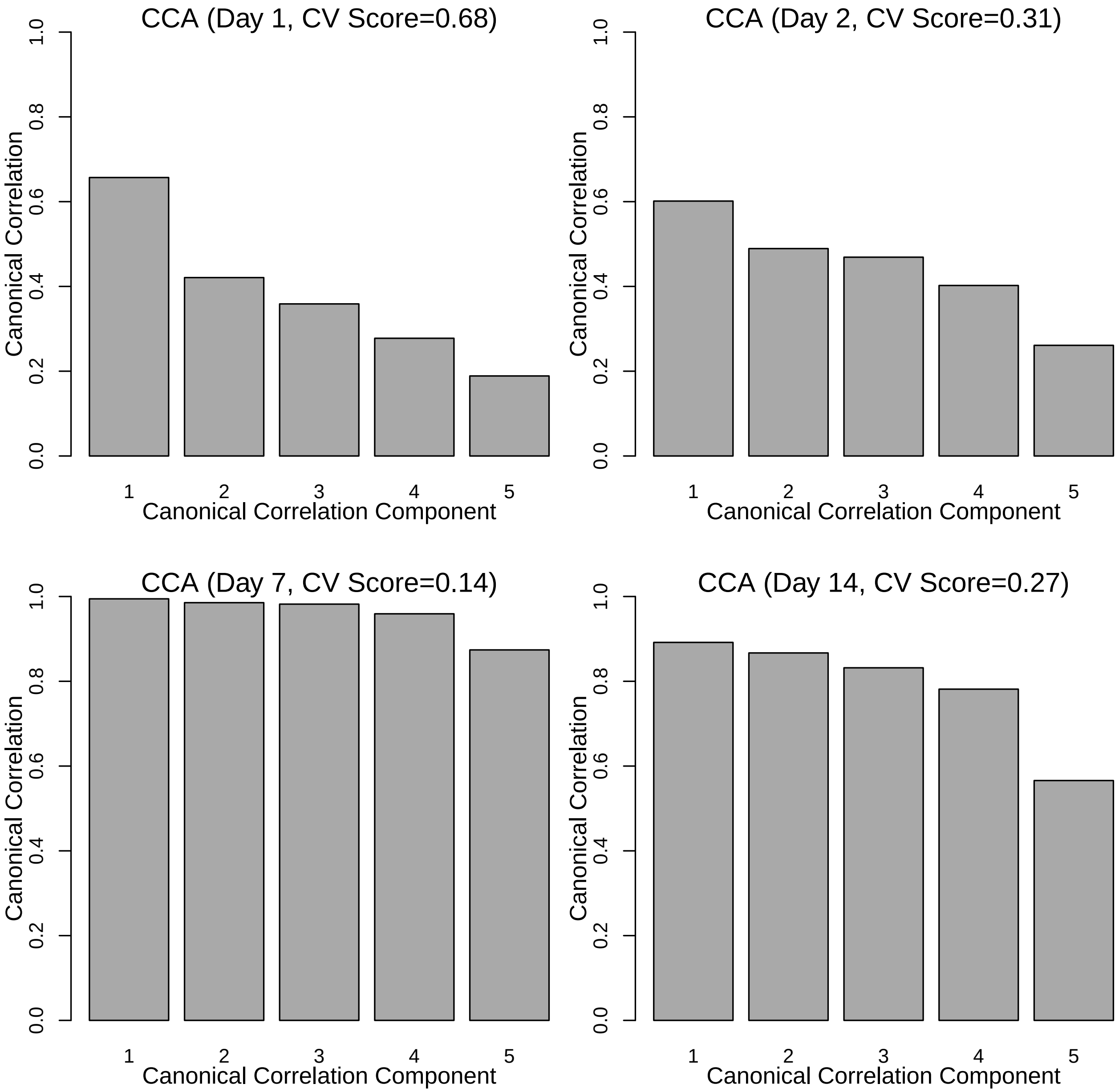

Figure 8: Canonical correlation scree plots for combined study groups. CV-score represents cross validation score.

4 References

[1] Mulligan, Mark J., et al. "Tularemia vaccine: Safety, reactogenicity,"'Take"' skin reactions, and antibody responses following vaccination with a new lot of the Francisella tularensis live vaccine strain-A phase 2 randomized clinical Trial." Vaccine 35.36 (2017): 4730-4737.

[2] Johnson, W. Evan, Cheng Li, and Ariel Rabinovic. "Adjusting batch effects in microarray expression

data using empirical Bayes methods." Biostatistics 8.1 (2007): 118-127.

[3] Subramanian, Aravind, et al. "Gene set enrichment analysis: a knowledge-based approach for interpreting genome-wide expression profiles." Proceedings of the National Academy of Sciences 102.43 (2005): 15545-15550.

[4] Kanehisa, Minoru, and Susumu Goto. "KEGG: kyoto encyclopedia of genes and genomes." Nucleic acids research 28.1 (2000): 27-30.

[5] Liberzon, Arthur, et al. "Molecular signatures database (MSigDB) 3.0." Bioinformatics 27.12 (2011): 1739-1740.
